# Single Mutation on Trastuzumab Modulates the Stability of Antibody–Drug Conjugates Built Using Acetal-Based Linkers and Thiol-Maleimide Chemistry

**DOI:** 10.1021/jacs.1c07675

**Published:** 2022-03-16

**Authors:** Xhenti Ferhati, Ester Jiménez-Moreno, Emily A. Hoyt, Giulia Salluce, Mar Cabeza-Cabrerizo, Claudio D. Navo, Ismael Compañón, Padma Akkapeddi, Maria J. Matos, Noelia Salaverri, Pablo Garrido, Alfredo Martínez, Víctor Laserna, Thomas V. Murray, Gonzalo Jiménez-Osés, Peter Ravn, Gonçalo J. L. Bernardes, Francisco Corzana

**Affiliations:** † Departamento de Química, Centro de Investigación en Síntesis Química, Universidad de La Rioja, 26006 Logroño, Spain; ‡ Yusuf Hamied Department of Chemistry, University of Cambridge, Lensfield Road, CB2 1EW Cambridge, U.K.; § Center for Cooperative Research in Biosciences (CIC BioGUNE), Basque Research and Technology Alliance (BRTA), Bizkaia Technology Park, Building 800, 48160 Derio, Spain; ⊥ Instituto de Medicina Molecular João Lobo Antunes, Faculdade de Medicina, Universidade de Lisboa, Av. Prof. Egas Moniz, 1649-028 Lisboa, Portugal; # Angiogenesis Group, Oncology Area, Center for Biomedical Research of La Rioja (CIBIR), 26006 Logroño, Spain; ¶ Biologics Engineering, R&D, Astra Zeneca, CB21 6GH Cambridge, U.K.; ∥ Ikerbasque, Basque Foundation for Science, 48013 Bilbao, Spain

## Abstract

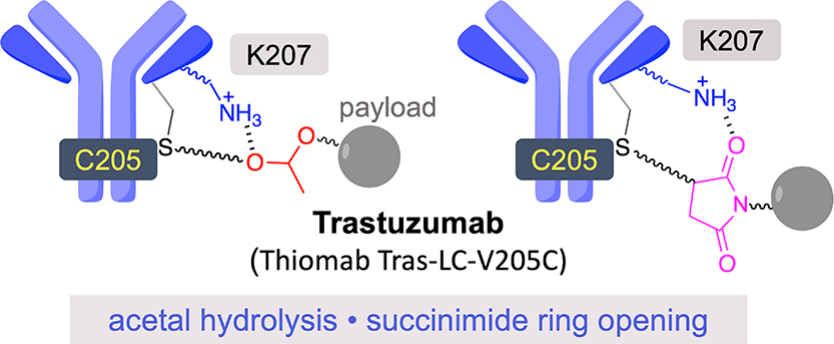

Antibody-drug conjugates (ADCs) are a class of targeted therapeutics used to selectively kill cancer cells. It is important that they remain intact in the bloodstream and release their payload in the target cancer cell for maximum efficacy and minimum toxicity. The development of effective ADCs requires the study of factors that can alter the stability of these therapeutics at the atomic level. Here, we present a general strategy that combines synthesis, bioconjugation, linker technology, site-directed mutagenesis, and modeling to investigate the influence of the site and microenvironment of the trastuzumab antibody on the stability of the conjugation and linkers. Trastuzumab is widely used to produce targeted ADCs because it can target with high specificity a receptor that is overexpressed in certain breast cancer cells (HER2). We show that the chemical environment of the conjugation site of trastuzumab plays a key role in the stability of linkers featuring acid-sensitive groups such as acetals. More specifically, Lys-207, located near the reactive Cys-205 of a thiomab variant of the antibody, may act as an acid catalyst and promote the hydrolysis of acetals. Mutation of Lys-207 into an alanine or using a longer linker that separates this residue from the acetal group stabilizes the conjugates. Analogously, Lys-207 promotes the beneficial hydrolysis of the succinimide ring when maleimide reagents are used for conjugation, thus stabilizing the subsequent ADCs by impairing the undesired retro-Michael reactions. This work provides new insights for the design of novel ADCs with improved stability properties.

## Introduction

Antibody-drug conjugates (ADCs) are a class of targeted therapeutics currently used for the selective destruction of cancer cells.^1−3^ Most of these conjugates are prepared by linking potent cytotoxic agents or other functional components through a variety of linkers to cysteine or lysine residues on the antibody.^4^ Ideally, ADCs should remain intact in the bloodstream and efficiently release their payload in the target cell for maximum efficacy and minimal toxicity. Similarly, it needs to be considered that the inherent properties of the antibody, together with the conjugation chemistry, the linker, and the cytotoxic molecule used, strongly influence the drug-like properties of the resulting conjugates and their stability.^5^ It is known that reactive thiols present in plasma molecules, for example, in glutathione or Cys-34 in albumin, may react with the conjugates and reduce their stability in vivo.^6,7^ In the context of artificial enzymes, for example, it is well known that the engineering of proximal amino acids in the active site can affect their activity significiantly.^8,9^ Similarly, it is known that the chemical and steric environment of the conjugation site is known to modulate both the conjugation and deconjugation properties of the linkers and thus, in the latter case, their stability and efficacy.^10,11^ Vollmar et al. reported that modulation of the p*K*_a_ of the thiol of a cysteine residue could affect the stability of ADCs. Specifically, conjugates that present a higher thiol p*K*_a_ yielded more stable conjugates.^12^ On the other hand, the steric influence on the stability of conjugates attached by disulfide chemistry was studied by Steiner et al. Derivatives with longer linkers, and thus less steric hindrance, were reported to be more prone to reduction and concomitant drug release, resulting in a decreased stability of the conjugates.^13^ The site of conjugation has also been reported to affect the extent of off-target cleavage of valine-citrulline linkers by serum proteases.^11^

**Figure 1:**
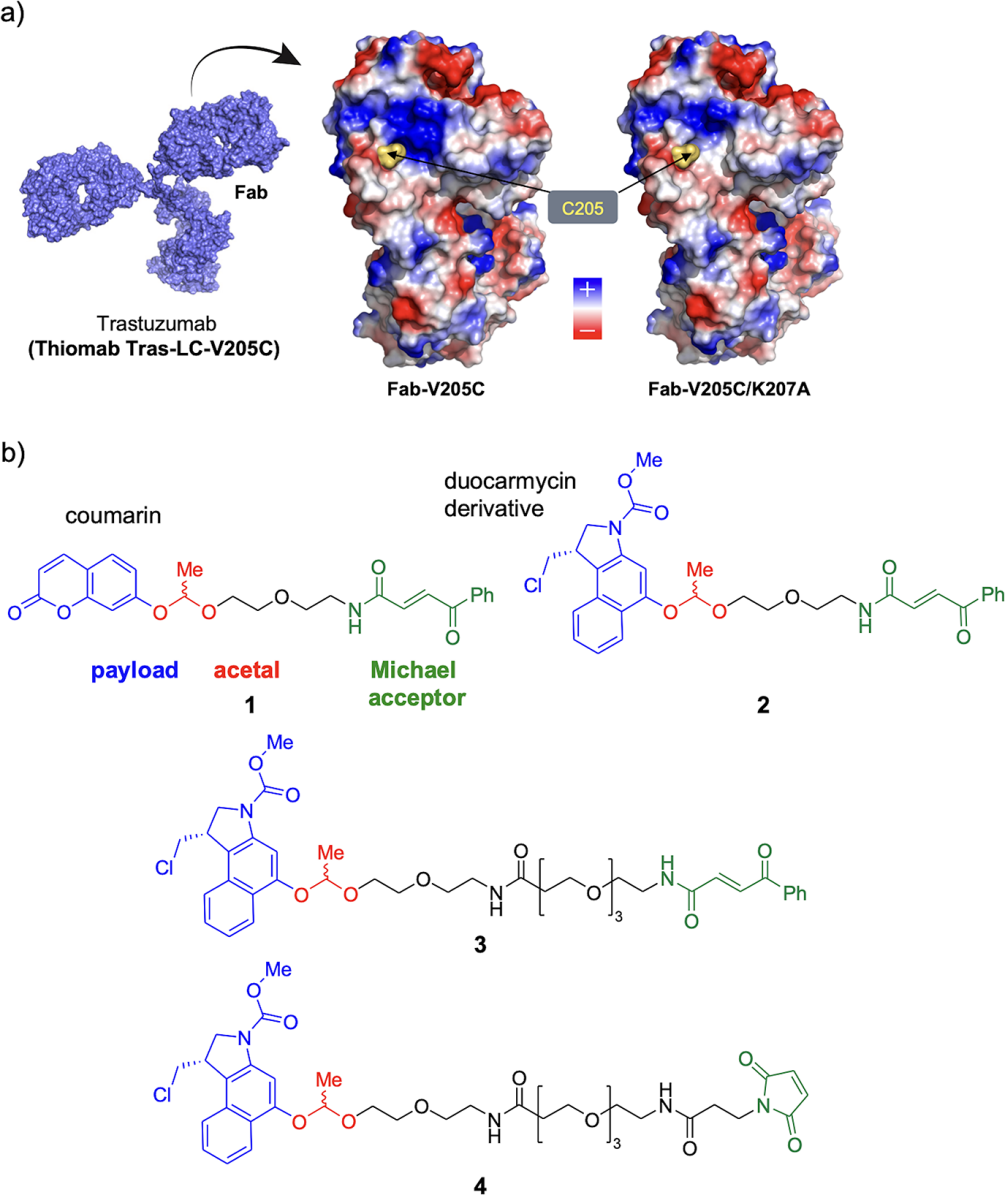
(a) Electrostatic potential surface of the Fab region of a thiomab derived from trastuzumab (Thiomab Tras-LC-V205C) and the corresponding mutant (Fab-V205C/K207A). (b) Linkers studied in this work were based on acetals.

The influence of the chemical environment and its local charge have also been found of significant importance for thiomab antibodies modified by maleimide chemistry. In this context, thiomabs are variants of trastuzumab (Herceptin, Figure 1a), a widely used therapeutic antibody targets the HER2 receptor, which is overexpressed in 20–30% of human breast cancers, and correlates with more aggressive tumors and a poorer prognosis.^14^ These variants include engineered cysteines at selected positions in order to provide for reactive tags for bioconjugation. Interestingly, it has been reported that the incorporation of a reactive cysteine residue at a site with a positively charged environment (e.g., with a high density of lysine residues) in Thiomab LC-V205C^15^ can facilitate the hydrolysis of the succinimide ring formed during conjugation with maleimide, preventing the retro-Michael reaction and stabilizing the conjugate.^10^ Development of self-hydrolyzing maleimides that include a basic amino-group adjacent to the maleimide moiety also points to the importance of the positively charged environment in this stabilization.^16^ However, a specific mechanism of how the conjugation site in Thiomab LC-V205C facilitates this hydrolysis, and thus stabilizes the conjugates, remains unclear.

We hypothesized that the stability of conjugates with a linker based on an acid labile group, such as acetals, which can undergo hydrolysis under acidic catalysis, could also be affected by the conjugation site environment of Thiomab LC-V205C. Ketals have been widely used for the controlled release of drugs, for example, the release of nucleic acids,^17,18^ the design of degradable polymers,^19,20^ and for prodrug formulations with particles.^21,22^ Acetals have also been used as acid-sensitive groups to generate prodrugs that exhibit enhanced absorption^23^ or circulating half-life.^24,25^ However, although some significant examples have been reported till date,^26,27^ the study of ADCs with acetal linkers remains largely unexplored. In addition, how the conjugation site can affect this type of motifs has not been previously investigated.

In this work, we uncover the role of Lys-207, which is in close proximity to the reactive Cys-205 of this thiomab, in the stability of conjugates that have such linkers based on an acid-labile group, either acetals or a maleimide scaffold for conjugation.

## Results and Discussion

**Figure 2:**
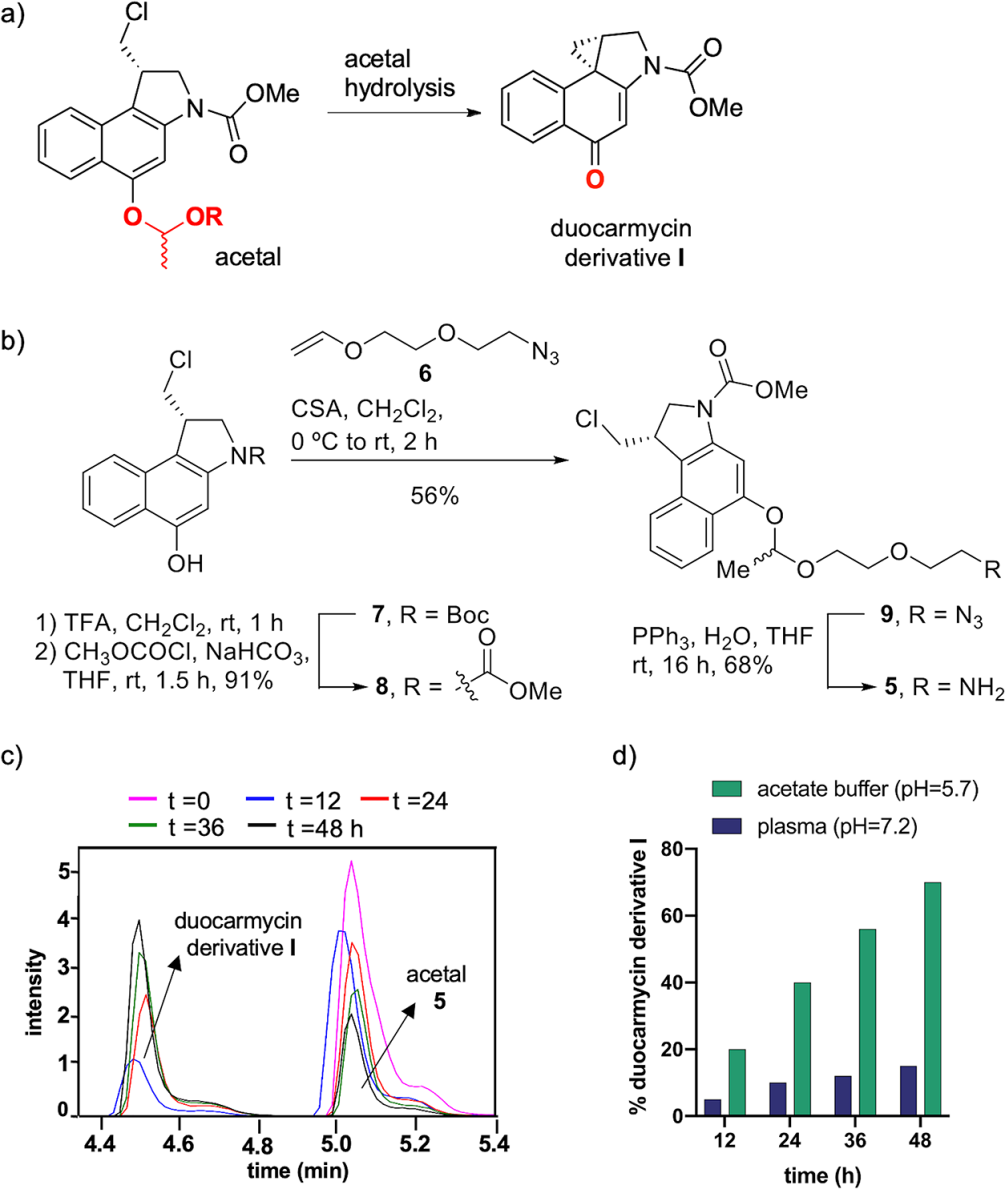
(a) Formation of duocarmycin derivative **I** after hydrolysis of the acetal group. (b) Synthetic route to prepare acetal **5**. (c) Monitoring of duocarmycin derivative **I** from acetal **5** at acidic pH [NaP_i_ buffer (0.1 M, pH = 5.7)] and 37 °C by UPLC-MS. (d) Stability of acetal **5** at different pH values.

We started our work by conjugating a prodrug and a masked fluorophore (see above) to Thiomab Tras-LC-V205C (hereafter referred to as IgG) through acetal-conditionally labile linkers derived from acetaldehyde, which are easy to prepare as a racemic mixture (Figure 1b, Schemes S1–S5, Figures S1–S22). These linkers contain a short ethylene glycol-based chain to increase hydrophilicity, a key aspect in the ADC design,^28^ and are equipped with a handle for Cys-selective bioconjugation to the antibody. We used a carbonylacrylamide^29,30^ or a typical maleimide^31^ to conjugate the linkers to the free engineered cysteine present in the antibody. They also contain a coumarin (acetal **1**, Figure 1b) whose fluorescence is greatly attenuated when it is part of the linker, or a prodrug derivative of duocarmycin (acetal **2**, Figure 1b) that is converted to its active analogue (derivative **I**, Figure 2a) upon acidic pH-induced acetal cleavage. In general, natural duocarmycins show potent cytotoxic activity as a DNA-alkylating agents and are suitable for destroying solid tumors.^32^ These derivatives have additional subunits compared with duocarmycin derivative **I** that can interact with the minor groove of DNA via hydrogen bonds and CH/π interactions, resulting in very high cytotoxicity.^33−37^ Some of them show IC_50_ values of around 20 pM in the leukemia cell line L1210.^38^ Derivative **I** lacks the indole motif and although being relatively toxic,^39,40^ the IC_50_ for the same cell line is much higher, with a value of 0.14 μM. Yet, this reduced version of duocarmycin was chosen because this scaffold provides easy access to the acetal derivatives studied in this work (compounds **2–4**). The free naphthol group of this duocarmycin derivative has a fundamental role in toxicity, which promotes an intramolecular spiro-cyclization under physiological conditions to generate the active and cytotoxic drug^38^ (Figure 2a). Therefore, blocking this reactivity through the conditional protection of the naphthol group has been explored by several groups as a strategy for controlled drug activation.^41−46^ In this work, we design a duocarmycin prodrug by blocking the hydroxyl group with an acetal. Thus, the release of the alcohol, and the subsequent transformation into compound **I**, will occur in situ after treatment of the linker under acidic conditions. The mechanism of such a reaction was analyzed by quantum mechanical calculations, which demonstrated the importance of phenol deprotonation to promote prodrug activation (Scheme S6, Figure S23, Tables S1 and S2). To test the stability of acetals **1** and **2** at neutral and acidic pH, we prepared their structurally simpler variants by removing the reactive Michael acceptor fragment. Acetal **5** was synthesized as a diastereomeric mixture via the synthetic pathway, as shown in Figure 2b and Scheme S2, in an overall yield of 35%. The pseudo-first-order hydrolysis rate constant (*k*_1_) of acetal **5** was determined by ultra-performance liquid chromatography–mass spectrometry (UPLC-MS; Figures 2c and S31, Table S9). The value of this constant (*k*_1_ = 5.0 × 10^–6^ ± 0.8 × 10^–6^ s^–1^ at pH = 5.7 and 37 °C) was similar to that of the variant with coumarin (Figure S32), suggesting that these substituents (payloads) have little effect on the rate of hydrolysis. Interestingly, the acetal-coupled prodrug was stable in plasma and underwent ∼10% hydrolysis in 24 h (Figure S33), whereas 40% of the acetal was hydrolyzed in the same period under slightly acidic conditions (Figure 2d).

**Figure 3:**
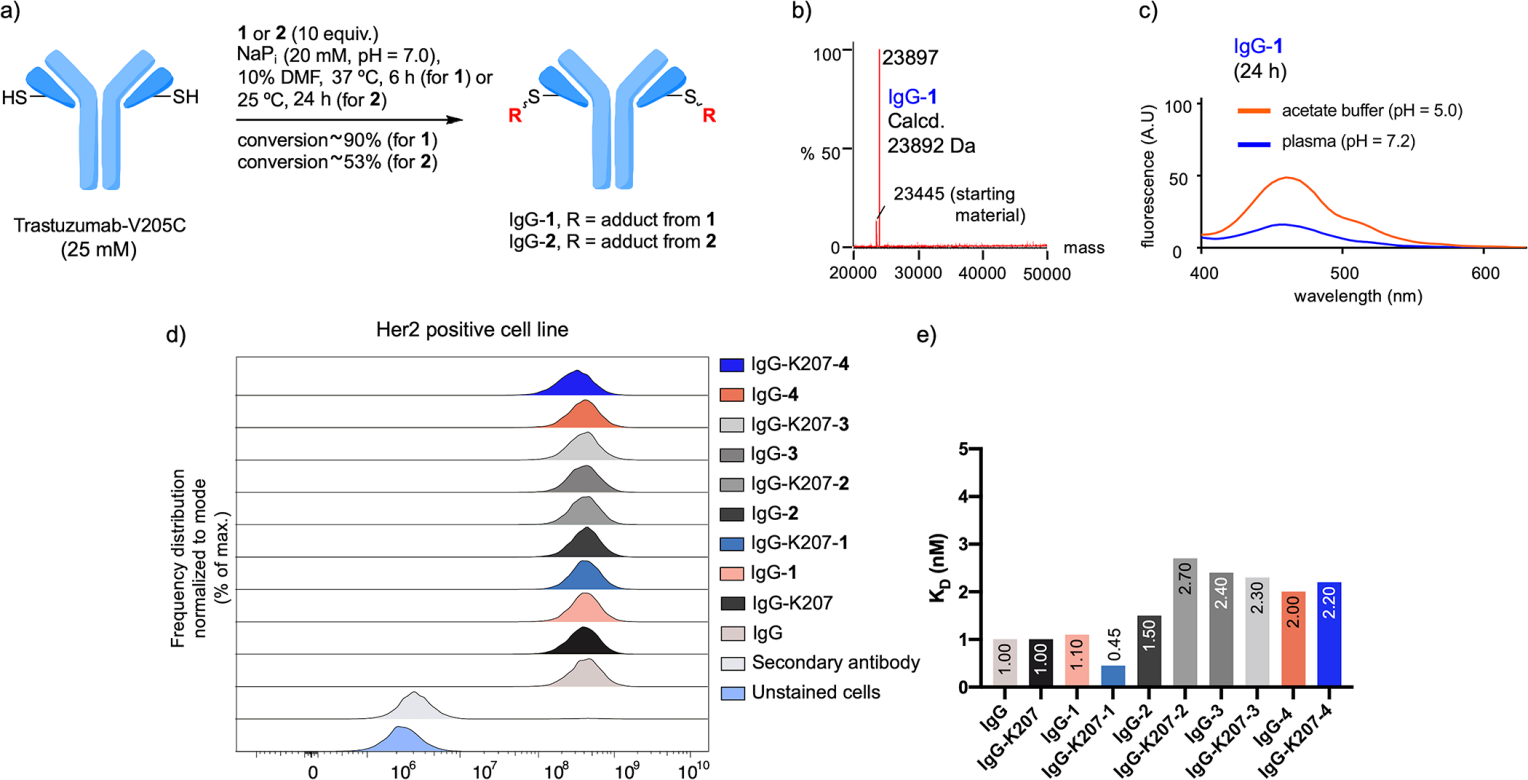
(a) Optimized conditions for the preparation of IgG-**1** and IgG-**2**. (b) ESI–MS spectrum of IgG-**1**. No modification of the heavy chain was observed (see also the Supporting Information). (c) Stability studies of IgG-**1** followed by fluorescence. (d) Flow cytometry plots for the conjugates studied in this work obtained by flow cytometry with HER2-expressing cells (SKBR3 cell line, see also Figures S46 and S47). (e) *K*_D_ constants derived from BLI experiments for the conjugates studied in this work with SKBR3 cell line. These values range from 0.45 to 2.7 nM, indicating that all conjugates have a similar binding (Figure S48).

Next, we envisaged the construction of two ADCs with acetals **1** and **2** and IgG (IgG-**1** and IgG-**2**, respectively, Figures 3a, S34 and S36, Schemes S8 and S10). To this purpose, we treated the antibody IgG with 10 equiv. of **1** in NaP_i_ buffer (20 mM, pH 7.0) with 10% DMF as a co-solvent. The mixture was stirred at 37 °C for 6 h, and the extent of the conjugation was analyzed by LC–MS (Figure 3b). Under these conditions, 90% conversion was achieved for IgG-**1**, with only one modification per light chain, as determined by LC–MS analysis, and without any modification in the heavy chain (Figures 3b and S34, Scheme S8). IgG-**1** was stable under the reaction conditions and in human plasma (pH = 7.2) at 37 °C for 24 h (Figure 3c, 15% hydrolysis), as determined by fluorescence of the free coumarin. It is known that trastuzumab can be internalized into cancer cells (cellular endosomes and lysosomes) via an antibody-mediated HER2 mechanism, although not very efficiently.^47,48^ Moreover, the pH in these organelles was found to be 4–4.5.^49^ Based on these considerations, we decided to perform the stability studies of conjugate IgG-**1** at a more acidic pH, compared to free acetal **5**, for biological relevance. As expected, at pH = 5.0, 50% of the coumarin was released after 24 h (Figure 3c). As a next step, we tested the effect of the modifications on the specificity of the conjugate toward HER2+ cells. IgG-**1** was incubated with SKBR3 cells, expressing the HER2 receptor on their surface and with MDA-MB 231 cell line as a negative control.^50^ As inferred from the flow cytometry studies (Figures 3d and S46), IgG-**1** binds to the surface of SKBR3 cells selectively, whereas no binding was observed for the negative control (MDA-MB 231 cells, Figure S47). Furthermore, a *K*_D_ value of 1.1 ± 0.4 nM was experimentally determined for IgG-**1** binding to SKBR3 by bio-layer interferometry (BLI) assays (Figures 3e and S48). These data indicate that the antibody retains its activity after chemical modification. It is important to note that similar results, in terms of selectivity and affinity toward the two cell lines tested, were also obtained with the other antigens studied in this work (see Figure 3d,e below).

**Figure 4:**
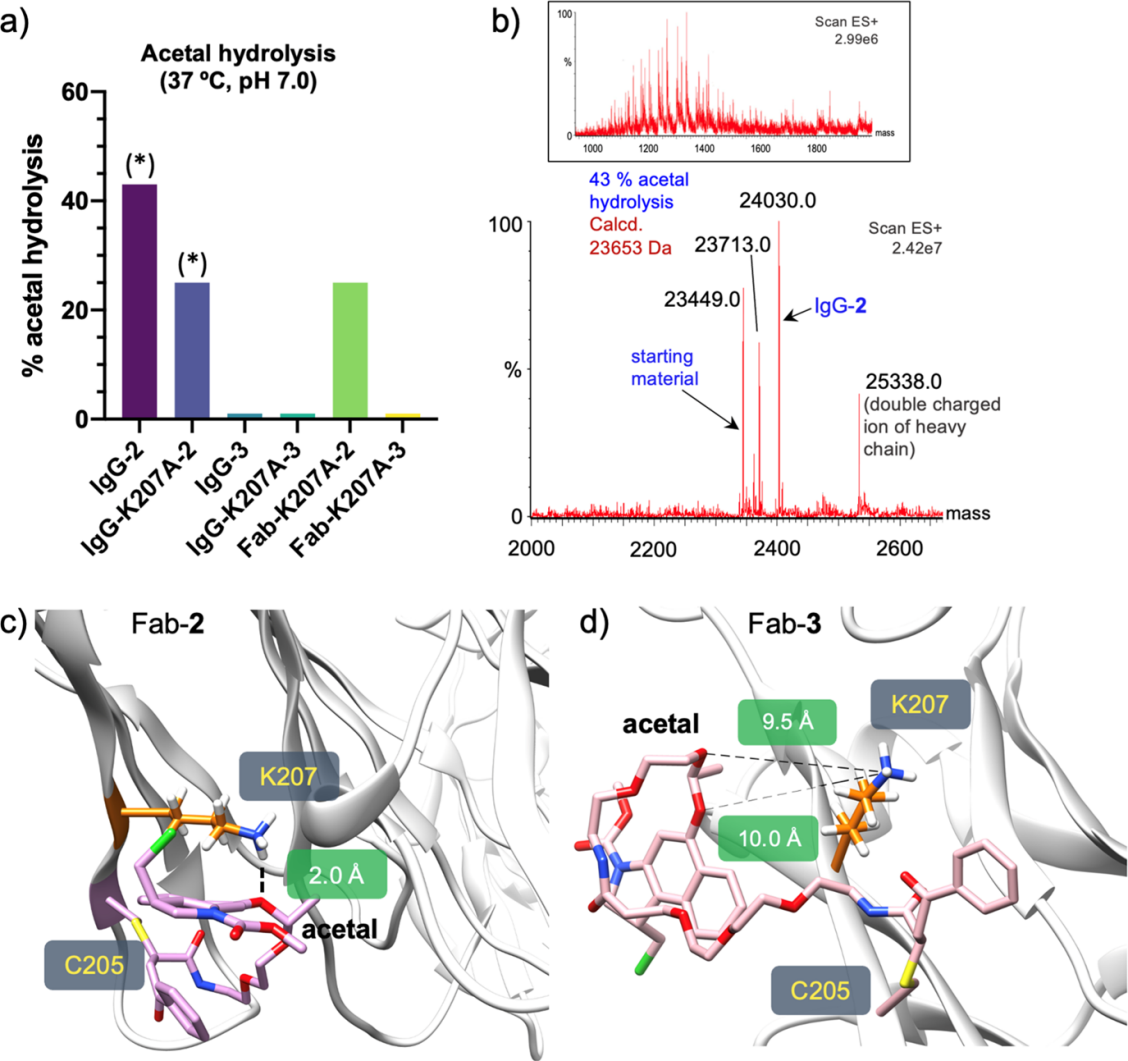
(a) Stability (in the reaction medium) of acetals **2** and **3** conjugated to different antibodies. (*) =25 °C, pH 7, 24 h. (b) Hydrolysis of acetal in IgG-**2**, as determined by the ESI–MS, showing the combined ion series (top) and the deconvoluted mass spectrum (bottom). Conditions: reaction medium (25 °C, pH 7, 24 h). Representative snapshots derived from 0.5 μs MD simulations performed on Fab-**2**. (c) or Fab-**3** (d). The *R* configuration at both stereocenters of the linker was considered in the calculations performed on Fab-**3**.

Next, we studied the stability of IgG-**2**, which was equipped with the linker for the duocarmycin derivative (Figures 3a and S36, Scheme S10). The MS spectra showed that this conjugate was not stable even under the reaction conditions. When the conjugation reaction was carried out at 25 °C and using 10 equiv of **2**, the hydrolysis of the acetal under these milder conditions was ∼45% after 24 h (Figure 4a,b). It is noteworthy that IgG-**2** could not be obtained under the same conditions as for the synthesis of IgG-**1** as it was unstable at 37 °C.

Considering the stability of acetal **5** in plasma (10% hydrolysis after 24 h, Figure 2d), the presence of specific residues near the conjugation site and/or the 3D orientation of linker **2** could have an influence on the hydrolysis rate. To provide further insights at the atomic level, we performed molecular dynamics (MD) simulations on conjugates IgG-**1** and IgG-**2**, using the Fab fragment of the antibody (PDB entry 1N8Z,^51^Figures 4c and S49–S51, derivatives Fab-**1** and Fab-**2**, respectively) and the four possible diastereomers produced upon the conjugation reaction with linkers **1** and **2**. Of note, transient hydrogen bonds are observed between oxygen atoms of the acetal group and the ammonium group of the side chain of Lys-207 in Fab-**2**, accounting for 20% of the total trajectories (Figures 4c and S51). Consequently, this lysine could act as an acidic catalyst to promote the hydrolysis reaction. In contrast, no contacts between the acetal oxygens and the antibody on Fab-**1** were detected in the MD simulations, consistent with the stability of the conjugate explained above (Figure S51). Interestingly, recent studies performed by our group suggest that Lys-207 is involved in the stabilization of dichloro-butenediamide-based linkers.^52^ The logP values were then estimated for acetal **5** and its analogue with coumarin (compound **S3** in the Supporting Information) following the Crippen fragmentation method.^53,54^ These values were calculated to be 2.4 and 1.0, respectively, indicating the higher hydrophobicity of the structure with the duocarmycin derivative. Thus, the nature of **2** could favor the proximity of the linker to the surface of the protein, allowing Lys-207 to protonate the acetal. This hypothesis is consistent with the lower solvent-accessible surface area (SASA) value obtained for the acetal moiety in Fab-**2** (average SASA value = 42.6 ± 23.0 Å^2^) compared with that found in Fab-**1** (average SASA value = 62.6 ± 7.7 Å^2^, Figures S49 and S50).

To gain insights into the mechanism of hydrolysis of acetal **2** when conjugated, the p*K*_a_ of Lys-207 was estimated in unligated Fab and ligated Fab-**2** using constant pH molecular dynamics simulations^55^ (CpHMD, see Supporting Information, Tables S3–S7, Figures S24–S28). For comparison, the p*K*_a_ of the more distant and solvent-exposed Lys-350 was also computed. Based on our calculations, Lys-207 has a lower p*K*_a_ (8.2) than Lys-350 (9.4). Of note, this intrinsically higher acidity of Lys-207 is exacerbated by the presence of the covalently modified Cys-205 (p*K*_a_ 6.9–7.7 for Lys-207 vs p*K*_a_ 9.3–9.5 for Lys 305, depending on the calculated diastereomer) because of the more hydrophobic microenvironment created by the conjugated duocarmycin moiety. Such a low p*K*_a_ value suggests that Lys-207 might be partially deprotonated at physiological pH. Interestingly, the deviation from unity of the Hill coefficients calculated for Lys-207 (*n* = 0.5–0.8) also suggests cooperativity in the calculated dissociation.^56^

**Figure 5:**
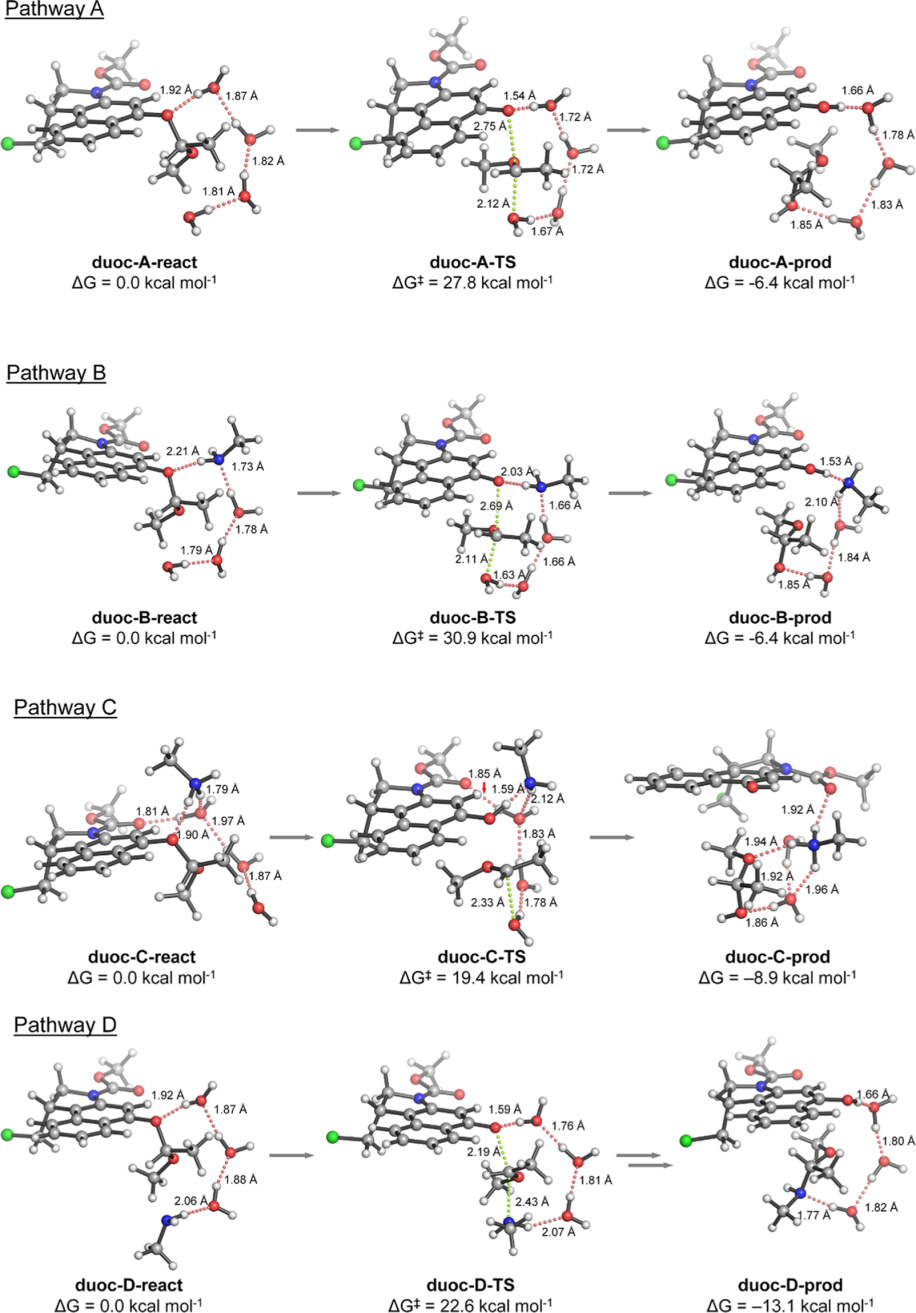
Geometries and relatives stabilities for the reactants, rate-limiting transition states, and final products for the four proposed mechanisms for acetal cleavage calculated with PCM(H_2_O)/M06-2X/6-31+G(d,p): water-assisted hydrolysis (pathway A), neutral amine-assisted hydrolysis (pathway B), charged ammonium-assisted hydrolysis (pathway C), and water-assisted aminolysis (pathway D). Relative free energies (Δ*G* and Δ*G*^⧧^) are given in kcal·mol^–1^ and interatomic distances in angstroms. Hydrogen bond interactions are shown as dotted red lines. Breaking/forming bonds on the transition states are shown as dotted green lines. The whole computed reaction pathways C and D are available in the Supporting Information (Figure S30).

Given these results, we then investigated the ability of Lys-207 to promote the cleavage of the acetal linker by acting as either a proton-transfer group (in its neutral or protonated forms) or a nucleophile (Scheme S7) using quantum mechanics. An initial study performed on a very reduced model of the protonated acetal revealed that hydrolysis using a water molecule as a nucleophile and phenol as a leaving group had an activation barrier around 7 kcal·mol^–1^ lower than that using methanol as a leaving group. Besides, the corresponding hemiacetal product was thermoneutral and around 8 kcal·mol^–1^ more stable. Based on these results, we constructed a larger model using duocarmycin methyl acetal and studied the hydrolysis reaction in the absence and the presence of methylamine or methylammonium as surrogates for deprotonated and protonated Lys-207, respectively (Figures 5 and S30). Three water molecules were also included to facilitate proton transfer from the nucleophile to the leaving group and to stabilize the nucleophilic addition transition structures. We first evaluated the reaction profile of acetal hydrolysis in the absence of amine/ammonium, involving therefore four water molecules (pathway A). One of the water molecules attacks the acetalic carbon with the concomitant breaking of the acetal C–O bond (Δ*G*^⧧^ ≈ 28 kcal·mol^–1^) followed by a barrierless proton transfer from the attacking water to the leaving naphtholate, releasing the pro-duocarmycin derivative. Similarly, replacing one of the water molecules by methylamine was calculated to assist the concerted proton transfer-acetal cleavage (pathway B) although with a higher activation barrier (Δ*G*^⧧^ ≈ 31 kcal·mol^–1^).

In contrast, modeling charged methylammonium to assist proton transfer (pathway C) resulted in a two-step process: first, the naphthyl ether is protonated with simultaneous breaking of the acetal C–O bond (Δ*G*^⧧^ = 18.4 kcal·mol^–1^), leading to a highly unstable oxocarbenium ion (Δ*G* = 18.4 kcal·mol^–1^),^57^ which is subsequently trapped by a nearby water molecule with virtually no activation barrier (Δ*G*^⧧^ = 19.4 kcal·mol^–1^) to give the corresponding hemiacetal after proton rearrangement. Obviously, the difference in the cleavage activation barriers for protonated versus neutral amine reflects their intrinsic ability to transfer a proton to the leaving group (i.e., p*K*_a_). Finally, we investigated the water-assisted nucleophilic attack of the neutral amine to the acetalic carbon (pathway D), which resulted in a lower activation barrier (Δ*G*^⧧^ = 22.6 kcal·mol^–1^) than the uncatalyzed hydrolysis due to the higher nucleophilicity of the amine, leading to a thermoneutral ammonium phenolate intermediate, which undergoes a fast proton transfer (Δ*G*^⧧^ ≈ 4 kcal·mol^–1^) to yield the neutral pro-duocarmycin derivative.

Although pathway C (ammonium-catalyzed hydrolysis) has the lowest activation barrier and thus could be considered the main reaction channel toward the final product, the other calculated pathways should not be discarded. In fact, the theoretical reaction rate constant (*k*_theo_) for pro-duocarmycin release considering all the calculated mechanisms, the propensity of Lys-207 to be in the surroundings of the acetal group, and environmental pH, can be approximately simulated (eq S8 in Supporting Information). According to these simulations, the cleavage reaction is accelerated at acidic pH values. At physiological pH 7.4, *k*_theo_ and the concentrations of the reactive species are extremely sensitive to slight variations on the values of the different equilibrium constants, highlighting the importance of considering all the plausible mechanisms involved in acetal cleavage to obtain a realistic picture of the observed reaction.

Considering that Lys-207 could be responsible for the stability of the linker on IgG-**2**, we envisaged two different strategies to mitigate the hydrolysis reaction of the acetal upon conjugation. First, we mutated this residue to alanine on the Fab or on the complete antibody (Fab-K207A or IgG-K207A, respectively (Figure S58). To our delight, the conjugation reaction of the new mutants with acetal **2** (25 °C, pH 7, 24 h) gave better results than that with the parent IgG. On the one hand, the absence of Lys-207 led to a conversion of the IgG-K207A-**2** conjugate ∼76% after 24 h. Also, the conjugates were more stable as only ∼25% of hydrolysis of the acetal was detected under these conditions (Figures 4a, S37 and S38, Schemes S11 and S12). Second, we synthesized acetal **3** (Figure 1b), which has a longer linker between the acetal group and the carbonyl acrylamide moieties. When this acetal was conjugated to the original IgG antibody to give IgG-**3** (Figure S39, Scheme S13), no hydrolysis was observed in the reaction medium, even at 37 °C for 4 h, Figure 4a). According to MD simulations, the distance between Nε of Lys-207 and the carbon of the acetal group averaged ∼12 Å throughout the simulation, preventing this lysine from protonating the acetal (Figures 4d and S52). Finally, to verify that our approach is consistent, we combined both approaches in the Fab and IgG forms to obtain Fab-K207A-**3** and IgG-K207A-**3**, respectively. As expected, the new conjugates (Schemes S14 and S15, Figures S40 and S41) showed high stability in the reaction medium after 4 h at 37 °C (Figure 4a). Our data indicate that Lys-207, located in the vicinity of the reactive Cys-205, can act as an acid catalyst and promote the hydrolysis of acetal-based linkers.

**Figure 6:**
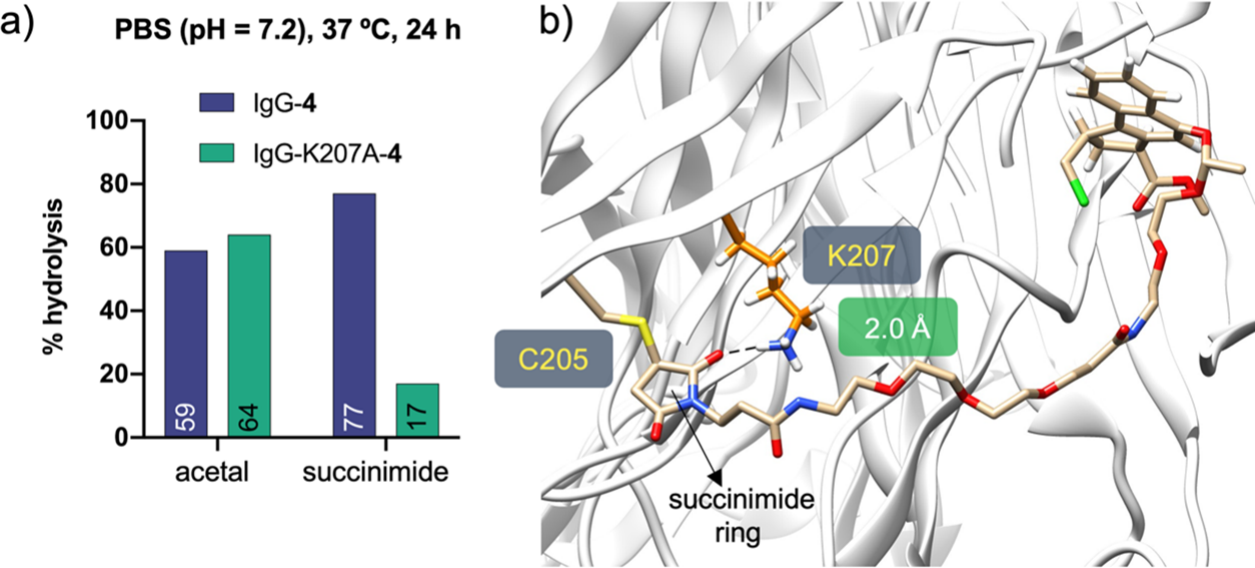
(a) Stability of the acetal group and succinimide ring of IgG-**4** and IgG-K207A-**4** in PBS (pH = 7.2). (b) Representative snapshot derived from 0.5 μs MD simulations performed on Fab-**4**. The *R* configuration at both stereocenters of the linker was considered in the calculations.

It is well-known that maleimide derivatives are routinely used to prepare conjugates of Trastuzumab-V205C, which show specific stability presumably through hydrolysis of the resulting succinimide ring.^10^ To test whether Lys-207 could contribute to this reaction, we prepared linker **4**, a variant of acetal **3**, but with a maleimide scaffold as a Michael acceptor (Figure 1b). We then conjugated derivative **4** to the parent IgG or IgG-K207A antibodies (Schemes S16 and S17, Figures S42 and S43) and tested the stability of the conjugates in PBS buffer (pH = 7.2) at 37 °C for 24 h (Figures 6a and S44 and S45). Interestingly, we detected about 60% more hydrolysis of the succinimide ring when Lys-207 was present in the conjugation site. On the other hand, the hydrolysis of acetal **4** in both conjugates was about 60% under the same conditions. MD simulations performed on Fab-**4** (Figures 6b and S52) show that the carbonyl group of the succinimide ring is located near Lys-207 for about 62% of the total trajectory (distance between the carbonyl group of the succinimide and Nε of Lys-207 < 3.5 Å). Our data indicate that Lys-207 may favor the ring opening reaction and, in this way, contributes to the stabilization of the corresponding conjugate.

We then investigated the effects that other amino acids might have from a theoretical point of view. For this purpose, the Fab-K207X-**2** and Fab-K207X-**4** mutants, where X = Arg, His, Asp, or Glu, were subjected to 0.5 μs MD simulations (see Supporting Information and Figures S53–S57). According to these calculations, Arg-207 (present in the Fab-K207R-**2** mutant) could play a similar role to Lys and act as an acid catalyst since the distance between its guanidino group and the acetal oxygen is ≤4 Å for about 80 ns of the total trajectory time of the MD simulations (Figure S53). For the other mutants studied with acetal **2**, the distance between the corresponding groups of the side chain and the acetal group remains >5 Å throughout the trajectory. Interestingly, in the context of Fab-K207X-**4** variants, the simulations show that mutation of Lys-207 to Arg could also lead to stabilization of the final conjugate by hydrolysis of the succinimide ring. In theory, this observation could also be applied to the K207H mutant.

**Figure 7:**
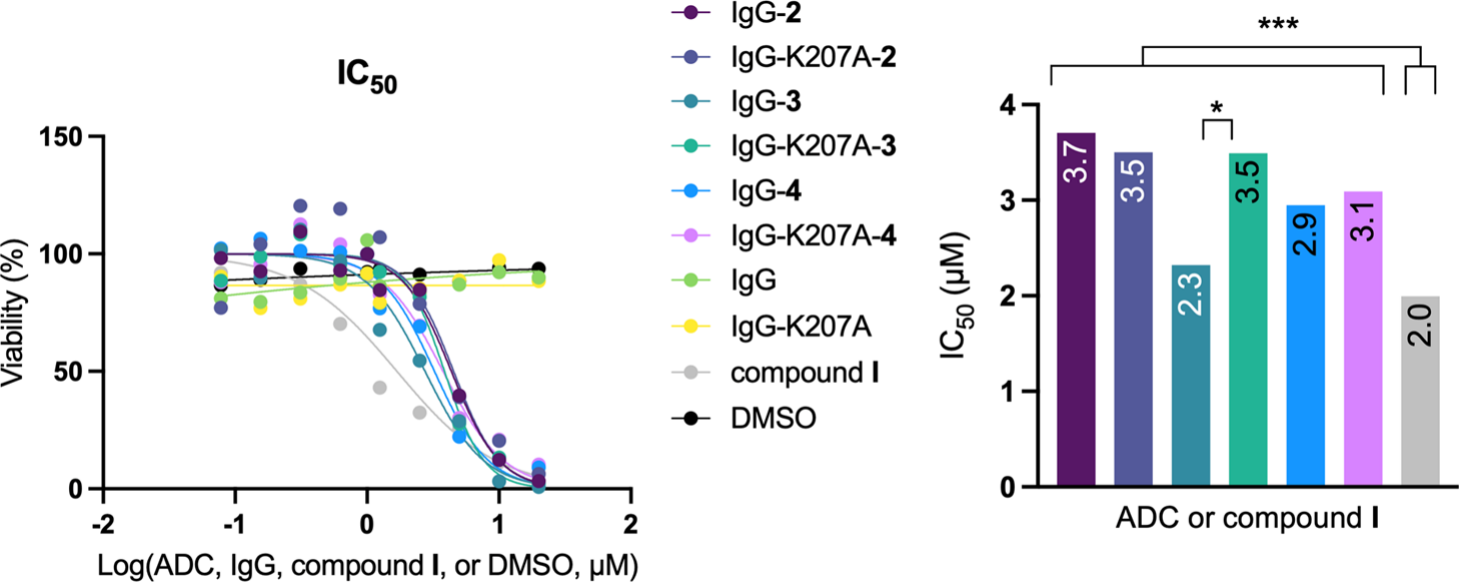
Dose–response curves (left panel) and half maximal inhibitory concentration (IC_50_) values (right panel) of the different conjugates in the SKBR3 cell line. IgG, IgG-K207A, and DMSO were used as negative controls. Statistical analysis: all conjugates and compound **I** against their negative controls; *p* < 0.001. IgG-**3** vs IgG-K207A-**3**; *p* = 0.0173 (*). All conjugates against compound **I**; *p* < 0.001 (***). All other comparisons; *p* > 0.05 (nonsignificant).

Finally, the cytotoxicity of the conjugates prepared in this work bearing the duocarmycin derivative was determined using the Her2 expressing cell line SKBR3. To this purpose, these cells were incubated in the presence of the conjugates (with IgG and IgG-K207A as negative controls) for 48 h. As can be seen in Figure 7, the IC_50_ values ranged from 2.8 to 4.5 μM, indicating that all derivatives are toxic under the experimental conditions (Supporting Information and Figure S59). In general, a significant difference was found between compound **I**, with a slightly lower IC_50_ value of 2.0 μM, and the ADCs. Among these conjugates, the difference was only detected between IgG-**3** and the corresponding mutant IgG-K207-**3** (*p* = 0.0173). These results suggest that the ADCs may be internalized more slowly by SKBR3 cells than compound **I** and that once inside cells, they are hydrolyzed at a similar rate under acidic conditions (e.g., in lysosomes). Although derivative **I** in the SKBR3 cell line has a higher IC_50_ value than that reported for the leukemia cell line L1210,^39,40^ this compound is more toxic to SKBR3 cell line than a variant that lacks the methyl carbamate and contains an additional methyl group (IC_50_ = 4.9 μM).^38^

## Conclusions

In summary, by combining site-directed mutagenesis and MD simulations, we have shown that Lys-207 in Thiomab Tras-LC-V205C is critical for the stability of linkers containing acid-sensitive groups such as acetals. This lysine may act as an acid catalyst and promote hydrolysis of the acetals. Interestingly, when maleimide reagents are used for the conjugation, Lys-207 helps in the hydrolysis of the succinimide ring, resulting from the maleimide, and contributes to the stabilization of the conjugate. Our results support the rational design of ADCs with improved stability and drug–release profiles through removal/addition of specific residues that can promote acid catalysis placed near the conjugation site.

## Supporting Information Available

Synthesis and characterization of acetals **1–4**; cartesian coordinates; electronic energies; Gibbs free energies and lowest frequencies of the DFT-calculated structures; determination of hydrolysis rate constants of several acetals, conjugation reactions, and characterization of ADCs; stability studies in human plasma; cell culture conditions; flow cytometry assays; CpHMD and additional MD simulations; production and characterization of the different mutants of the antibody; affinity studies; cytotoxicity; and IC_50_ calculations (PDF)
