# Supplementary material for: Single Mutation on Trastuzumab Modulates the Stability of Antibody–Drug Conjugates Built Using Acetal-Based Linkers and Thiol-Maleimide Chemistry
Source: J Am Chem Soc. 2022 Mar 16;144(12):5284–94. doi: 10.1021/jacs.1c07675 (PMC8972253; doi:10.1021/jacs.1c07675)
Supplement: Supplementary file 1 — ja1c07675_si_001.pdf [file ja1c07675_si_001.pdf]

A Single Mutation on Trastuzumab Modulates the Stability of Antibody-Drug Conjugates built using Acetal-based Linkers and Thiol-maleimide Chemistry

Xhenti Ferhati,[1,‡] Ester Jiménez-Moreno,[1,‡] Emily A. Hoyt,[2,‡] Giulia Salluce,[2,3] Mar Cabeza-Cabrerizo,[2] Claudio D. Navo,[4] Ismael Compañón,[1] Padma Akkapeddi,[5] Maria J. Matos,[2] Noelia Salaverri,[1] Pablo Garrido,[6] Alfredo Martínez,[6] Víctor Laserna,[7] Thomas V. Murray,[7] Gonzalo Jiménez-Osés,[4,8] Peter Ravn,[7,9] Gonçalo J. L. Bernardes,[2,5,*] and Francisco Corzana[1,*]

[1] Departamento de Química. Centro de Investigación en Síntesis Química. Universidad de La Rioja. 26006 Logroño, Spain.

[2] Department of Chemistry, University of Cambridge, Lensfield Road, CB2 1EW Cambridge, UK.

[3] Departamento de Química Orgánica e Centro Singular de Investigación en Química Biolóxica e Materiais Moleculares (CiQUS), Universidade de Santiago de Compostela, 15782 Santiago de Compostela, Spain

[4] Center for Cooperative Research in Biosciences (CIC bioGUNE), Basque Research and Technology Alliance (BRTA), Bizkaia Technology Park, Building 800, 48160 Derio, Spain.

[5]Instituto de Medicina Molecular João Lobo Antunes, Faculdade de Medicina, Universidade de Lisboa, 1649-028 Lisboa, Av. Prof. Egas Moniz, Portugal.

[6] Angiogenesis Group, Oncology Area, Center for Biomedical Research of La Rioja (CIBIR), 26006 Logroño, Spain.

[7] Biologics Engineering, R&D, Astra Zeneca, Cambridge, UK.

[8] Ikerbasque, Basque Foundation for Science, 48013 Bilbao, Spain

[9] Current address: Biotherapeutic Discovery, H. Lundbeck A/S Ottiliavej 9, 2500 Valby, Denmark.

[‡]These authors contributed equally to the work presented.

Correspondence should be addressed to: francisco.corzana@unirioja.es (F.C.); gb453@cam.ac.uk (G.J.L.B.)

Table of Contents

Reagents and general procedures. 3

Synthesis and characterization of compounds 1-4 and derivative I. 3

Quantum Mechanical calculations 22

Determination of the hydrolysis rate constants 58

Stability of acetal 5 in plasma 60

LC–MS method for analysis of protein conjugation 61

Analysis of protein conjugation by LC–MS 61

Conjugation reactions and characterization of ADCs 62

Stability of ADCs in human plasma and PBS 72

Cell culture conditions 74

Flow cytometry assays 74

Biolayer interferometry 77

Molecular dynamics (MD) simulations 79

Production and characterization of IgG-K207A and Fab-K207A 89

Cytotoxicity and IC_{50} calculation 90

References 92

Reagents and general procedures.

Commercial reagents were used without further purification. Analytical thin layer chromatography (TLC) was performed on Macherey-Nagel precoated aluminium sheets with a 0.20 mm thickness of silica gel 60 with fluorescent indicator UV254. TLC plates were visualized with UV light and by staining with phosphomolybdic acid (PMA) solution (5 g of PMA in 100 mL of absolute ethanol) or sulfuric acid-ethanol solution (1:20). Column chromatography was performed on silica gel (230−400 mesh). ^{1}H and ^{13}C NMR spectra were measured with 500 MHz, 400 MHz or 300 MHz spectrometers with TMS as the internal standard. Multiplicities are quoted as singlet (s), broad singlet (brs), doublet (d), doublet of doublets (dd), triplet (t), or multiplet (m) or combinations thereof. Cq stands for quaternary carbon atom. Spectra were assigned using COSY and HSQC experiments. All NMR chemical shifts (δ) were recorded in ppm and coupling constants (*J*) were reported in Hz. High resolution electrospray mass (ESI) spectra were recorded on a microTOF spectrometer; accurate mass measurements were achieved by using sodium formate as an external reference.

Synthesis and characterization of compounds 1-4 and derivative I.

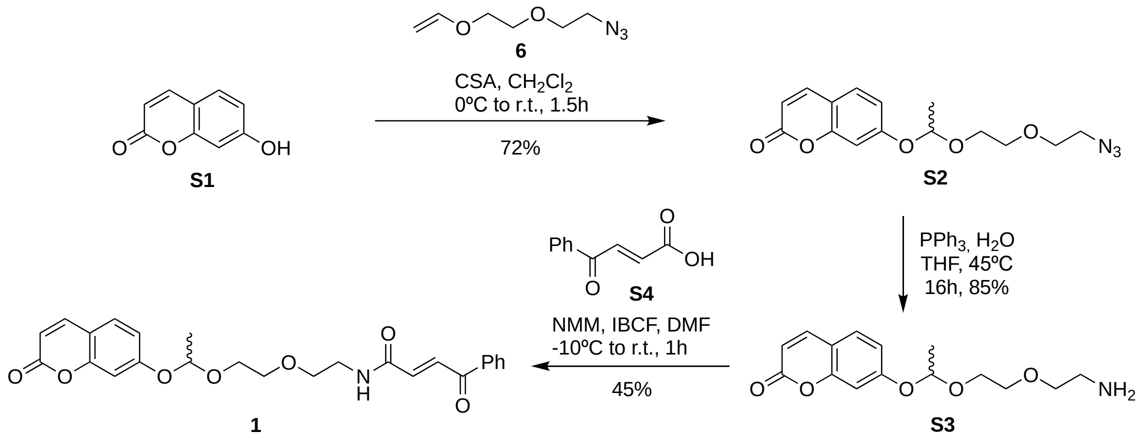

Scheme S1. Synthetic route to prepare compound **1**.

Compound S2. To an ice-cooled solution of **6**[1] (280 mg, 1.78 mmol) in dry CH_2Cl_2 (2 mL) under nitrogen atmosphere, CSA (8 mg, 0.035 mmol) and a solution of compound S1 (96 mg, 0.59 mmol) were added. After 5 minutes, the solution was allowed to warm to r.t. and stirred for 1.5 h. After this time, the reaction mixture was diluted with 40 mL of CH_2Cl_2 and washed with a saturated $NaHCO_3$ solution (20 mL). The product was extracted with CH_2Cl_2 (2 x 40 mL) and the organic phase was dried over anhydrous Na_2SO_4, filtered and

concentrated. The crude was purified by flash column chromatography in CH_2Cl_2:Et_2O (60:1 to 40:1) to give compound **S2** (135 mg, 0.43 mmol, 72%).^{1}H NMR (400 MHz, CD_3OD) δ: 7.86 (d, *J* = 9.5 Hz, 1H, H-4), 7.52 (d, *J* = 8.5 Hz, 1H, H-5), 7.03-6.99 (m, 2H, H-6, H-8), 6.26 (d, *J* = 9.5 Hz, 1H, H-3), 5.64 (q, *J* = 5.3 Hz, 1H, H-acetal), 3.87-3.82 (m, 1H, OCH_2), 3.73-3.62 (m, 5H, OCH_2,) 3.34-3.32 (m, 2H, CH_2-N_3), 1.53 (d, *J* = 5.3 Hz, 3H, CH_3). ^{13}C{^{1}H} NMR (100 MHz, CD_3OD) δ: 161.7 (CO), 160.2 (Cq arom.), 155.4 (Cq arom.), 144.2 (C-4), 129.0 (C-5), 114.4 (C-6), 113.4 (Cq arom.), 112.6 (C-3), 103.8 (C-8), 99.5 (*C*H-acetal), 69.9, 69.7 (2C, O*C*H_2), 64.7 (O*C*H_2), 50.3 (*C*H_2N_3), 18.7 (*C*H_3).

Compound S3. Compound **S2** (111 mg, 0.34 mmol) was dissolved in THF (10 mL) and to this solution PPh_3 (182 mg, 0.69 mmol) and H_2O (49 µL, 2.72 mmol) were added. The solution was heated at 45°C for 16 h and then it was concentrated. The crude obtained was purified by silica gel chromatography in CH_2Cl_2/MeOH/ Et_3N (10:1:0.1) to afford compound **4** (85 mg, 0.29 mmol, 85%). ^{1}H NMR (500 MHz, CD_3OD) δ: 7.91 (d, *J* = 9.4 Hz, 1H, H-4), 7.57 (d, *J* = 8.6 Hz, 1H, H-5), 7.09 (d, *J* = 2.3 Hz, 1H, H-8) 7.03 (dd, *J* = 8.6, 2.3 Hz, 1H, H-6), 6.29 (d, *J* = 9.5 Hz, 1H, H-3), 5.66 (q, *J* = 5.3 Hz, 1H, H-acetal), 3.86-3.84 (m, 1H, OCH_2), 3.74-3.72 (m, 1H, OCH_2), 3.64-3.63 (m, 2H, OCH_2), 3.52 (t, *J* = 5.3 Hz, 2H, OCH_2), 2.78 (t, *J* = 5.3 Hz, 2H, CH_2NH_2), 1.55 (d, *J* = 5.3 Hz, 3H, CH_3). ^{13}C{^{1}H} NMR (125 MHz, CD_3OD) δ: 161.7 (CO), 160.3 (Cq arom.), 155.4 (Cq arom.), 144.2 (C-4), 129.0 (C-5), 114.4 (C-6), 113.4 (Cq arom.), 112.6 (C-3), 103.7 (C-8), 99.5 (*C*-acetal), 71.6, 69.7 (2C, O*C*H_2) 64.6 (O*C*H_2), 40.6 (*C*H_2NH_2), 18.6 (*C*H_3). HRMS (ESI+): m/z calculated for $C_{15}H_{20}NO_5$ $[M+H]^+$ 293.1341, found 294.1331.

Synthesis of compound 1. To a solution of compound **S4** (60 mg, 0.14 mmol) in dry DMF (1 mL) at -10 ºC. was added isobutyl chloroformate (IBCF, 56 µL, 0.43 mmol) and *N*-methylmorpholine (NMM, 47 µL, 0.43 mmol) under stirring. 0.28 mL of this solution were added to a solution of **S3** (16 mg, 0.038 mmol) in dry DMF, cooled at -10 ºC. After 15 min, the reaction was allowed to warm at r.t. and stirred for 1 h. The reaction mixture was then diluted with 20 mL of CH_2Cl_2, washed with H_2O (20 mL) and the organic phase was dried over anhydrous Na_2SO_4, filtered and concentrated. The crude product was purified by column chromatography in CH_2Cl_2/acetone (20:1) to afford compound **1** as a light-yellow solid (36 mg, 0.063 mmol, 45%). ^{1}H NMR (400 MHz, CD_3OD) δ (ppm): 8.04 – 8.02 (m, 2H, H-Ph), 7.88 – 7.84 (m, 2H, H-4, CH=CH), 7.69 – 7.56 (m, 1H, H-Ph), 7.58 – 7.52 (m, 3H, H-5, H-Ph), 7.06 – 6.98 (m ,3H, H-8, H-6, CH=CH), 6.26 (d, *J*=9.5 Hz, 1H, H-3), 5.65 (q, *J*=5.2 Hz, 1H, H-acetal), 3.89 – 3.84 (m, 1H, OCH_2), 3.76 – 3.71 (m, 1H, OCH_2), 3.67 – 3.60 (m,

4H, CH_2-PEG), 3.49 - 3.47 (m, 2H, CH2NH), 1.53 (d, *J*=5.2 Hz, 3H, CH3). $^{13}C\{^1H\}$ NMR (125 MHz, CD_3OD) δ (ppm): 190.0 (CO), 165.3 (CO amide), 161.8 (CO), 160.2 (Cq arom.), 155.4 (Cq arom.), 144.2 (C-4), 136.9 (Cq), 135.1 (CH=CH), 133.5 (CH-Ph), 132.6 (CH=CH), 129.0, 128.6, 128.4 (5C, 4 CH-Ph, C-5), 114.4 (C-6), 113.4 (Cq), 112.6 (C-3), 103.7 (C-8), 99.6 (CH-acetal), 69.8, 68.8 (2C, OCH_2-PEG), 64.8 (OCH_2), 39.4 (CH_2NH), 18.7 (CH_3). HRMS (ESI+): m/z calcd. for $C_{25}H_{26}NO_7$ [M+H]+ 452.1709, found 452.1711.

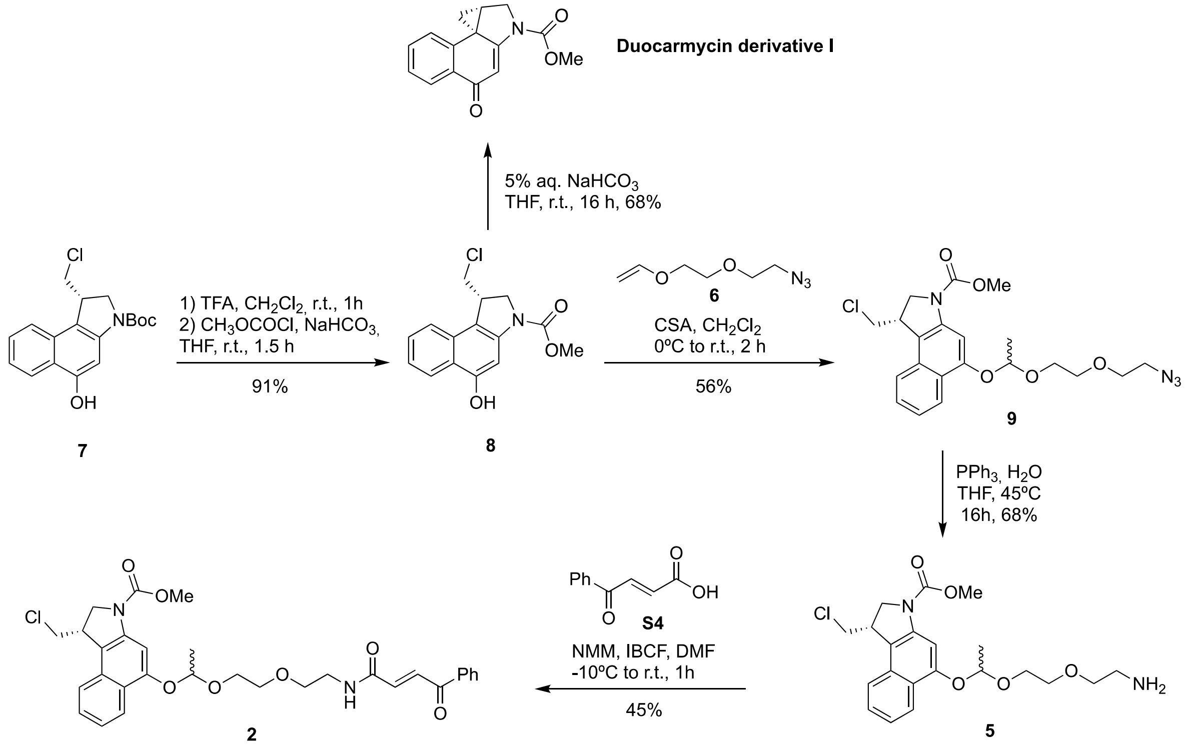

Scheme S2. Synthetic route to prepare compound **2** and duocarmycin derivative **I**.

Compound 8. To a solution of commercially available compound **7** (80 mg, 0.24 mmol) in CH_2Cl_2 (3 mL), TFA (460 µL) was added, and the reaction was stirred at 25 °C. After 1 h, the mixture was concentrated, and the crude product was dissolved in THF (4.2 mL), cooled at 0 °C. Next, $NaHCO_3$ (60 mg) and CH_3OCOCl (37 µL, 0.48 mmol) were added. After 1.5 h, the reaction was diluted with of CH_2Cl_2 (40 mL) and the organic phase washed with H_2O (20 mL), dried over anhydrous Na_2SO_4, filtered and concentrated. The crude was purified by silica gel column chromatography in hexane/AcOEt (1:1) to give compound **8** (64 mg, 0.22 mmol, 91%) as a grey solid. ^1H NMR (400 MHz, $CDCl_3$) δ: 8.58 (brs, 1H, OH), 8.27 (d, *J* = 8.4 Hz, 1H, H arom.), 7.96 (brs, 1H, H arom.), 7.64 (d, *J* = 8.3 Hz, 1H, H arom.), 7.54-7.49 (m, 1H, H arom.), 7.39-7.35 (m, 1H, H arom.), 4.35-4.32 (m, 1H, CH_2N), 4.16-4.11 (m, 1H, CH_2N), 4.01-3.92 (m, 5H, OCH_3, CH_2Cl, CH), 3.42 (d, *J* = 10.6 Hz, 1H, CH_2Cl). $^{13}C\{^1H\}$

NMR (75 MHz, $CDCl_3$) δ: 154.8 (N*C*O), 154.7 (Cq arom.) 140.6 (Cq arom.), 130.3 (Cq arom.), 127.7 (*C*H arom.), 123.8 (*C*H arom.), 123.1 (*C*H arom.), 122.0 (Cq arom.), 121.9 (*C*H arom.), 114.2 (Cq arom.), 98.8 (*C*H arom.), 53.4 (O*C*H_3), 52.8 (*C*H_2N), 46.5 (*C*H_2Cl), 42.0 (*C*H). HRMS (ESI+): m/z calculated for $C_{15}H_{14}NClO_3Na$ $[M+Na]^+$ 314.0554, found 314.0550.

Duocarmycin derivative I. To a solution of compound **8** (8 mg, 0.027 mmol) in THF (1 mL) and a 5% aqueous solution of $NaHCO_3$ was added. After stirring overnight at room temperature, the mixture was diluted with CH_2Cl_2 (10 mL) and washed with H_2O (5 mL). The organic phase was dried over anhydrous Na_2SO_4, filtered, concentrated and the resulting crude product was purified by column chromatography in AcOEt/hexane (1:1) to afford duocarmycin derivative **I** (6 mg, 87%). ^{1}H NMR (300 MHz, CD_3OD) δ: 8.12 (dd, *J*=7.9, 1.4 Hz, 1H, arom.), 7.61 – 7.56 (m , 1H, arom.), 7.46 – 7.40 (m, 1H, arom.), 7.13 – 7.10 (m, 1H, arom.), 6.92 (s, 1H, arom.), 4.14 – 4.05 (m, 2H, CH_2N), 3.87 (s, 3H, OCH_3), 3.13 – 3.07 (m, CH) 1.76 (dd, *J*=7.8, 4.2 Hz, 1H, CH_2), 1.55 (‘t’, *J*=4.7 Hz, 1H, CH_2). $^{13}C\{^1H\}$ NMR (75 MHz, CD_3OD) δ: 187.1 (CO), 162.4 (Cq), 153.2 (Cq), 140.9 (Cq), 132.1 (CH arom.), 131.9 (Cq), 126.1 (CH arom.), 125.9 (CH arom.), 121.6 (CH arom.), 107.2 (CH arom.), 52.9 (CH_2N), 52.6 (OCH_3), 33.4 (Cq), 29.0 (CH_2), 24.0 (CH) HRMS (ESI+): m/z calculated for $C_{15}H_{13}NO_3Na$ $[M+Na]^+$ 278.0788, found 278.0784.

Compound 9. To a solution of **8** (24 mg, 0.08 mmol) in dry CH_2Cl_2 (600 µL) at 0 °C was added compound 6 (56 mg, 0.36 mmol), followed by a few drops of dry THF to complete solubilisation. After that, a 5 mg/mL solution of CSA (320 µL, 0.0072 mmol) in CH_2Cl_2 was added and the reaction was allowed to warm to r.t. After completion of the reaction, the mixture was diluted with CH_2Cl_2 (40 mL) and washed with H_2O (20 mL), dried over anhydrous Na_2SO_4, filtered and concentrated. The crude mixture was purified by a silica gel column chromatography in CH_2Cl_2/Et_2O (70:1) to afford compound **9** (20 mg, 0.045 mmol, 56%) as a diastereomeric mixture. ^{1}H NMR (400 MHz, CD_3OD) δ: 8.09 (d, *J* = 8.4 Hz, 1H, H arom.), 7.75 (brs, 1H, H arom.), 7.63-7.60 (m, 1H, H arom.), 7.42-7.38 (m, 1H, H arom.), 7.26-7.22 (m, 1H, H arom.), 5.61 (q, *J* = 5.2 Hz, 1H, H-acetal), 4.17-4.13 (m, 1H, C*H*$_2$N), 4.06-4.00 (m, 1H, C*H*$_2$N), 3.97-3.91 (m, 1H, C*H*), 3.84-3.81 (m, 1H, C*H*$_2$Cl), 3.80-3.73 (m, 4H, OC*H*$_3$, C*H*H-PEG), 3.64-3.58 (m, 1H, C*H*H-PEG), 3.57-3.47 (m, 5H, C*H*$_2$-PEG, C*H*$_2$Cl), 3.19-3.15 (m, 2H, C*H*$_2$N$_3$), 1.52-1.50 (2 x d, *J* = 5.2 Hz, 3H, C*H*$_3$ 2 diastereomers). $^{13}C\{^1H\}$ NMR (75 MHz, CD_3OD) δ: 154.0 (N*C*O), 153.9 (Cq arom.) 140.8 (Cq arom.), 130.3 (Cq arom.), 127.3 (*C*H arom.), 123.2 (*C*H arom.), 123.1 (Cq arom.), 122.9 (*C*H arom.), 121.9 (*C*H arom.), 115.9 (Cq arom.), 100.4 (C-acetal), 99.4 (*C*H arom.), 70.0, 69.7 (*C*H_2-PEG),

65.5 (*C*H_2-PEG, 2 diastereomers), 52.4 (*C*H_2N), 51.9 (O*C*H_3), 50.3 (*C*H_2N_3), 46.3 (*C*H_2Cl), 41.2 (*C*H), 19.2, 19.1 (*C*H_3, 2 diastereomers). HRMS (ESI+): m/z calculated for $C_{21}H_{25}ClN_4O_5Na$ $[M+Na]^+$ 471.1406, found 471.1406.

Compound 5. To a solution of compound **9** (15 mg, 0.033 mmol) in THF (2 mL) at r.t., PPh_3 (17 mg, 0.067 mmol) was added. After stirring for 20 minutes, H_2O (4.7 μL, 0.26 mmol) was added, and the mixture was stirred for 16 h. The reaction was then concentrated at reduced pressure and the crude was purified by a silica gel column chromatography in CH_2Cl_2/MeOH (7:1) to give compound **5** (9 mg, 0.021 mmol, 68%). ^{1}H NMR (400 MHz, CD_3OD) δ: 8.19 (d, *J* = 8.5 Hz, 1H, H arom.), 7.87 (brs, 1H, H arom.), 7.74 (d, *J* = 8.4 Hz, 1H, H arom.), 7.53-7.49 (m, 1H, H arom.), 7.36-7.32 (m, 1H, H arom.), 5.71 (q, *J* = 5.2 Hz, 1H, H-acetal), 4.29-4.28 (m, 1H, C*H_2*N), 4.20-4.14 (m, 1H, C*H_2*N), 4.11-4.09 (m, 1H, C*H*), 3.96-3.95 (m, 1 H, C*H_2*Cl), 3.93-3.85 (m, 4H, OC*H_3*, C*H*H-PEG), 3.76-3.72 (m, 1H, CH*H*-PEG), 3.65-3.59 (m, 3H, C*H_2-PEG*, CH_2Cl), 3.50-3.47 (m, 2H, C*H_2*-PEG), 2.77-2.74 (m, 2H, C*H_2*NH_2), 1.61 (d, *J* = 5.2 Hz, 3H, C*H_3*). ^{13}C{^{1}H} NMR (75 MHz, CD_3OD) δ: 153.9 (2C, N*C*O, Cq arom.), 140.9 (Cq arom.), 130.3 (Cq arom.), 127.4 (*C*H arom.), 123.2, 123.1 (*C*H arom., Cq arom.), 122.9 (*C*H arom.), 121.9 (*C*H arom.), 116.1 (Cq arom.), 100.4 (C-acetal), 99.5, 99.3 (*C*H arom., 2 diastereomers), 69.9 (2C, *C*H_2-PEG), 65.4 (*C*H_2-PEG), 52.4 (*C*H_2N), 51.9 (O*C*H_3), 46.4, 46.3 (*C*H_2Cl, 2 diastereomers), 41.1 (*C*H), 40.1 (*C*H_2NH_2), 19.1 (*C*H_3, 2 diastereomers). HRMS (ESI+): m/z calculated for $C_{21}H_{28}ClN_2O_5$ $[M+H]^+$ 423.1681, found 423.1683.

Compound 2. To a solution of **S4** (60 mg, 0.14 mmol) in dry DMF (1 mL) at -10 ºC was added isobutyl chloroformate (IBCF, 56 μL, 0.43 mmol) and *N*-methylmorpholine (NMM, 47 μL, 0.43 mmol) under stirring. 0.28 mL of this solution were added to a solution of compound 5 (16 mg, 0.038 mmol) in dry DMF, cooled at -10 ºC. After 15 min, the reaction was allowed to warm at r.t. and stirred for 1 h. The crude reaction mixture was diluted with 20 mL of CH_2Cl_2 and washed with H_2O (20 mL). The organic phase was dried over anhydrous Na_2SO_4, filtered and concentrated. The crude was purified by column chromatography in CH_2Cl_2/acetone (20:1) to give compound **2** as a light-yellow solid (36 mg, 0.063 mmol, 45%). ^{1}H NMR (400 MHz, CD_3OD) δ: 8.20 (d, *J* = 8.5 Hz, 1H, H arom.), 8.02-7.99 (m, 2H, H arom.), 7.87-7.81 (m, 2H, H arom., C*H*=CH), 7.72 ('t', *J* = 7.9 Hz, 1H, H arom.), 7.67-7.64 (m, 1H, H arom.), 7.56-7.49 (m, 3H, H arom.), 7.37-7.33 (m, 1H, H arom.), 7.03-6.98 (m, 1H, CH=C*H*), 5.75-5.72 (m, 1H, H-acetal), 4.29-4.26 (m, 1H, C*H_2*N), 4.20-4.17 (m, 1H, C*H_2*N), 4.07-3.98 (m, 1H, C*H*), 3.96-3.90 (m, 5H, OC*H_3*, C*H*HCl, C*H*H-PEG), 3.89-3.80 (m, 1H, CH*H*-PEG), 3.78-3.67 (m, 5H, C*H*H-PEG, CH*H*Cl), 3.63-3.60 (m, 2H, C*H_2*NHCO), 1.63 (d, *J* = 5.2 Hz, 3H,

CH_3). $^{13}C\{^1H\}$ NMR (100 MHz, CD_3OD) δ: 190.0 (CO), 165.3 (CO amide), 153.9 (2C, NCO, Cq arom.), 136.8 (Cq arom.), 135.1 (*C*H=CH, 2 diastereomers), 133.5 (*C*H arom.), 132.6, 132.5 (*C*H arom., 2 diastereomers), 130.3 (Cq arom.), 128.6, 128.4 (4 *C*H arom.), 127.4 (*C*H arom.), 123.2, 123.1 (*C*H arom. 2 diastereomers), 122.9 (*C*H arom.), 121.9 (*C*H arom.), 116.03 (Cq arom.), 100.5, 100.4 (C-acetal, 2 diastereomer), 99.5, 99.4 (*C*H arom., 2 diastereomers), 69.9, 69.8, 68.9, 68.8 (2 CH_2, 2 diastereomers), 65.6, 65.4 (OCH_2, 2 diastereomers), 52.4 (CH_2N), 51.9 (OCH_3), 46.3 (CH_2Cl), 41.2 (*C*H), 39.4 (CH_2-NHCO), 19.2, 19.1 (CH_3, 2 diastereomers). HRMS (ESI+): m/z calculated for $C_{31}H_{33}ClN_2O_7Na$ $[M+Na]^+$ 603.1869, found 603.1870.

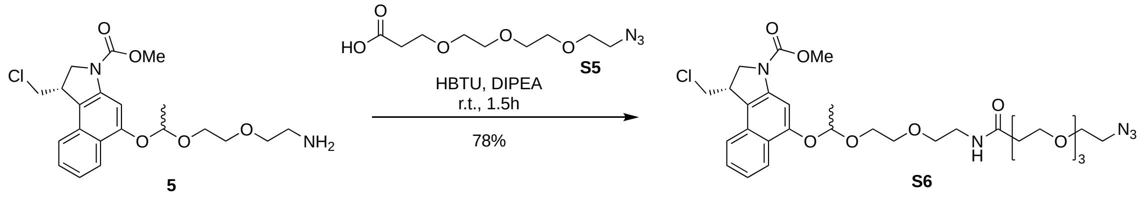

Scheme S3. Synthetic route to compounds **S6**.

Compound S6. To a solution of compound **S5** (30 mg, 0.121 mmol) in DMF (0.4 mL) was added HBTU (45 mg, 0.113 mmol) and DIPEA (43 mL, 0.242 mmol) and it was stirred for 5 minutes. This solution was then added to a solution of compound 5 (34 mg, 0.081 mmol) in DMF (0.4 mL). After 1.5 h the solvent was removed under reduced pressure and the residue was purified by flash chromatography (CH_2Cl_2/MeOH gradient 1:0 to 60:1) to afford compound **S6** (41 mg, 0.063 mmol, 78%). ^1H NMR (300 MHz, CD_3OD) δ: 8.22 (d, *J* = 8.5 Hz, 1H, arom.), 7.87 (bs, 1H, arom.), 7.76 (ddt, *J* = 8.3, 1.9, 1.0 Hz, 1H, arom.), 7.54 (ddd, *J* = 8.3, 6.8, 1.3 Hz, 1H, arom.), 7.38 (ddd, *J* = 8.1, 6.8, 1.2 Hz, 1H, arom.), 5.73 (qd, *J* = 5.1, 1.0 Hz, 1H, H-acetal), 4.29 (dt, *J* = 11.2, 2.4 Hz, 1H), 4.24-4.13 (m, 1H), 4.13-4.04 (m, 1H), 4.00-3.94 (m, 1H), 3.94-3.86 (m, 4H, NCOOCH_3), 3.79-3.49 (m, 19H), 3.39-3.29 (m, 4H), 2.42 (td, *J* = 6.2, 3.9 Hz, 2H, NHCOCH_2), 1.64 (d, *J* = 5.2 Hz, 3H, CH_3). $^{13}C\{^1H\}$ NMR (75 MHz, CD_3OD) δ: 174.0 (CO), 155.3 (2C, CO), 131.7, 128.8, 124.6, 124.5, 124.5, 124.3, 123.3, 117.4, 101.9 (C-acetal, diastereomer), 101.8 (C-acetal, diastereomer), 100.7, 71.6, 71.5, 71.3, 71.2, 71.1, 70.5 (2C), 68.2, 67.0, 66.9, 53.8, 53.5, 51.7, 47.8, 47.7, 42.6, 40.4, 37.6, 20.6 (2C, CH_3, diastereomers). HRMS (ESI+): m/z calculated for $C_{30}H_{42}ClN_5O_9Na$ $[M+Na]^+$ 674.2563, found 674.2558.

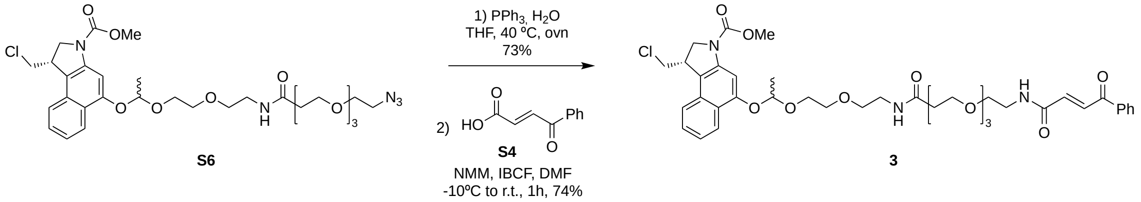

Scheme S4. Synthetic route to compound **3**.

Compound 3. To a solution of compound **S6** (23 mg, 0.035 mmol) in THF (2 mL) was added PPh_3 (18 mg, 0.070 mmol) and H_2O (5 µL, 0.28 mmol) and the mixture was stirred at 40 ºC overnight. The reaction was then concentrated at reduced pressure and the crude was purified by flash chromatography (CH_2Cl_2/MeOH/NH_3 gradient 9:1:0 to 9:1:0.01). To a solution of the compound obtained (16 mg, 0.026 mmol) in anhydrous DMF (0.6 mL) under inert atmosphere at -10 ºC was added 0.2 mL of a solution of compound S4 previously activated. To this end, to a solution of compound S4 (56 mg, 0.318 mmol mmol) in dry DMF (1 mL) at -10 ºC was added isobutyl chloroformate (IBCF, 50 µL, 0.387 mmol) and *N*-methylmorpholine (NMM, 42 µL, 0.382 mmol) under stirring. 15 min after the addition, the reaction was allowed to warm at r.t. and stirred for 1 h. The reaction mixture was diluted with CH_2Cl_2, washed with H_2O and the organic phase dried over anhydrous Na_2SO_4, filtered and concentrated. The crude was purified by flash chromatography (CH_2Cl_2/MeOH gradient 0 to 3%) to afford compound **3** (15 mg, 0.019 mmol, 74%). ^{1}H NMR (400 MHz, CD_3OD) δ: 8.18 (d, *J* = 8.5 Hz, 1H, H arom.), 7.99 (d, *J* = 7.4 Hz, 2H, H arom.), 7.89-7.79 (m, 2H, H arom., C*H*=CH), 7.71 (d, *J* = 8.4 Hz, 1H, H arom.), 7.63 (t, *J* = 7.5 Hz, 1H, H arom.), 7.55-7.45 (m, 3H, H arom.), 7.34 (t, *J* = 7.7 Hz, 1H, H arom.), 7.02 (dd, *J* = 15.3, 2.7 Hz, 1H, CH=C*H*), 5.68 (q, *J* = 5.1, Hz, 1H, H-acetal), 4.24 (dt, *J* = 11.4, 2.5 Hz, 1H), 4.18-4.09 (m, 1H), 4.09-4.00 (m, 1H, C*H*), 3.93 (dd, *J* = 11.2, 3.2 Hz, 1H), 3.89-3.81 (m, 4H, NCOOC*H₃*), 3.76-3.62 (m, 4H), 3.64-3.53 (m, 12H), 3.52-3.45 (m, 4H), 3.31-3.26 (m, 2H), 2.42-2.37 (m, 2H, NHCOC*H₂*), 1.60 (d, *J* = 5.1 Hz, 3H, C*H₃*). ^{13}C{^{1}H} NMR (101 MHz, CD_3OD) δ: 191.3 (CO), 173.9 (CO), 166.6 (CO), 155.3 (CO), 138.2, 136.6 (*C*H=CH), 134.8, 133.9 (CH=*C*H), 131.7, 130.0, 129.8, 128.8, 124.6, 124.5, 124.3, 123.3, 101.9 (C-acetal, diastereomer), 101.8 (C-acetal, diastereomer), 100.7, 71.6, 71.4, 71.3, 71.2, 70.5, 70.2, 68.2, 67.0, 53.8, 53.4, 47.8, 40.9, 40.4, 37.6, 20.6 (2 *C*H₃, diastereomers). HRMS (ESI+): m/z calculated for $C_{40}H_{51}ClN_3O_{11}Na$ $[M+H]^+$ 784.3207, found 784.3197.

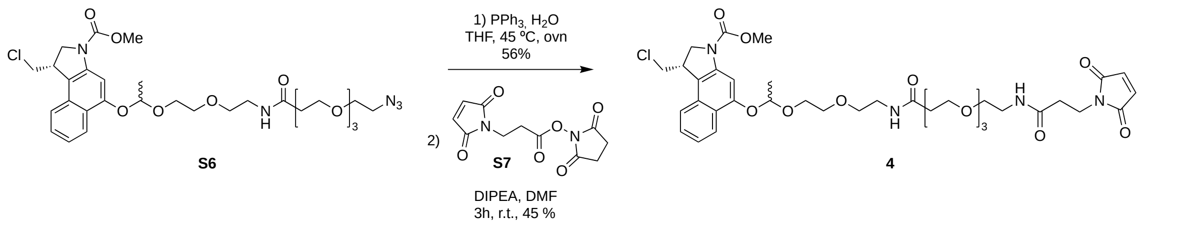

Scheme S5. Synthetic route to compound **4**.

Compound 4. To a solution of compound **S6** (18 mg, 0.028 mmol) in THF (2 mL) was added PPh_3 (15 mg, 0.69 mmol) and H_2O (4 µL, 0.221 mmol). The solution was heated at 45°C overnight and then it was concentrated. The crude obtained was purified by silica gel chromatography in CH_2Cl_2/MeOH/Et_3N (9:1:0.1). To a solution of the compound obtained (9.8 mg, 0,016 mmol) in anhydrous DMF (1 mL) under inert atmosphere was added compound S7 (6.3 mg, 0.024 mmol) and DIPEA (5.5 mL, 0.031 mmol). The reaction was stirred at r.t. for 3 h and then diluted with CH_2Cl_2. The organic phase was washed with water, dried with anhydrous Na_2SO_4, the solvent removed under reduced pressure and the residue was purified by flash chromatography (CH_2Cl_2/MeOH 0% to 2.5%) to afford compound **4** (5.6 mg, 0.007 mmol, 45%). ^{1}H NMR (400 MHz, CD_3OD) δ: 8.20 (d, *J* = 8.5 Hz, 1H, H arom.), 7.86 (bs, 1H, H arom.), 7.76 (d, *J* = 8.4 Hz, 1H, H arom.), 7.53 (t, *J* = 7.6 Hz, 1H, H arom.), 7.36 (t, *J* = 7.7 Hz, 1H, H arom.), 6.82-6.75 (m, 2H, C*H*=C*H*), 5.72 (q, *J* = 5.2 Hz, 1H, H-acetal), 4.29 (dd, *J* = 11.5, 2.3 Hz, 1H), 4.20 (td, *J* = 11.5, 10.2, 4.1 Hz, 1H), 4.15-4.07 (m, 1H, C*H*), 3.96 (dd, *J* = 11.2, 3.1 Hz, 1H), 3.93-3.84 (m, 4H, NCOOCH_3,), 3.78-3.61 (m, 8H), 3.60-3.48 (m, 12H), 3.46 (td, *J* = 5.4, 2.5 Hz, 2H), 3.33-3.25 (m, 4H), 2.48-2.36 (m, 4H, NHCOCH_2), 1.63 (d, *J* = 5.2 Hz, 3H, CH_3). ^{13}C{^{1}H} NMR (101 MHz, CD_3OD) δ: 173.7 (CO), 173.0 (CO), 172.1 (CO), 155.4 (CO), 135.44 (*C*H=*C*H), 131.8, 128.8, 124.7, 124.5, 124.3, 123.3, 102.0 (C-acetal, diastereomer), 101.9 (C-acetal, diastereomer), 71.5, 71.4, 71.3, 71.2, 70.5 (2C), 70.4, 68.2, 66.9, 60.9, 53.9, 53.4, 47.8, 47.7, 42.5, 40.4, 37.6, 35.7, 35.5, 20.6 (2 *C*H_3, diastereomers). HRMS (ESI+): m/z calculated for $C_{37}H_{49}ClN_4O_{12}Na$ $[M+Na]^+$ 799.2928, found 799.2929.

Figure S1. ^{1}H NMR (400 MHz, CD_3OD) of compound **S2**

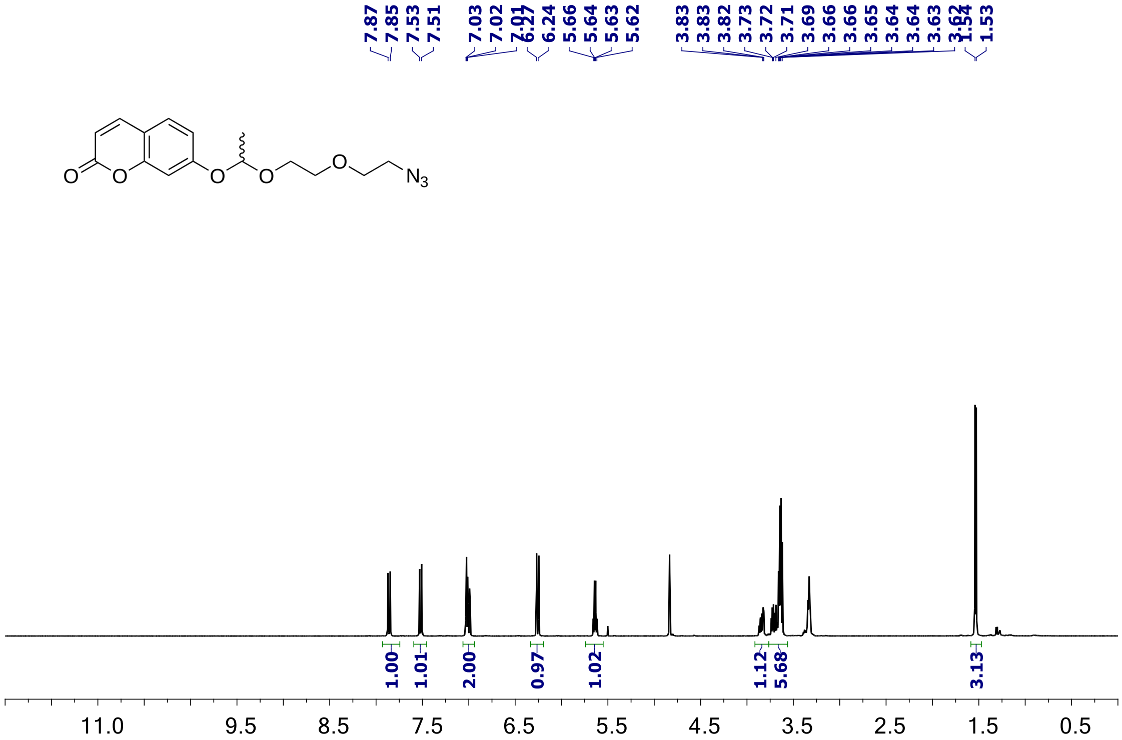

Figure S2. $^{13}C\{^{1}H\}$ NMR (100 MHz, CD_3OD) of compound **S2**

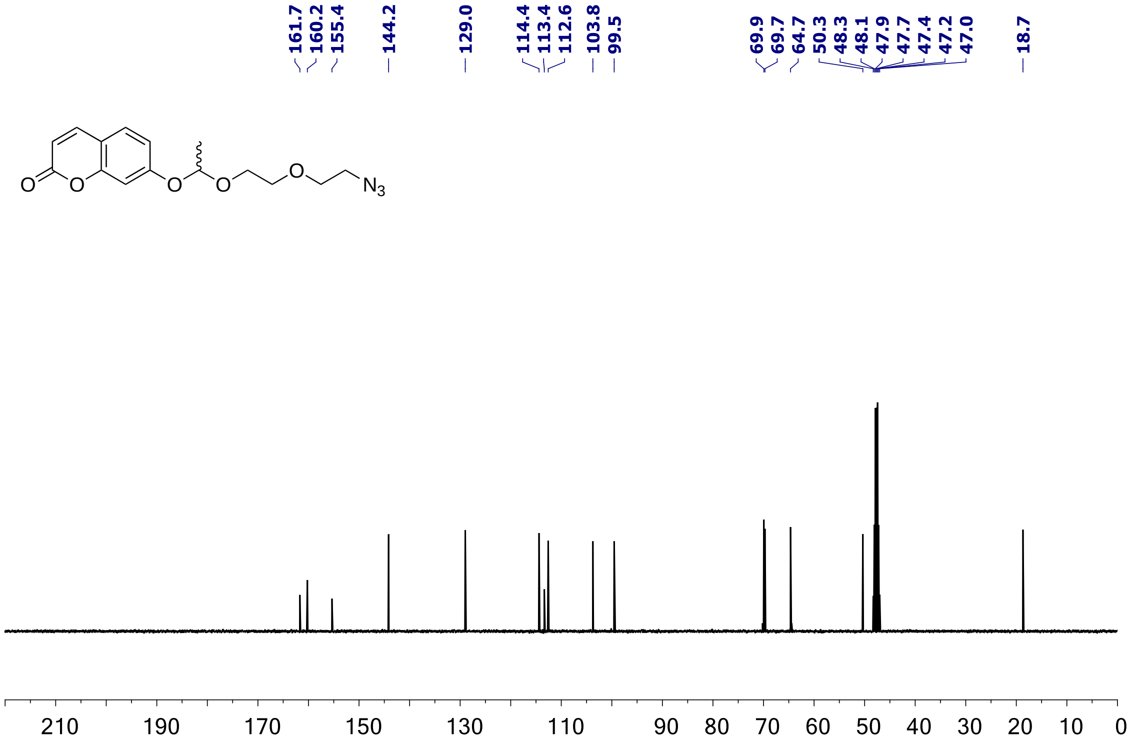

Figure S3. ^{1}H NMR (500 MHz, CD_3OD) of compound **S3**

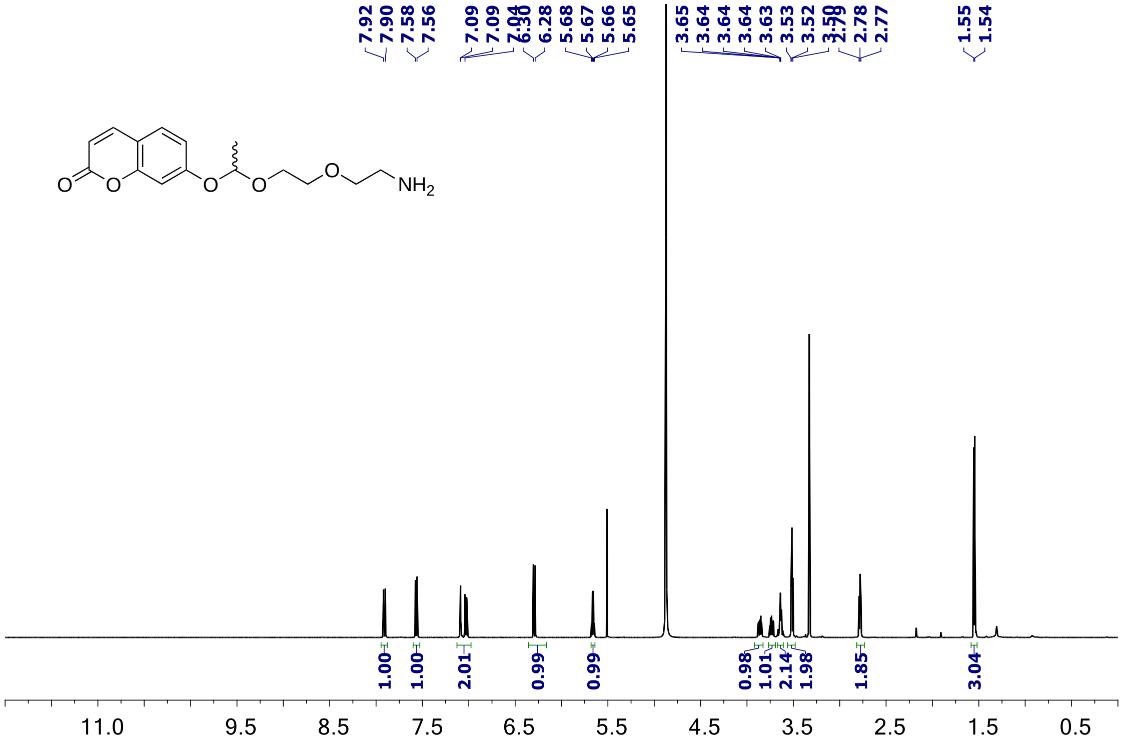

Figure S4. ^{13}C{^{1}H} NMR (125 MHz, CD_3OD) of compound **S3**

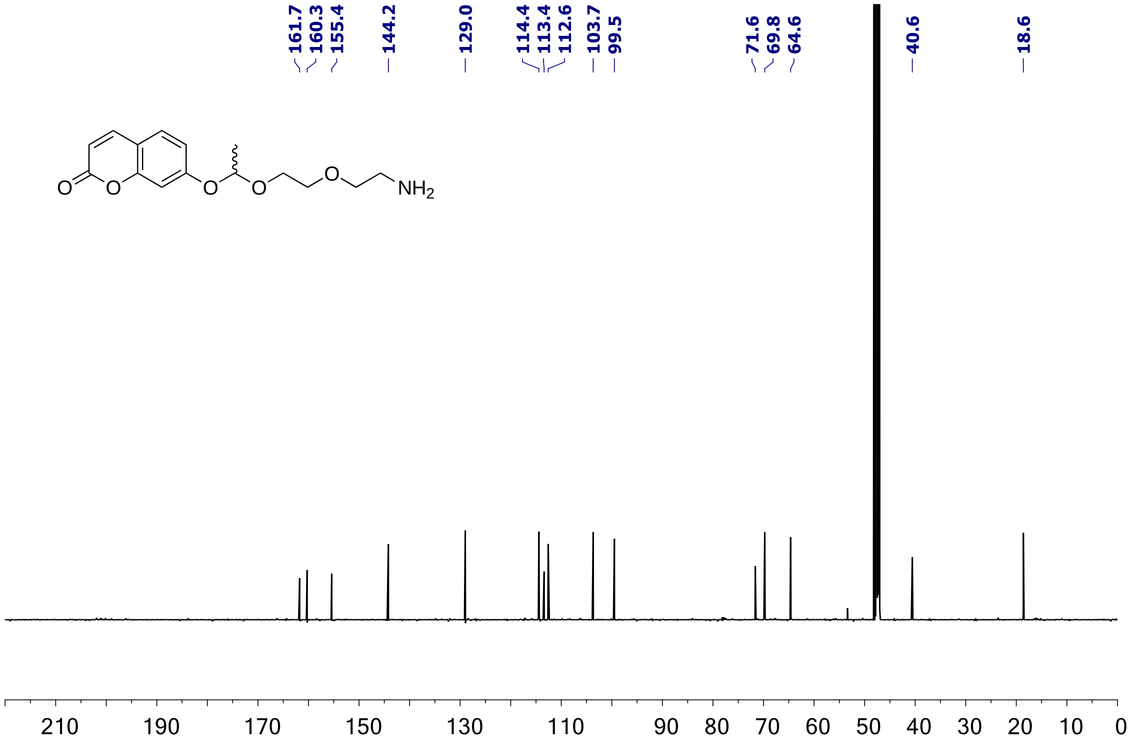

Figure S5. ^{1}H NMR (400 MHz, CD_3OD) of compound **1**

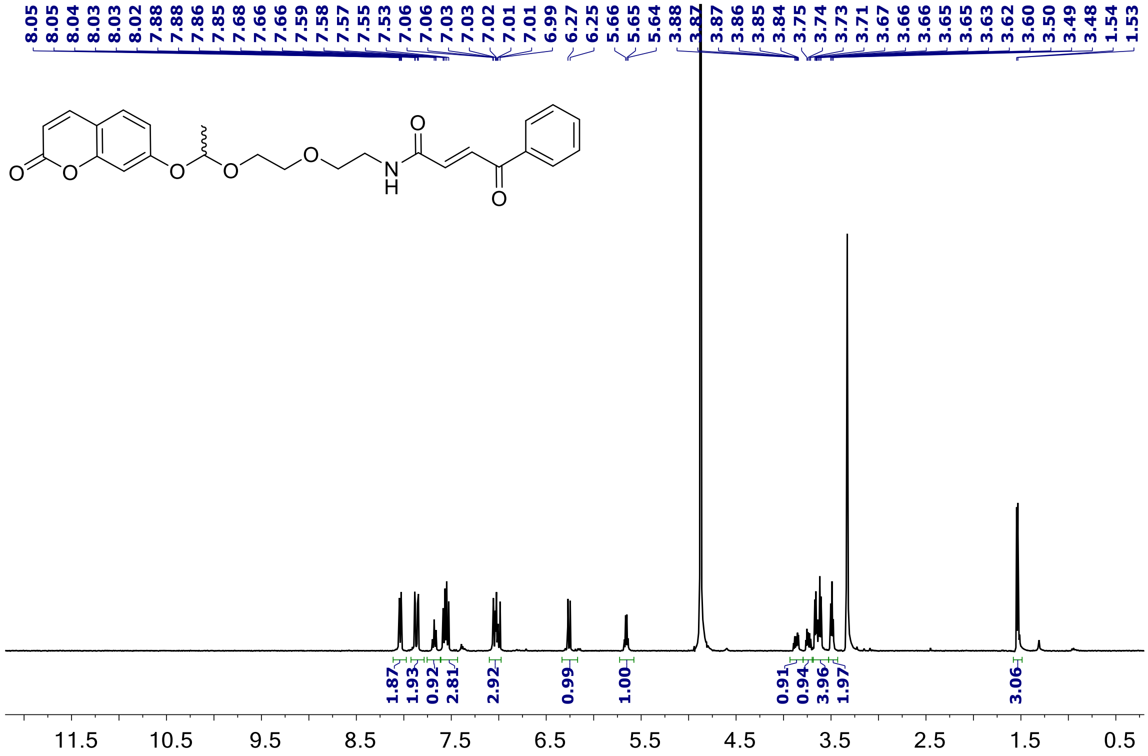

Figure S6. $^{13}C\{^{1}H\}$ NMR (100 MHz, CD_3OD) of compound **1**

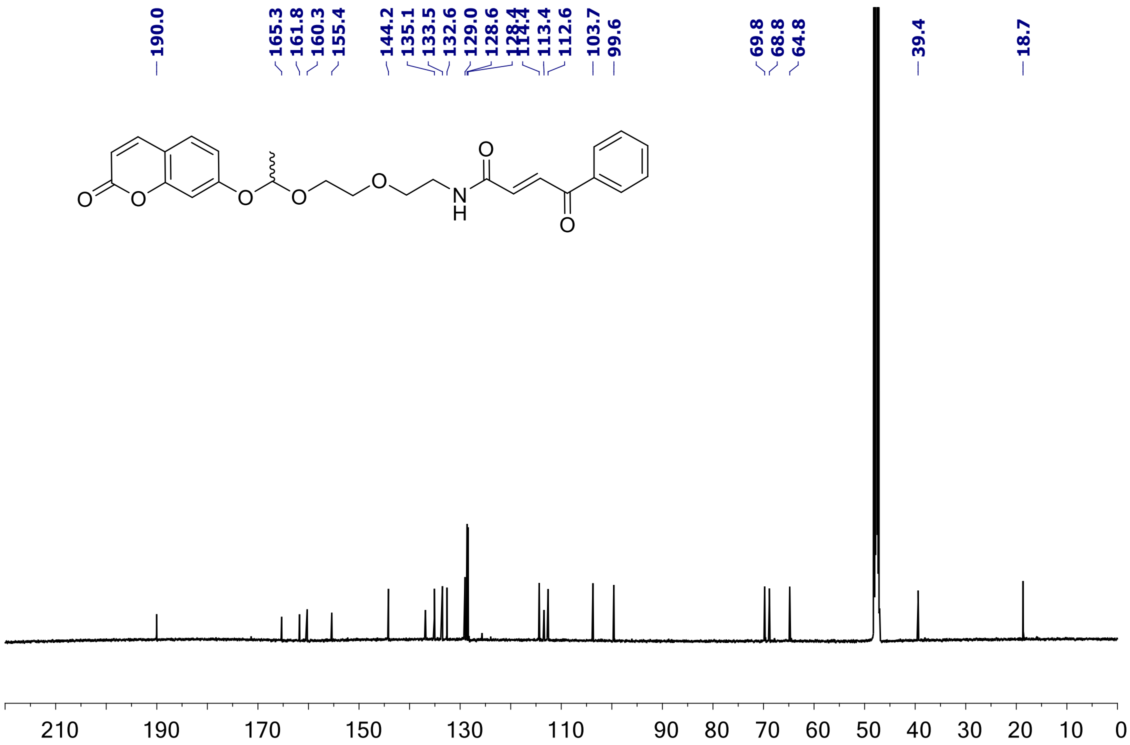

Figure S7. ^{1}H NMR (400 MHz, $CDCl_3$) of compound **8**

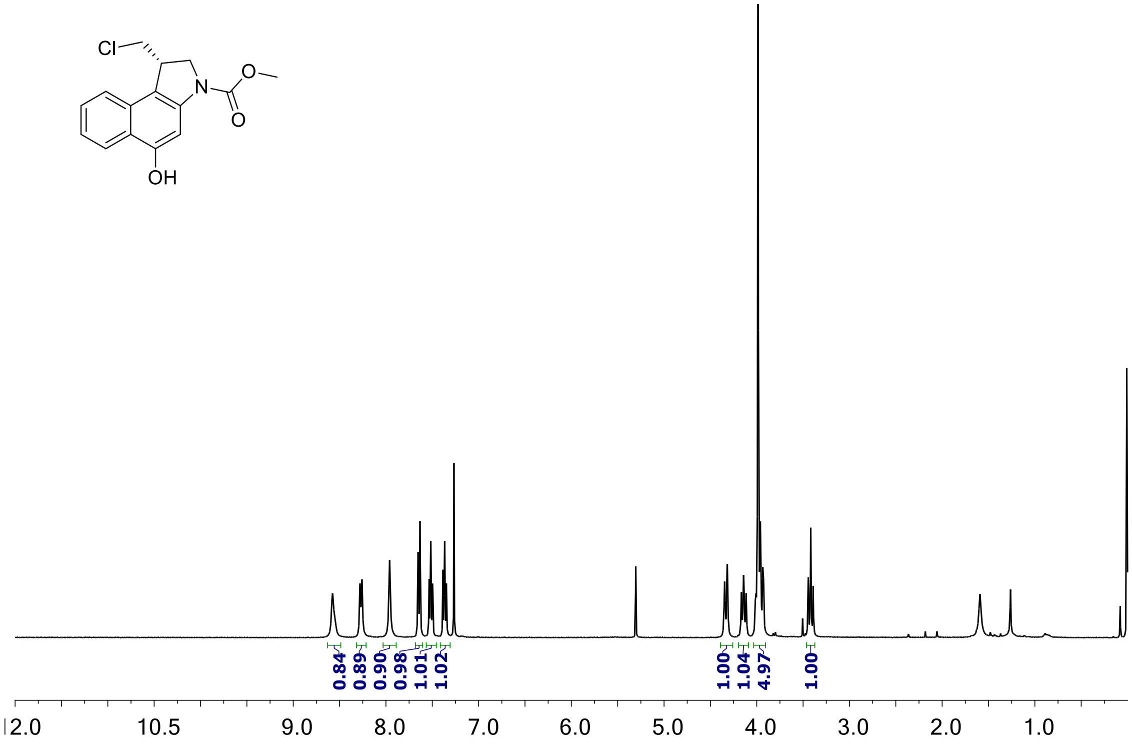

Figure S8. $^{13}C\{^{1}H\}$ NMR (75 MHz, $CDCl_3$) of compound **8**

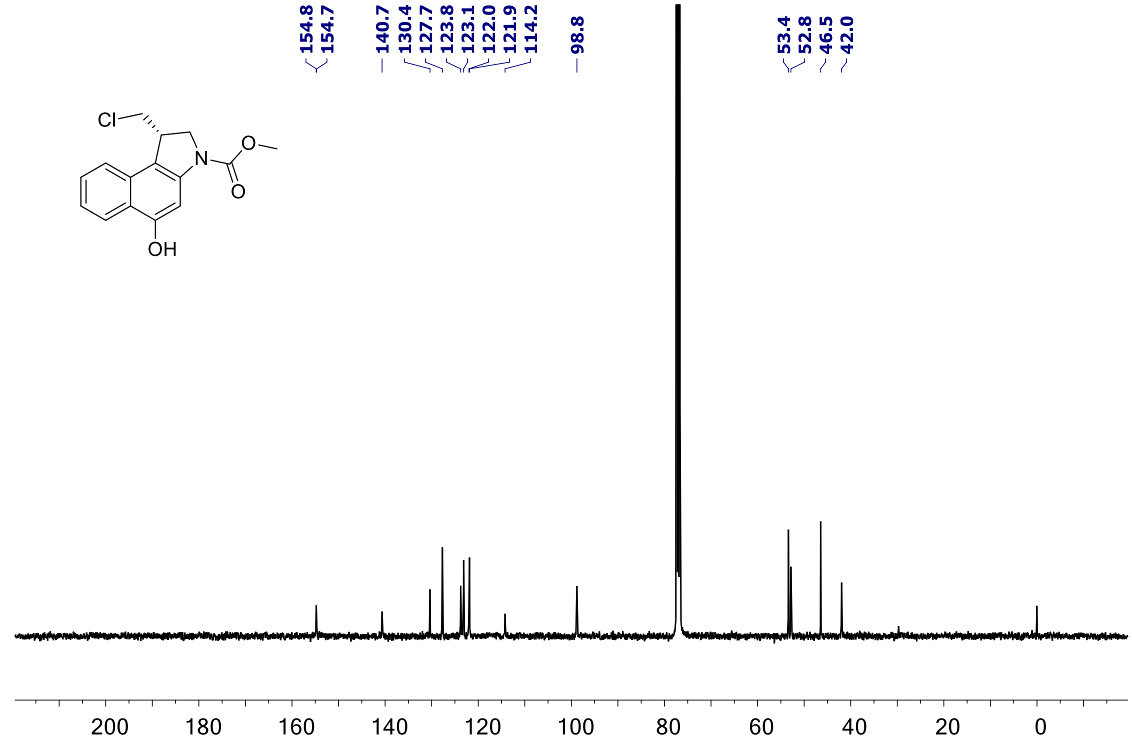

Figure S9. ^{1}H NMR (300 MHz, CD_3OD) of duocarmycin derivative **I**

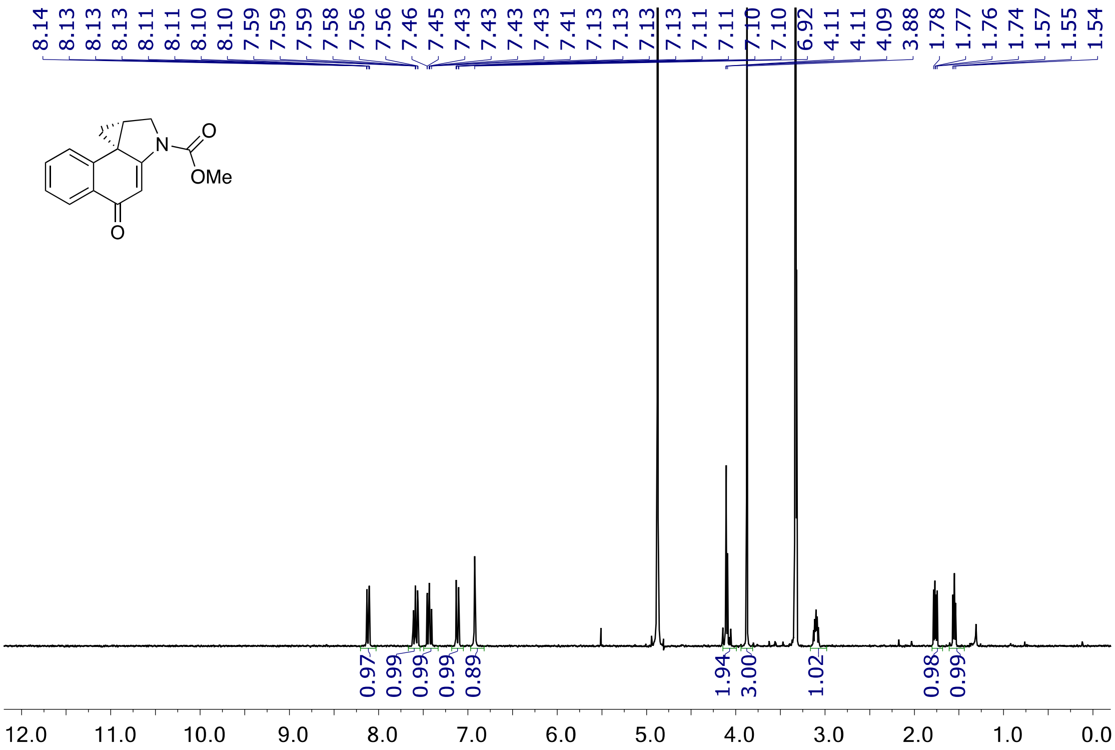

Figure S10. $^{13}C\{^{1}H\}$ NMR (75 MHz, CD_3OD) of duocarmycin derivative **I**

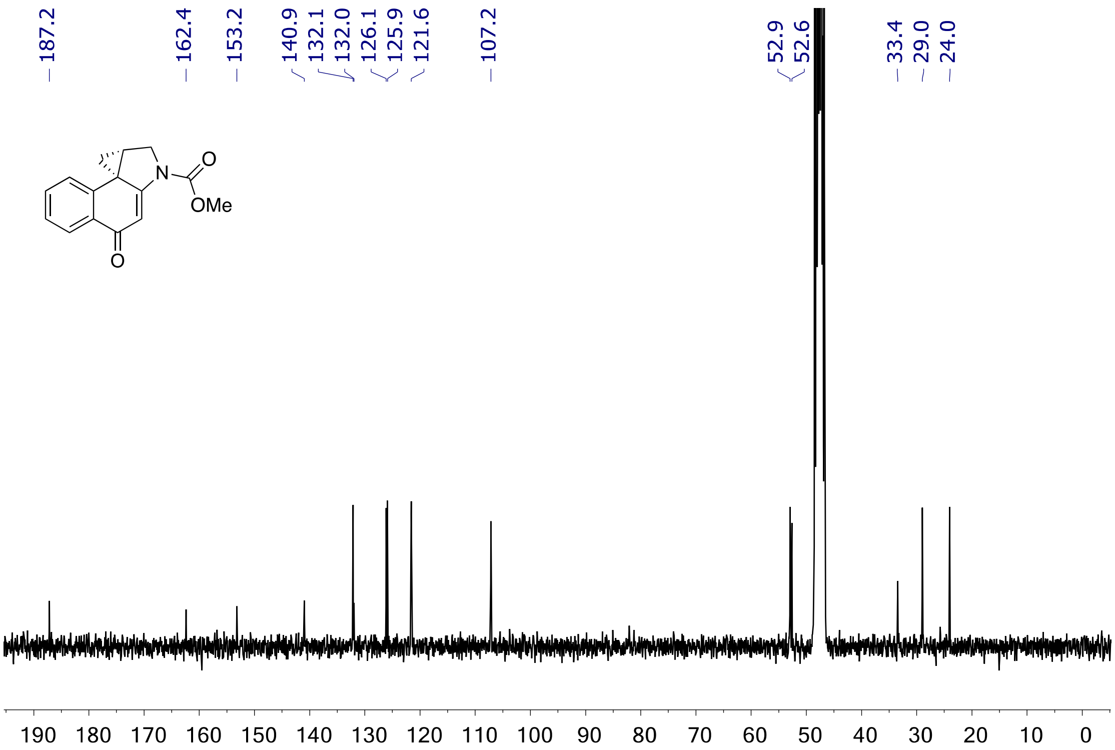

Figure S11. ^{1}H NMR (400 MHz, CD_3OD) of compound **9**

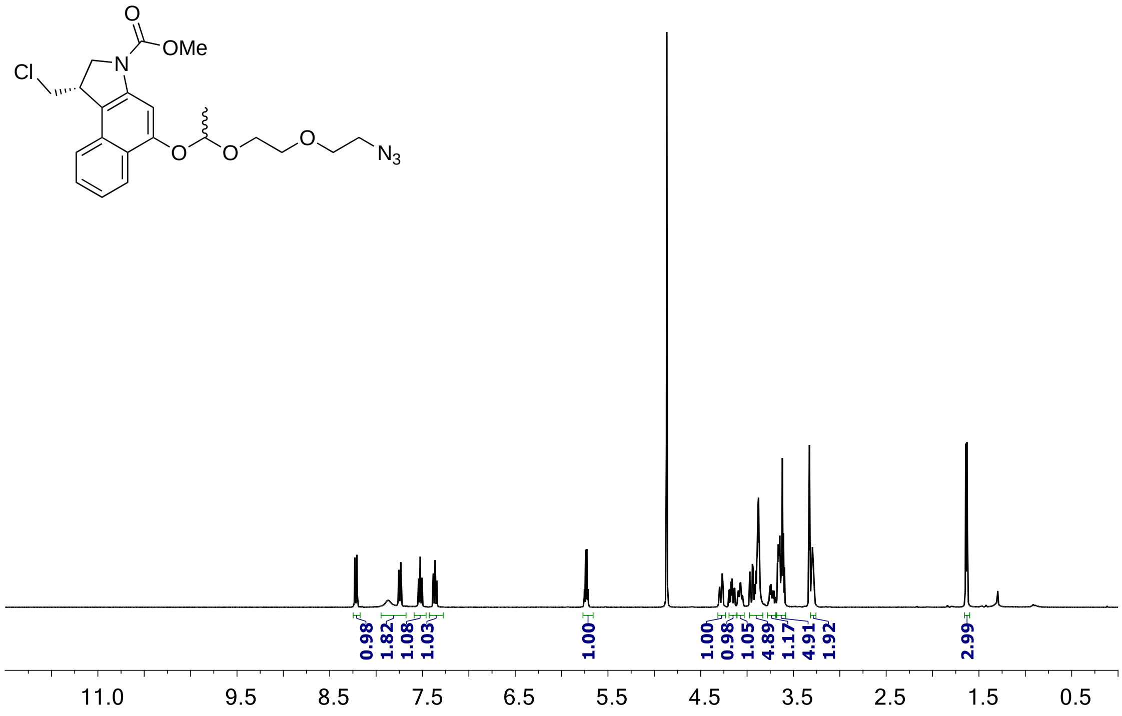

Figure S12. $^{13}C\{^{1}H\}$ NMR (75 MHz, CD_3OD) of compound **9**

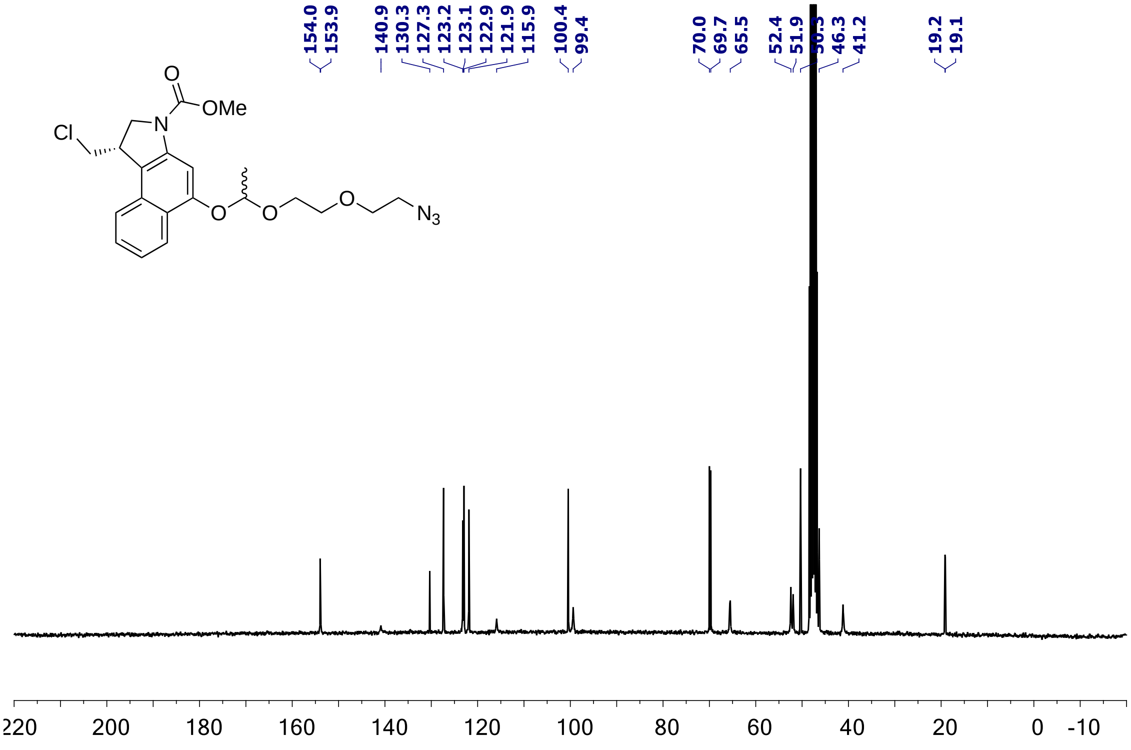

Figure S13. ^{1}H NMR (400 MHz, CD_3OD) of compound **5** (as a mixture of diastereomers)

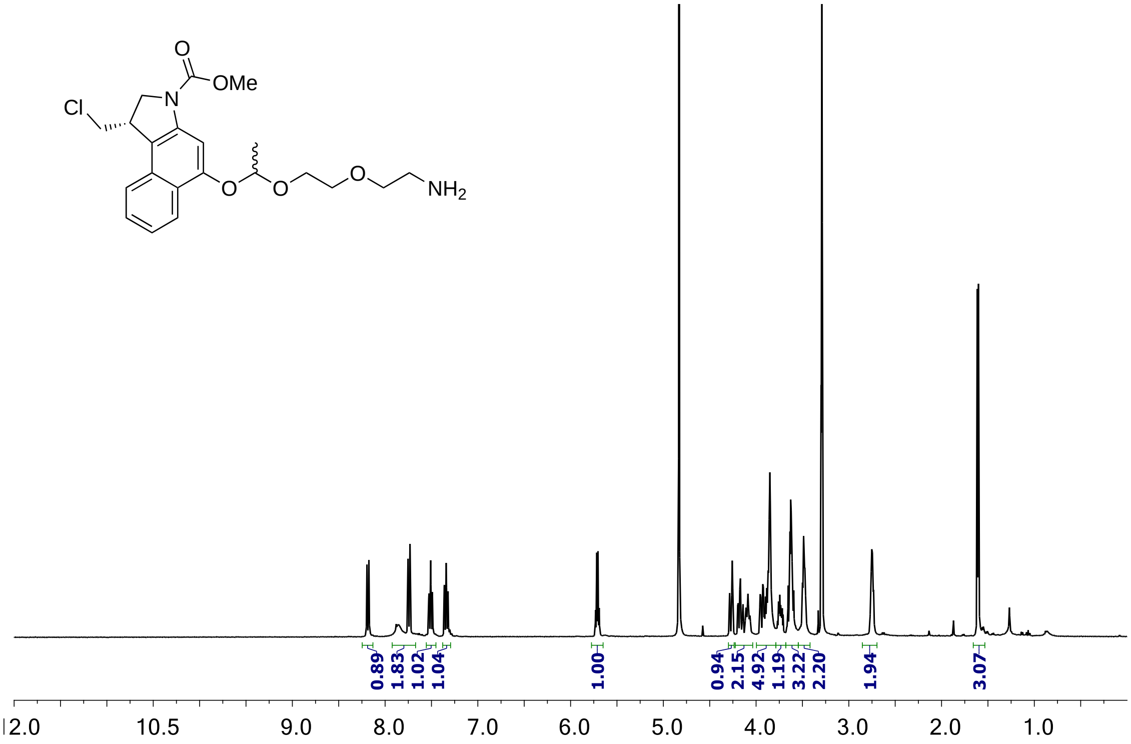

Figure S14. ^{13}C{^{1}H} NMR (75 MHz, CD_3OD) of compound **5** (as a mixture of diastereomers)

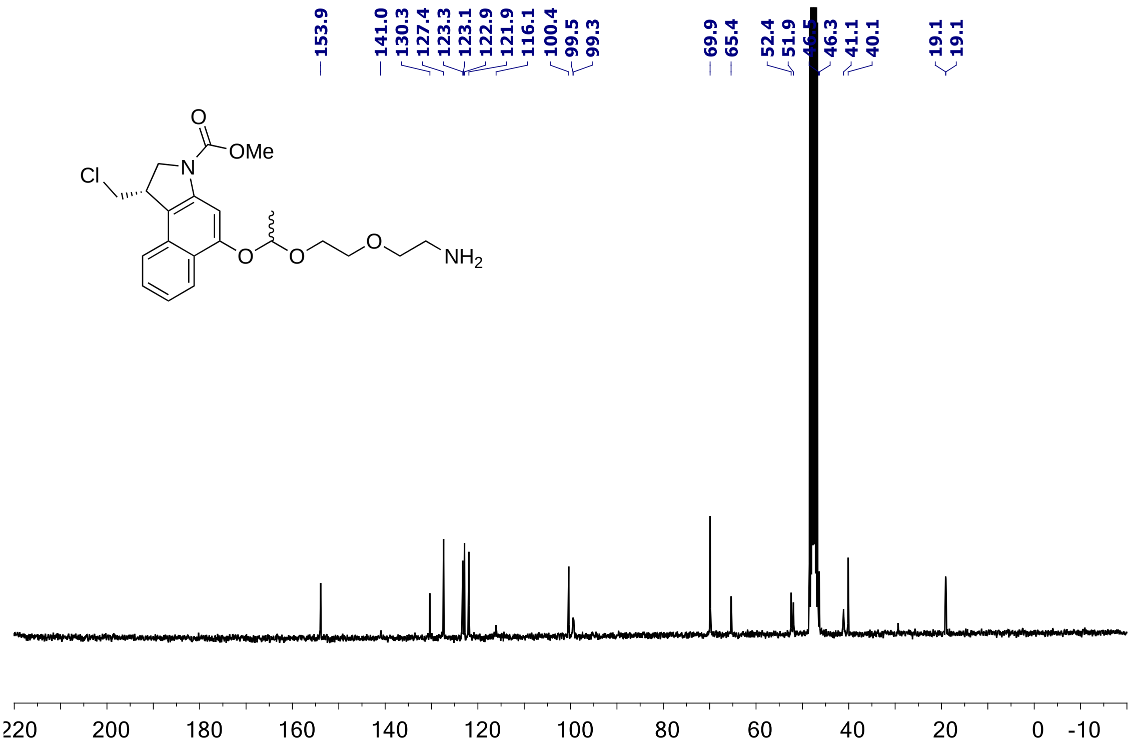

Figure S15. ^{1}H NMR (400 MHz, CD3OD) of compound **2** (as a mixture of diastereomers)

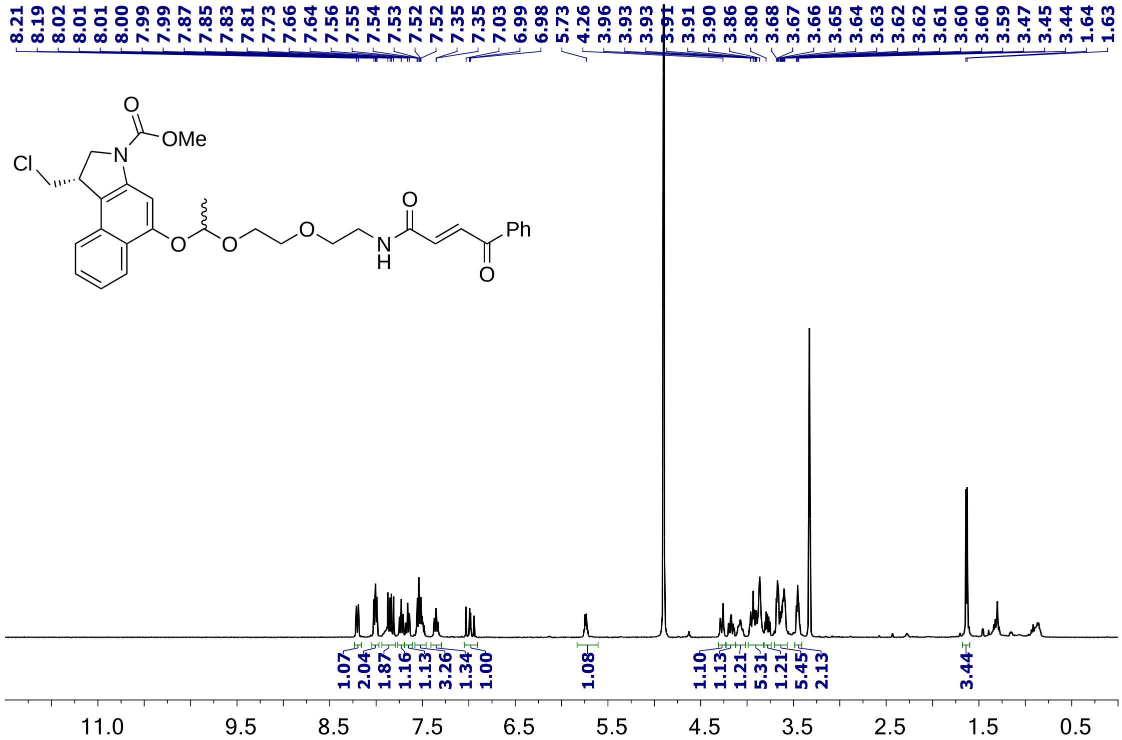

Figure S16. ^{13}C{^{1}H} NMR (100 MHz, CD_3OD) of compound **2** (as a mixture of diastereomers)

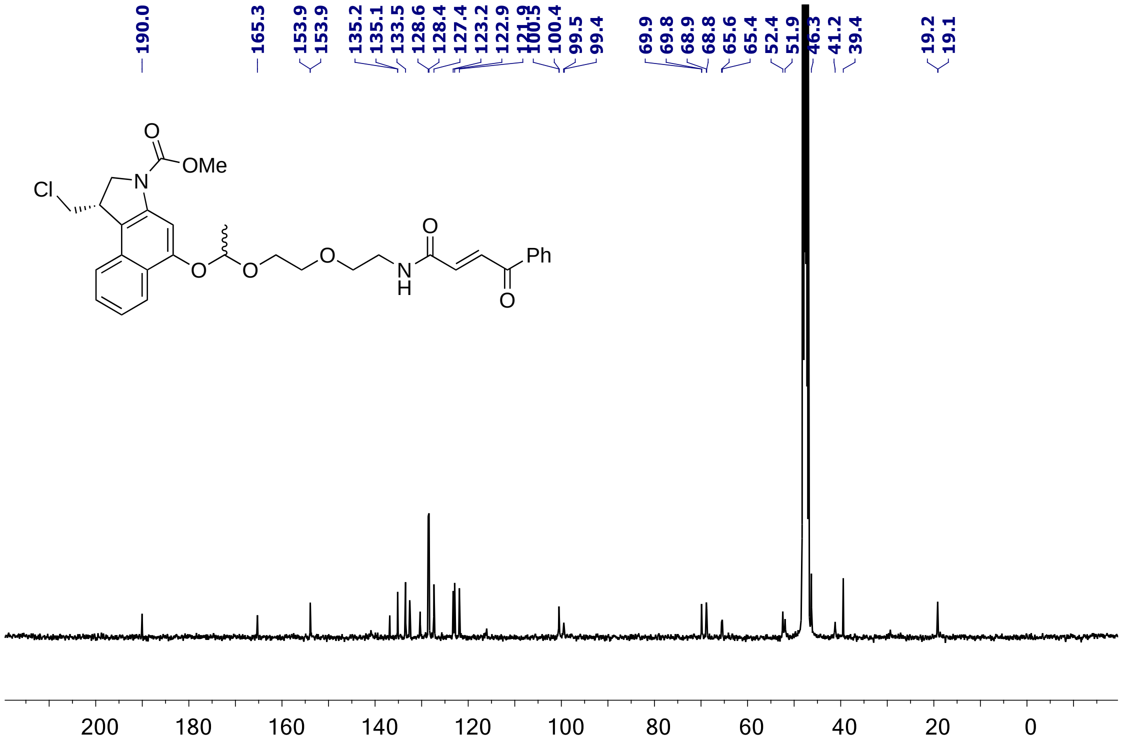

Figure S17. ^{1}H NMR (300 MHz, CD_3OD) of compound **S6** (as a mixture of diastereomers)

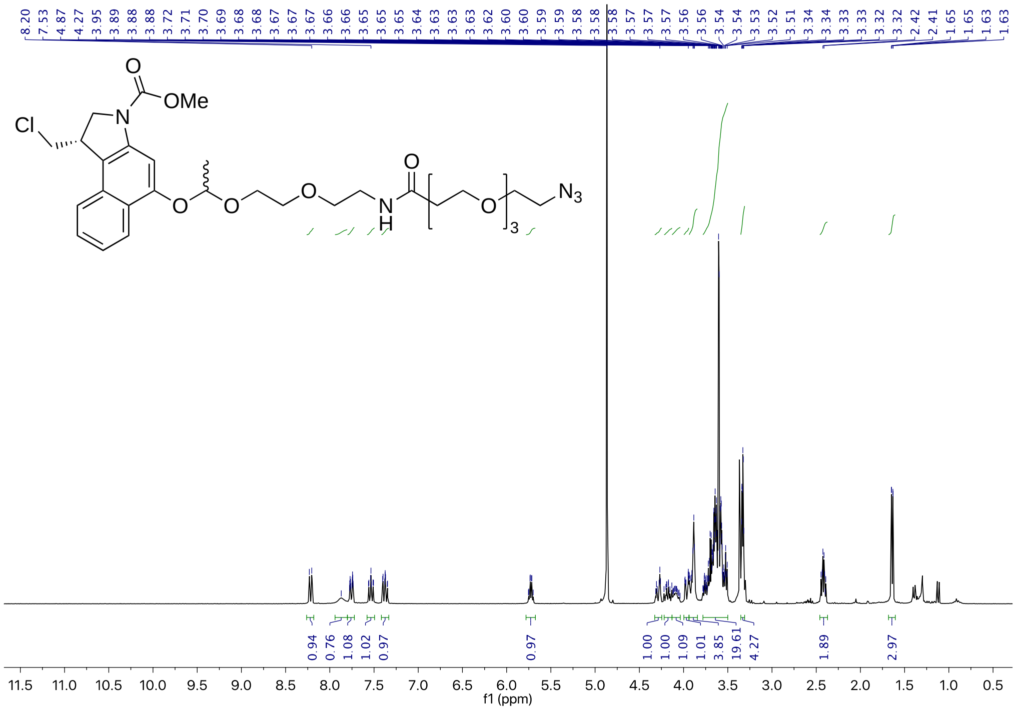

Figure S18. $^{13}C\{^{1}H\}$ NMR (75 MHz, CD_3OD) of compound **S6** (as a mixture of diastereomers)

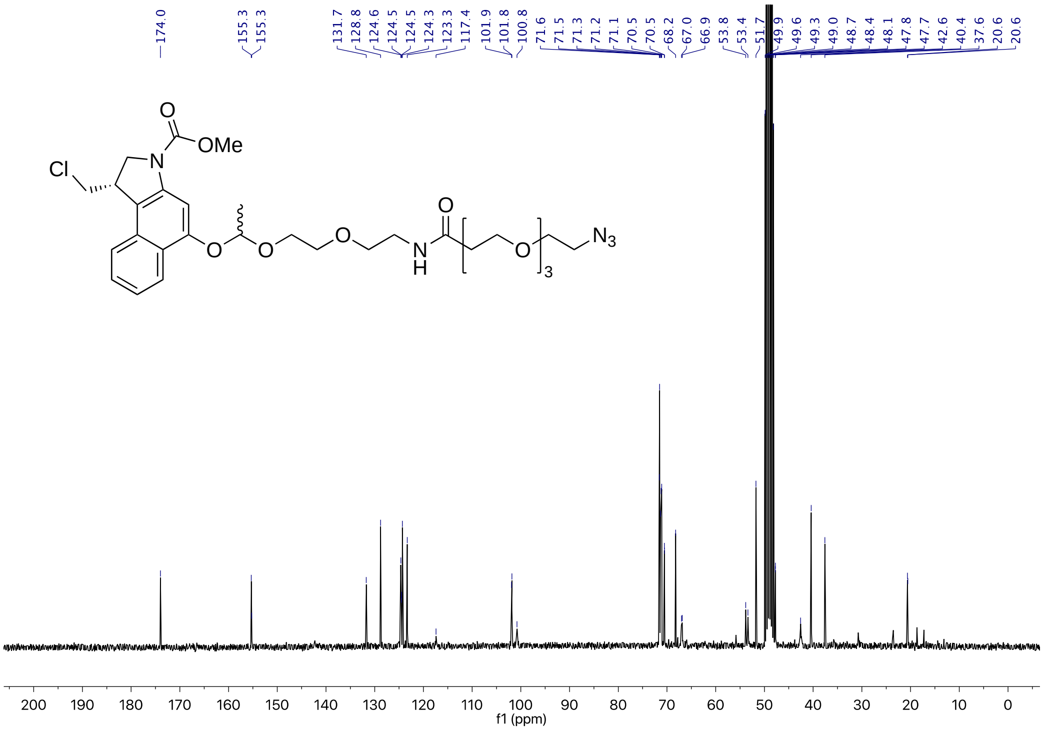

Figure S19. ^{1}H NMR (400 MHz, CD_3OD) of compound **3**

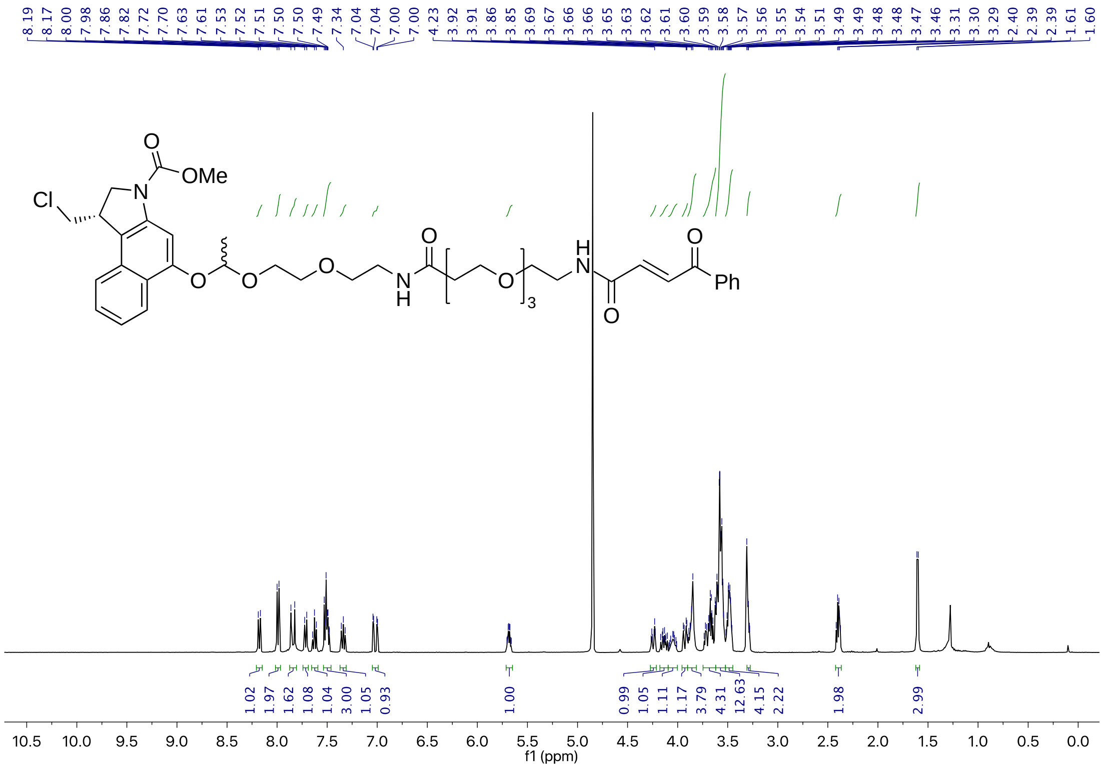

Figure S20. $^{13}C\{^{1}H\}$ NMR (101 MHz, CD_3OD) of compound **3**

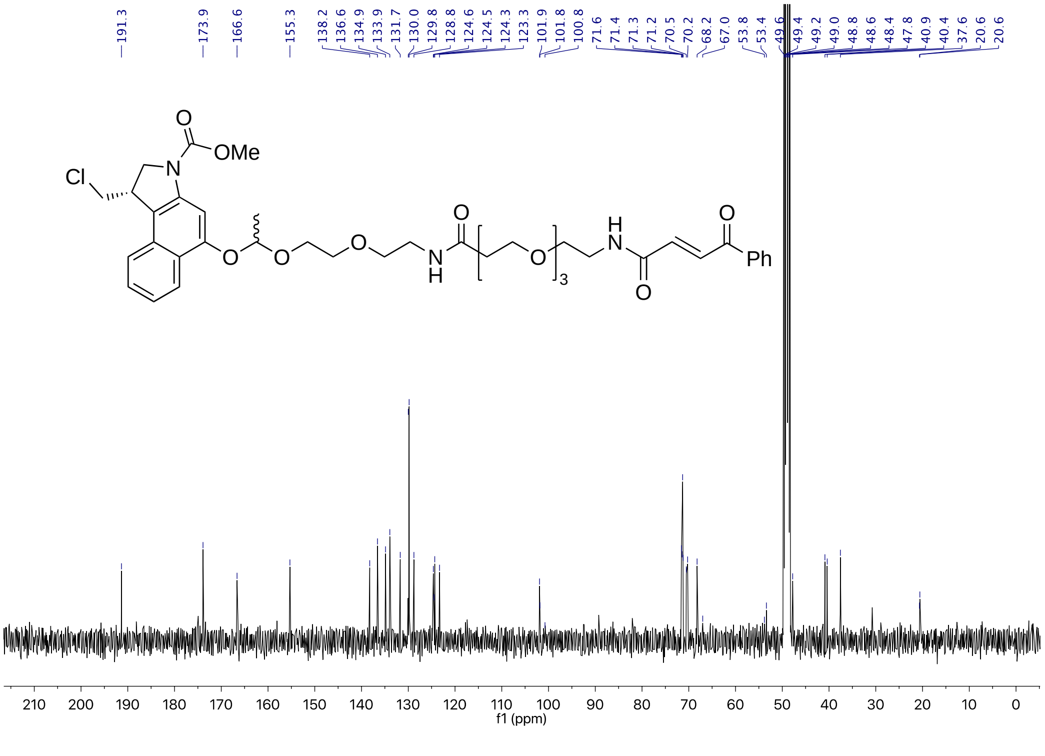

Figure S21. ^{1}H NMR (400 MHz, CD_3OD) of compound **4**

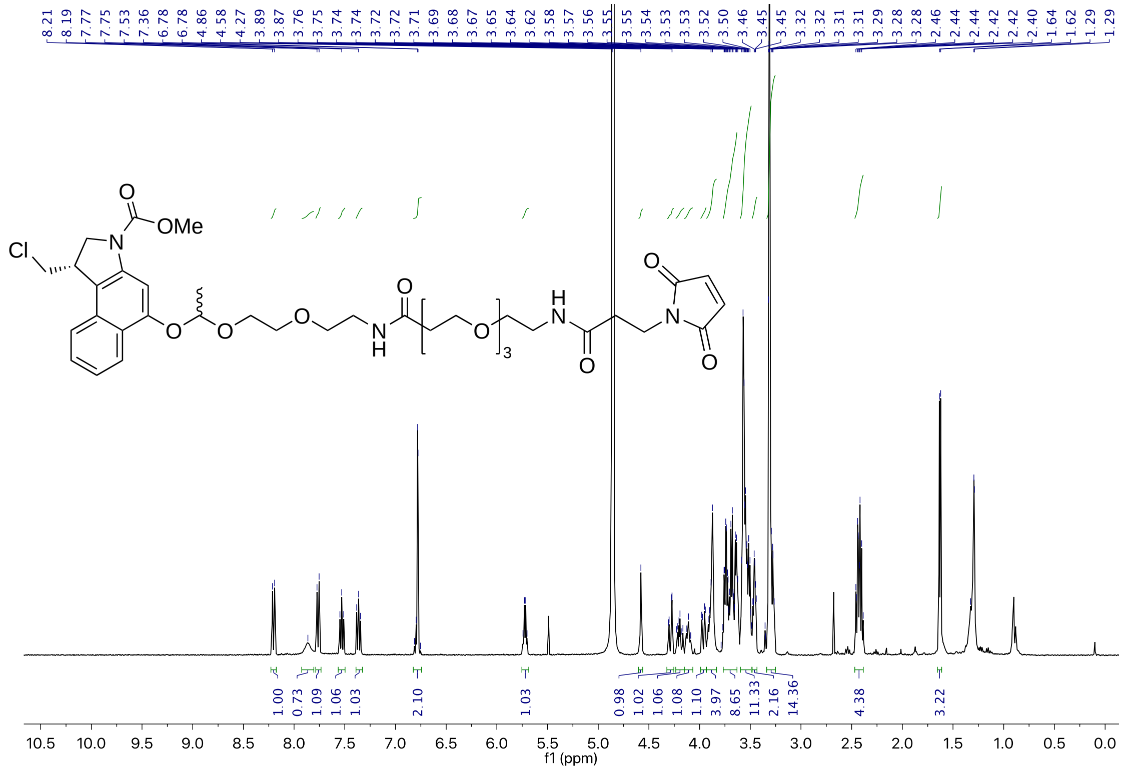

Figure S22. $^{13}C\{^{1}H\}$ NMR (100 MHz, CD_3OD) of compound **4**

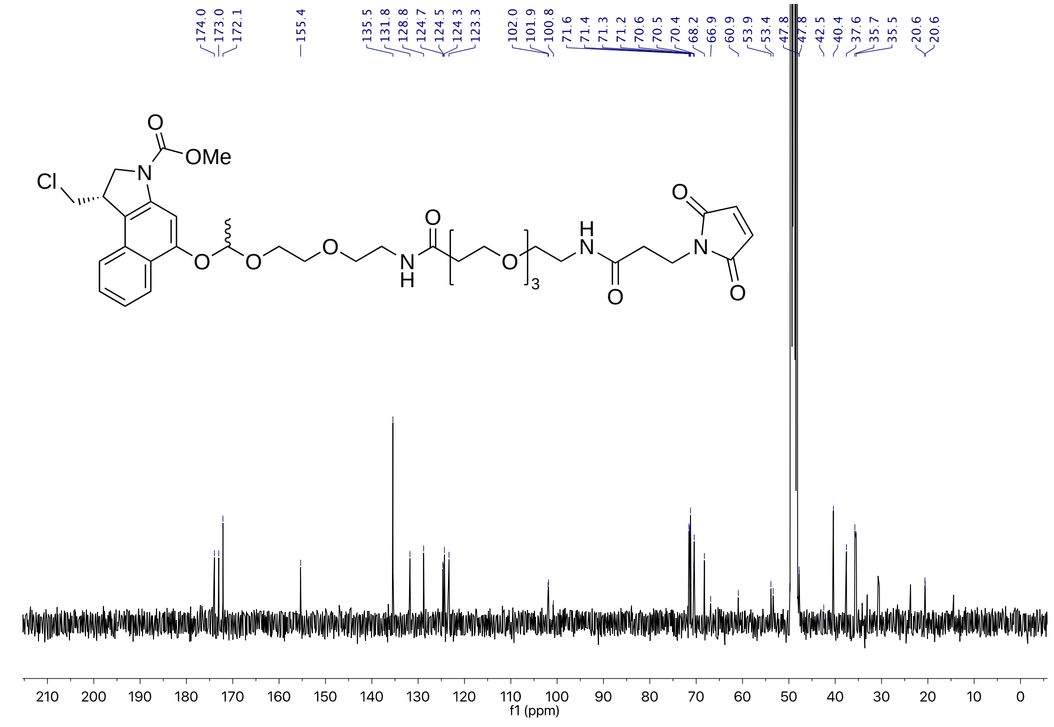

Quantum Mechanical calculations

Full geometry optimizations and transition structure (TS) searches were carried out with Gaussian 16[2] using the M06-2X hybrid functional[3] and 6-31+G(d,p) basis set with ultrafine integration grids. Bulk solvent effects in water were considered implicitly through the IEF-PCM polarizable continuum model.[4] The possibility of different conformations was considered for all structures. All stationary points were characterized by a frequency analysis performed at the same level used in the geometry optimizations from which thermal corrections were obtained at 298.15 K. The quasiharmonic approximation reported by Truhlar *et al.* was used to replace the harmonic oscillator approximation for the calculation of the vibrational contribution to enthalpy and entropy.[5] Scaled frequencies were not considered. Mass-weighted intrinsic reaction coordinate (IRC) calculations were carried out by using the Gonzalez and Schlegel scheme[6,7] to ensure that the TSs indeed connected the appropriate reactants and products. Gibbs free energies (ΔG) were used for the discussion on the relative stabilities of the considered structures. The lowest energy conformer for each calculated stationary point (Scheme S6 and Figure S23) was considered in the discussion; all the computed structures can be obtained from authors upon request. Cartesian coordinates, electronic energies, entropies, enthalpies, Gibbs free energies, and lowest frequencies of the calculated structures are summarized in Table S1. Cartesian coordinates of the lowest energy structures calculated with PCM(H_2O)/M06-2X/6-31+G(d,p) are shown in Table S2.

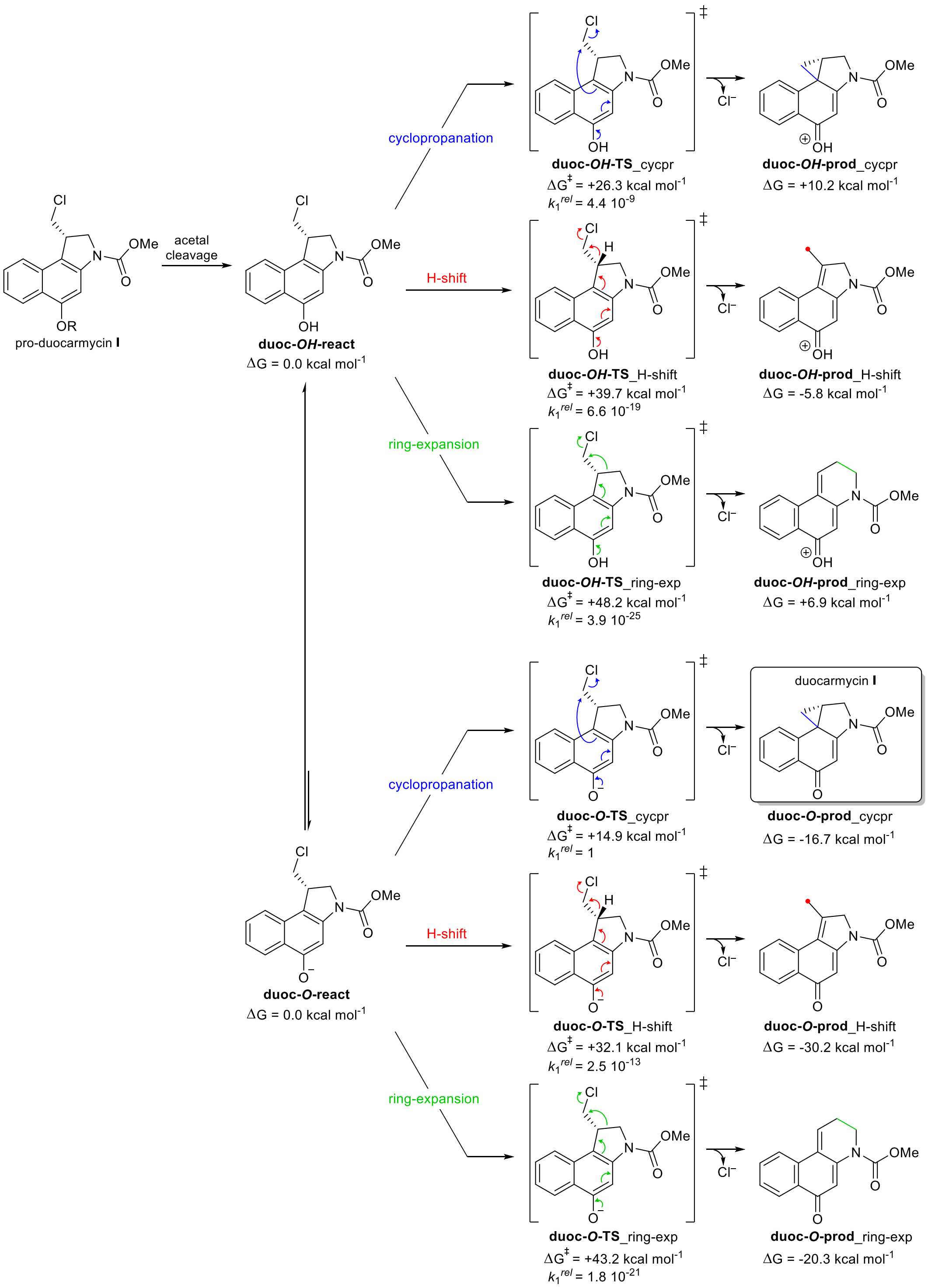

Scheme S6. All possible reaction pathways studied computationally from pro-duocarmycin I. After acetal cleavage, three reactions (cyclopropanation, hydrogen shift and ring-expansion) from either the protonated or deprotonated forms of the intermediate were analyzed. The most feasible reaction was computed to be, by far, cyclopropanation from the napthoxide leading to duocarmycin.

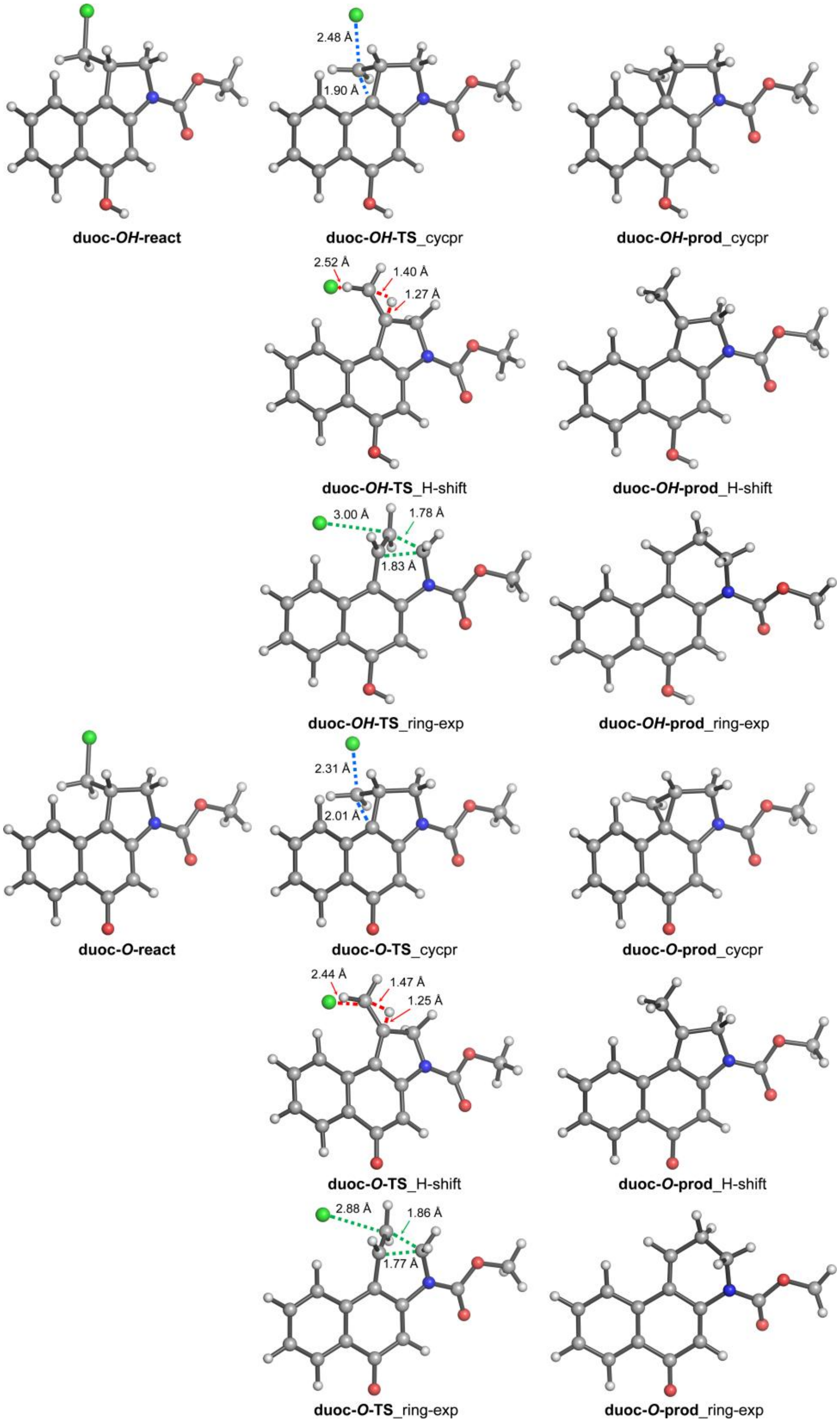

Figure S23. Guide to compound numbering of calculated structures (only the lowest energy conformers are shown).

Table S1. Energies, entropies, and lowest frequencies of the lowest energy calculated structures.[a]

Structure	E_{elec} (Hartree)	E_{elec} + ZPE (Hartree)	H (Hartree)	S (cal mol^{-1} K^{-1})	G (Hartree)	Lowest freq. (cm^{-1})	# of imag freq.
**duoc-*OH*-react**	-1320.365451	-1320.096238	-1320.077823	136.0	-1320.140081	35.8	0
**duoc-*OH*-TS**_cycpr	-1320.321592	-1320.054487	-1320.036129	135.8	-1320.098244	-548.7	1
**duoc-*OH*-prod**_cycpr	-859.989109	-859.720984	-859.704648	124.0	-859.762256	41.6	0
**duoc-*OH*-TS**_H-shift	-1320.296544	-1320.032783	-1320.014034	139.4	-1320.076784	-919.1	1
**duoc-*OH*-prod**_H-shift	-860.011857	-859.745649	-859.728371	129.1	-859.787774	33.7	0
**duoc-*OH*-TS**_ring-exp	-1320.283933	-1320.018696	-1319.999592	141.8	-1320.063210	-323.8	1
**duoc-*OH*-prod**_ring-exp	-859.994390	-859.726106	-859.709484	127.1	-859.767544	20.6	0
**duoc-*O*-react**	-1319.892466	-1319.636899	-1319.618768	135.3	-1319.680645	35.6	0
**duoc-*O*-TS**_cycpr	-1319.867119	-1319.613280	-1319.595224	134.8	-1319.656884	-565.6	1
**duoc-*O*-prod**_cycpr	-859.559708	-859.304495	-859.288371	123.5	-859.345701	39.1	0
**duoc-*O*-TS**_H-shift	-1319.836184	-1319.585614	-1319.567222	137.7	-1319.629480	-984.1	1
**duoc-*O*-prod**_H-shift	-859.578708	-859.325140	-859.308057	128.8	-859.367232	29.1	0
**duoc-*O*-TS**_ring-exp	-1319.819801	-1319.567656	-1319.548979	139.9	-1319.611878	-324.2	1
**duoc-*O*-prod**_ring-exp	-859.565521	-859.310068	-859.293651	126.3	-859.351470	25.6	0
**Cl-**	-460.346502	-460.346502	-460.344142	36.6	-460.361525		0

[a]Energy values calculated at the PCM(H_2O)/M06-2X/6-31+G(d,p) level.

1 Hartree = 627.51 kcal mol^{-1}. Thermal corrections at 298.15 K.

observed rate | cyclopropanation kinetics | deprotonation thermodynamics

$$k_{obs} = k_{duocarmycin-O^-}\left(\frac{K_a}{K_a + [H^+]}\right)$$

Equation S1. Effect of pH on the apparent reaction rate constant for duocarmycin formation (k_{obs}). The intrinsic reaction rate constants for pro-duocarmycin cyclopropanation ($k_{duocarmycin-O^-}$) is affected by the acidity of the phenol (K_a) group and the environmental pH ($[H^+]$).[8]

Table S2. Cartesian coordinates of the lowest energy structures calculated with PCM(H_2O)/M06-2X/6-31+G(d,p).

Structure **duoc-*OH*-react**

C	-4.703092	0.087525	0.151630
C	-3.932582	-1.024419	-0.094355
C	-2.517114	-0.944916	-0.041938
C	-1.891639	0.299447	0.267499
C	-2.718085	1.429101	0.522778
C	-4.087072	1.324339	0.465487
H	-5.785173	0.020465	0.109423
H	-4.395033	-1.976500	-0.331200
H	-2.255545	2.379449	0.774510
H	-4.703772	2.195074	0.664990
C	-0.317684	-2.025550	-0.238730
H	0.294225	-2.898302	-0.426637
C	0.268507	-0.779746	0.074600
C	-0.479079	0.352245	0.316641
C	-1.693092	-2.086011	-0.292671
C	0.431498	1.543312	0.517034
H	0.140311	2.171253	1.364075
C	1.795264	0.873357	0.774641
H	2.622871	1.395164	0.292784
H	2.003037	0.791322	1.845757
N	1.637060	-0.479757	0.203206
C	2.663376	-1.357967	0.036382
O	2.554059	-2.503168	-0.372716
C	0.429379	2.382589	-0.759937
H	0.845817	1.821172	-1.598568
H	-0.573404	2.725466	-1.013753
Cl	1.444445	3.867262	-0.573879
O	-2.352593	-3.238483	-0.581738
H	-1.723720	-3.955311	-0.738315
O	3.833041	-0.793001	0.376800
C	4.986736	-1.627099	0.213427
H	5.092656	-1.924876	-0.830780
H	5.831168	-1.015863	0.523824
H	4.903596	-2.513698	0.843690

Structure **duoc-*OH*-TS**_cycpr

C	-4.720441	0.053631	0.235532
C	-3.925647	-1.005602	-0.144338
C	-2.517302	-0.891113	-0.092653
C	-1.922978	0.319184	0.347382
C	-2.758303	1.390604	0.733434
C	-4.129733	1.255851	0.679563
H	-5.800587	-0.035235	0.197584
H	-4.366816	-1.936196	-0.483127
H	-2.316619	2.319684	1.081935
H	-4.761505	2.083517	0.985229
C	-0.283275	-1.934258	-0.392030
H	0.328229	-2.790577	-0.641772
C	0.289154	-0.736655	0.055220
C	-0.492856	0.392840	0.362553
C	-1.665090	-1.988410	-0.468783
C	0.445625	1.511439	0.795933
H	0.100345	2.196426	1.565168
C	1.779074	0.817722	1.034274
H	2.608133	1.394542	0.623239
H	1.952859	0.615773	2.094102
N	1.622751	-0.462331	0.302393
C	2.668932	-1.331319	0.069032
O	2.563660	-2.405546	-0.487655
C	0.210505	1.872519	-0.591279
H	0.803562	1.419744	-1.375759
H	-0.673795	2.430659	-0.867959
Cl	1.553620	3.928030	-0.956061
O	-2.301722	-3.091222	-0.883527
H	-1.675334	-3.788581	-1.124526
O	3.805754	-0.820979	0.540581
C	4.973401	-1.634804	0.344950
H	5.139058	-1.797799	-0.720556
H	5.793339	-1.069008	0.779990
H	4.850395	-2.590935	0.854785

Structure **duoc-*OH*-prod**_cycpr

C	-4.536839	0.101713	-0.360446
C	-3.587155	1.093123	-0.188374
C	-2.234562	0.743255	-0.024854
C	-1.845062	-0.610578	-0.030342
C	-2.816462	-1.601174	-0.197776
C	-4.147661	-1.245159	-0.368224
H	-5.580048	0.366184	-0.491458
H	-3.872272	2.138843	-0.182635
H	-2.532137	-2.649094	-0.205001

H	-4.892907	-2.020692	-0.510583
C	0.151269	1.500136	0.182705
H	0.873124	2.305013	0.184544
C	0.539865	0.168648	0.130909
C	-0.430446	-0.917095	0.212709
C	-1.215321	1.766311	0.129337
C	0.348706	-2.207319	-0.058461
H	-0.111797	-2.997174	-0.639585
C	1.768660	-1.787240	-0.381843
H	2.505590	-2.321928	0.218001
H	2.007944	-1.897373	-1.441477
N	1.784721	-0.344431	-0.031246
C	2.962854	0.413087	-0.027484
O	3.004403	1.596479	0.215663
C	-0.072433	-1.938891	1.324569
O	-1.669951	3.003505	0.157622
H	0.666804	-1.622502	2.054563
H	-0.936713	-2.463624	1.714601
H	-0.957278	3.653663	0.259366
O	3.994350	-0.360247	-0.325679
C	5.274411	0.299766	-0.350024
H	5.991053	-0.477940	-0.599365
H	5.269548	1.082874	-1.108453
H	5.486685	0.725370	0.630951

Structure **duoc-*OH*-TS**_H-shift

C	-4.447924	-0.960233	0.041178
C	-3.432511	-1.876475	-0.113700
C	-2.080322	-1.470845	-0.021058
C	-1.758179	-0.110231	0.250148
C	-2.826900	0.813155	0.371216
C	-4.136327	0.395912	0.272919
H	-5.483523	-1.274612	-0.030221
H	-3.653272	-2.918561	-0.315295
H	-2.623557	1.867731	0.502280
H	-4.935711	1.124282	0.363469
C	0.310672	-2.059413	-0.179930
H	1.098284	-2.779416	-0.355423
C	0.609036	-0.716060	0.110698
C	-0.377423	0.241028	0.347622
C	-1.018936	-2.417659	-0.219762
C	0.296966	1.478884	0.730920
H	0.176863	1.732339	1.973700
C	1.763313	1.300362	0.391998
H	1.968457	1.826854	-0.548186
H	2.442154	1.660671	1.164212
N	1.878655	-0.147112	0.205677
C	3.075581	-0.784768	-0.002881
O	3.206104	-1.977213	-0.201187
C	-0.199347	2.746351	1.078064
H	-1.232647	2.897779	1.350043
H	0.512392	3.525823	1.323281
Cl	-0.703820	3.951455	-1.078277
O	-1.408549	-3.682936	-0.467944
H	-0.643026	-4.261253	-0.590658
O	4.080869	0.094030	0.050200
C	5.392449	-0.459971	-0.134575
H	5.464656	-0.929392	-1.116303
H	6.073503	0.384293	-0.061295
H	5.601489	-1.192770	0.645671

Structure **duoc-*OH*-prod**_H-shift

C	-4.549007	-0.210925	-0.000610
C	-3.578427	-1.198368	-0.000354
C	-2.218694	-0.846269	-0.000034
C	-1.830103	0.514287	0.000044
C	-2.829434	1.497840	-0.000158
C	-4.170056	1.136596	-0.000491
H	-5.598914	-0.481750	-0.000898
H	-3.856709	-2.245742	-0.000426
H	-2.568489	2.547481	-0.000087
H	-4.929100	1.911656	-0.000666
C	0.161889	-1.622123	0.000388
H	0.888655	-2.422608	0.000487
C	0.545917	-0.283212	0.000412
C	-0.404854	0.809846	0.000274
C	-1.199001	-1.882411	0.000254
C	0.296138	1.984051	0.000233
H	-0.791154	3.590947	0.884761
C	1.755355	1.684693	0.000536
H	2.251855	2.093792	-0.886937
H	2.251303	2.093903	0.888291
N	1.803928	0.228081	0.000664
C	2.992074	-0.508267	0.000016
O	3.037260	-1.715736	-0.000275
C	-0.177465	3.393437	0.000111
H	-0.791084	3.590830	-0.884585
H	0.665050	4.085619	0.000081
O	-1.664758	-3.120852	0.000422
H	-0.950063	-3.775832	0.000720
O	4.027526	0.316611	-0.000182
C	5.322202	-0.314656	-0.000923
H	5.431636	-0.927934	-0.895567
H	6.037312	0.503418	-0.001502
H	5.432778	-0.927651	0.893779

Structure **duoc-*OH*-TS**_ring-exp

C	4.371202	-1.129691	-0.338342
C	3.340234	-1.957376	0.039105
C	2.001797	-1.491802	0.007374
C	1.729407	-0.161011	-0.412951
C	2.814994	0.677043	-0.781131
C	4.102956	0.197254	-0.751772
H	5.394242	-1.489652	-0.314834
H	3.534062	-2.973170	0.364908
H	2.622844	1.707134	-1.064664
H	4.924212	0.845444	-1.040054
C	-0.401555	-1.902223	0.344159
H	-1.217405	-2.562426	0.604246
C	-0.648031	-0.583308	-0.084227
C	0.378459	0.274919	-0.425630
C	0.908365	-2.331199	0.389188
C	-0.064303	1.619815	-0.834648
H	0.300193	2.050107	-1.765769
C	-1.854851	1.282502	-0.946366
H	-2.710760	1.917727	-0.704207

H	-1.809369	1.228229	-2.037717
N	-1.917487	0.000543	-0.288209
C	-3.127115	-0.571285	0.055272
O	-3.247163	-1.636517	0.622036
C	-0.855067	2.418020	-0.012795
H	-1.097835	3.428310	-0.318687
H	-1.053614	2.128649	1.013616
Cl	1.595435	3.685443	1.178945
O	1.238521	-3.581998	0.778229
H	0.450368	-4.084003	1.026088
O	-4.139328	0.210610	-0.325445
C	-5.452965	-0.280593	-0.008495
H	-5.556292	-0.393221	1.071017
H	-6.137995	0.474494	-0.385273
H	-5.620037	-1.237107	-0.504285

Structure **duoc-*OH*-prod**_ring-exp

C	-4.654453	0.237777	-0.148593
C	-3.646691	1.179773	-0.050088
C	-2.306098	0.760527	0.005445
C	-1.968078	-0.604826	-0.026227
C	-3.003716	-1.540656	-0.157371
C	-4.326079	-1.123478	-0.209192
H	-5.691495	0.550944	-0.192505
H	-3.875321	2.238857	-0.020849
H	-2.782326	-2.598365	-0.241810
H	-5.112583	-1.864059	-0.309319
C	0.114808	1.365020	0.043669
H	0.857608	2.147433	0.052516
C	0.488719	0.020256	-0.003569
C	-0.556962	-1.010195	0.067679
C	-1.227671	1.719071	0.055809
C	-0.204363	-2.270249	0.420873
H	-0.969252	-2.985713	0.706327
C	2.052012	-1.812747	-0.413787
H	3.110366	-2.005859	-0.281049
H	1.791472	-1.964135	-1.466248
N	1.790187	-0.393358	-0.074930
C	2.877472	0.497221	0.069415
O	2.802184	1.596921	0.565961
C	1.214047	-2.702051	0.477396
H	1.299856	-3.740400	0.145892
H	1.559028	-2.675685	1.521056
O	-1.598749	2.985570	0.079882
H	-0.841159	3.591239	0.100191
O	3.992041	-0.041056	-0.401164
C	5.175547	0.766025	-0.257672
H	5.353230	0.975314	0.797373
H	5.979611	0.166438	-0.675737
H	5.054884	1.697207	-0.811491

Structure **duoc-*O*-react**

C	-4.729858	0.039588	0.126268
C	-3.937770	-1.066109	-0.113355
C	-2.529854	-0.993919	-0.052456
C	-1.911943	0.254789	0.264648
C	-2.748876	1.380606	0.511853
C	-4.120760	1.275149	0.444347
H	-5.811620	-0.035114	0.073691
H	-4.380856	-2.027056	-0.357448
H	-2.292104	2.334575	0.765372
H	-4.738525	2.147457	0.638765
C	-0.315803	-2.066692	-0.238856
H	0.305780	-2.932467	-0.420409
C	0.244672	-0.824122	0.076458
C	-0.500940	0.318564	0.328694
C	-1.728005	-2.202155	-0.315473
C	0.401472	1.511043	0.529723
H	0.111400	2.148471	1.371899
C	1.762940	0.845268	0.796670
H	2.596161	1.375861	0.333635
H	1.955706	0.755454	1.870730
N	1.620542	-0.502375	0.210248
C	2.665544	-1.346901	0.024308
O	2.601863	-2.485540	-0.415473
C	0.409585	2.345647	-0.748682
H	0.842898	1.785827	-1.579827
H	-0.592647	2.680255	-1.015414
17	1.410613	3.850691	-0.566549
O	-2.297583	-3.309169	-0.590838
O	3.825904	-0.760027	0.385733
C	4.998648	-1.557218	0.195700
H	5.117888	-1.813173	-0.858470
H	5.827651	-0.938209	0.532531
H	4.938275	-2.470336	0.790085

Structure **duoc-*O*-TS**_cycpr

C	-4.739918	-0.013959	0.209698
C	-3.924693	-1.071871	-0.150602
C	-2.523123	-0.961782	-0.099837
C	-1.935551	0.257956	0.327502
C	-2.783701	1.330600	0.696169
C	-4.157198	1.194960	0.638059
H	-5.819847	-0.111826	0.166745
H	-4.346439	-2.016305	-0.480514
H	-2.346340	2.267402	1.032801
H	-4.791228	2.027620	0.928088
C	-0.263862	-1.992344	-0.368972
H	0.358135	-2.846582	-0.595115
C	0.273131	-0.792635	0.052170
C	-0.510060	0.344238	0.361129
C	-1.688888	-2.126917	-0.482075
C	0.399546	1.481756	0.758240
H	0.061955	2.163311	1.537410
C	1.743258	0.810424	1.014758
H	2.575494	1.396828	0.623662
H	1.897400	0.619087	2.080990
N	1.623943	-0.472376	0.287124
C	2.687745	-1.295876	0.052642
O	2.637722	-2.376173	-0.509282
C	0.227043	1.933751	-0.623728
H	0.793384	1.434626	-1.401094
H	-0.701772	2.411385	-0.907260
17	1.430712	3.892876	-0.890436
O	-2.227268	-3.193980	-0.870014
O	3.820540	-0.750545	0.525187

C	5.007742	-1.523509	0.310987
H	5.815665	-0.937858	0.743769
H	4.924282	-2.489514	0.811073
H	5.172841	-1.673593	-0.756975

Structure **duoc-*O*-prod**_cycpr

C	-4.559788	0.103401	-0.376485
C	-3.613782	1.103502	-0.197064
C	-2.263875	0.778547	-0.016788
C	-1.863102	-0.567089	-0.013042
C	-2.821709	-1.570846	-0.189666
C	-4.158626	-1.237230	-0.374938
H	-5.604454	0.358752	-0.519865
H	-3.897438	2.150927	-0.196786
H	-2.522651	-2.615554	-0.186962
H	-4.892360	-2.023720	-0.520153
C	0.152953	1.540179	0.128018
H	0.867080	2.348683	0.064234
C	0.524279	0.237807	0.121872
C	-0.441849	-0.871066	0.247984
C	-1.264552	1.881893	0.140246
C	0.318157	-2.149879	-0.054267
H	-0.148682	-2.950318	-0.616202
C	1.738667	-1.749370	-0.399654
H	2.481823	-2.306925	0.172339
H	1.945869	-1.862015	-1.467305
N	1.790252	-0.316779	-0.030395
C	2.961952	0.405912	-0.029264
O	3.046646	1.593513	0.213664
C	-0.079974	-1.874866	1.347820
O	-1.637989	3.055407	0.209272
H	0.685935	-1.583002	2.061114
H	-0.925597	-2.418100	1.754515
O	3.998040	-0.383322	-0.328963
C	5.278793	0.264394	-0.341425
H	5.993019	-0.515638	-0.593674
H	5.290665	1.055169	-1.092554
H	5.496182	0.682666	0.642198

Structure **duoc-*O*-TS**_H-shift

C	4.516079	-0.864697	-0.087983
C	3.511476	-1.787167	0.138887
C	2.153956	-1.429261	0.055819
C	1.792940	-0.090285	-0.269855
C	2.841586	0.840641	-0.484894
C	4.167752	0.462127	-0.398453
H	5.559540	-1.155530	-0.021977
H	3.742877	-2.817745	0.389364
H	2.615367	1.876564	-0.701391
H	4.944620	1.202088	-0.566726
C	-0.236410	-2.076344	0.244550
H	-1.004173	-2.809502	0.444596
C	-0.552674	-0.770225	-0.087844
C	0.402376	0.236865	-0.345260
C	1.133360	-2.469860	0.321738
C	-0.298204	1.439090	-0.754075
H	-0.169508	1.711552	-1.964986
C	-1.767866	1.219045	-0.439698
H	-2.017146	1.765846	0.477875
H	-2.437945	1.536356	-1.238947
N	-1.846039	-0.224876	-0.217531
C	-3.029122	-0.875055	-0.023685
O	-3.161659	-2.065678	0.198291
C	0.135419	2.768898	-0.989202
H	1.158788	2.991592	-1.248903
H	-0.619573	3.497526	-1.261706
17	0.446885	3.860894	1.169329
O	1.481971	-3.645954	0.610077
O	-4.056402	-0.012849	-0.118409
C	-5.357382	-0.589785	0.046968
H	-6.055627	0.237283	-0.059902
H	-5.536197	-1.343845	-0.720974
H	-5.446806	-1.041333	1.036045

Structure **duoc-*O*-prod**_H-shift

C	4.573858	-0.230111	-0.000309
C	3.600709	-1.220534	-0.000254
C	2.243062	-0.885634	-0.000077
C	1.848527	0.468733	0.000043
C	2.842377	1.460533	0.000008
C	4.188366	1.113400	-0.000170
H	5.625630	-0.496592	-0.000449
H	3.870054	-2.271507	-0.000338
H	2.572260	2.508310	0.000153
H	4.940858	1.895434	-0.000195
C	-0.169921	-1.657212	0.000387
H	-0.891636	-2.460962	0.000576
C	-0.534104	-0.347885	0.000405
C	0.416290	0.768197	0.000207
C	1.239127	-1.997391	0.000022
C	-0.269875	1.937031	0.000201
H	0.817637	3.560758	-0.885034
C	-1.742698	1.662001	0.000329
H	-2.233383	2.079533	0.887094
H	-2.233258	2.079165	-0.886694
N	-1.808467	0.202545	0.000619
C	-2.988212	-0.503115	0.000223
O	-3.074455	-1.714782	0.000352
C	0.208362	3.352044	-0.000144
H	0.818011	3.561098	0.884373
H	-0.637641	4.041745	-0.000021
O	1.618089	-3.174002	-0.000167
O	-4.028935	0.335767	-0.000378
C	-5.321128	-0.288953	-0.000796
H	-6.036342	0.529982	-0.001291
H	-5.439085	-0.903829	-0.893912
H	-5.439860	-0.903428	0.892494

Structure **duoc-*O*-TS**_ring-exp

C	4.506575	-0.898881	-0.240720
C	3.513736	-1.777298	0.145944
C	2.150452	-1.426301	0.066973
C	1.793973	-0.136413	-0.419445
C	2.830396	0.758773	-0.800655
C	4.153499	0.381625	-0.719980
H	5.551570	-1.184406	-0.173957

H	3.757035	-2.765786	0.523094
H	2.573971	1.755011	-1.151953
H	4.929734	1.079145	-1.020748
C	-0.238732	-2.002351	0.355598
H	-1.015596	-2.711469	0.602346
C	-0.544001	-0.732427	-0.120039
C	0.418565	0.206371	-0.477714
C	1.124894	-2.401850	0.483386
C	-0.128739	1.481465	-0.969477
H	0.239877	1.893476	-1.908576
C	-1.823301	1.006255	-1.139596
H	-2.697877	1.628595	-0.934454
H	-1.770569	0.889159	-2.224546
N	-1.847903	-0.222329	-0.389751
C	-3.028770	-0.761254	0.064380
O	-3.121339	-1.754668	0.756109
C	-0.926391	2.326880	-0.191428
H	-1.251411	3.284969	-0.578127
H	-1.184097	2.062857	0.827914
17	0.948191	3.954466	1.261552
O	1.458279	-3.546917	0.908240
O	-4.075855	-0.044387	-0.369510
C	-5.363413	-0.519194	0.051018
H	-5.429704	-0.507048	1.139474
H	-6.080219	0.172188	-0.385329
H	-5.527770	-1.530927	-0.321444

Structure **duoc-*O*-prod**_ring-exp

C	-4.677643	0.187713	0.176690
C	-3.685515	1.146522	0.046614
C	-2.338460	0.768819	-0.018476
C	-1.964272	-0.583589	0.052965
C	-2.984701	-1.543786	0.158758
C	-4.318144	-1.163929	0.227152
H	-5.721369	0.479220	0.227978
H	-3.926587	2.202742	-0.012454
H	-2.747725	-2.601227	0.177213
H	-5.085063	-1.927167	0.312534
C	0.080700	1.409383	-0.316004
H	0.805808	2.198050	-0.457525
C	0.457935	0.109895	-0.227811
C	-0.532964	-0.964950	0.000588
C	-1.309348	1.823844	-0.202087
C	-0.114400	-2.225349	0.239247
H	-0.825748	-2.985499	0.544502
C	2.043894	-1.661008	-0.796978
H	3.112293	-1.846551	-0.815098
H	1.659734	-1.720607	-1.820804
N	1.803764	-0.295066	-0.300172
C	2.834604	0.518453	0.128225
O	2.702285	1.611939	0.644638
C	1.311157	-2.647076	0.092424
H	1.354925	-3.648638	-0.346156
H	1.787723	-2.712670	1.080161
O	-1.622467	3.014141	-0.279005
O	4.029889	-0.050198	-0.080356
C	5.157686	0.716561	0.361705
H	5.097374	0.889432	1.436981
H	6.028090	0.111395	0.118852
H	5.193377	1.670906	-0.165272

Structure **duoc-A-react**

C	-2.749690	-0.957017	-0.008359
H	-2.360532	-1.859163	0.480853
C	-4.173335	-1.132734	-0.479385
H	-4.806448	-1.373024	0.376578
H	-4.528641	-0.200225	-0.927007
H	-4.231977	-1.936239	-1.216715
O	-2.681356	0.123323	0.864352
O	-1.962640	-0.701161	-1.192481
H	-2.775664	1.340817	-1.394777
H	-4.339743	2.117489	-0.007025
O	-3.559532	-2.261206	2.718387
C	-1.744668	0.060180	1.934919
H	-1.017049	0.869791	1.829237
H	-2.291582	0.173897	2.875390
H	-1.216447	-0.898618	1.944915
O	-4.955992	2.111448	0.786863
H	-5.807906	2.434941	0.473548
H	-4.943367	0.699637	1.863359
O	-5.129950	0.013010	2.543961
H	-4.077669	-1.431626	2.662575
H	-5.388506	0.488646	3.340715
H	-4.211817	-2.968470	2.764017
N	-3.336318	2.191960	-1.410045
H	-2.687763	2.972039	-1.336690
C	-4.092448	2.282867	-2.665682
H	-4.780108	1.435664	-2.731361
H	-4.689381	3.197605	-2.662433
H	-3.459752	2.283212	-3.560153
C	-0.601774	-0.706216	-0.990452
C	0.090903	-1.953367	-1.053730
C	0.043970	0.485777	-0.762610
C	1.502414	-1.964572	-0.838606
C	1.440991	0.445610	-0.559173
H	-0.506974	1.415107	-0.719711
C	2.152556	-0.734465	-0.584729
C	-0.581893	-3.173219	-1.329701
C	2.193426	-3.206967	-0.901347
C	1.514531	-4.372237	-1.161882
C	0.113487	-4.357314	-1.379618
H	-1.651729	-3.152742	-1.512313
H	-0.406678	-5.285122	-1.593685
H	2.053994	-5.312802	-1.210016
H	3.269459	-3.225552	-0.752177
C	3.599286	-0.472977	-0.225332
H	4.304905	-0.989664	-0.882038
C	3.710595	1.055682	-0.392969
H	4.313943	1.524929	0.384920
H	4.124041	1.320298	-1.370788
N	2.311611	1.521435	-0.310952
C	3.830057	-0.918462	1.218327
H	3.607023	-1.976690	1.352257
H	3.227361	-0.328267	1.911391
C	1.945114	2.825840	-0.182724
O	0.795302	3.236042	-0.157649

O	3.025861	3.615393	-0.078334
C	2.750953	5.011583	0.090780
H	2.177677	5.175405	1.004464
H	3.724759	5.491062	0.159988
H	2.195940	5.393517	-0.767229
Cl	5.554894	-0.693669	1.707044

Structure **duoc-A-TS**

C	-2.974592	-0.910562	0.368557
H	-2.467191	-1.837317	0.106822
C	-4.289630	-0.558118	-0.209456
H	-4.936751	-1.434877	-0.223830
H	-4.745719	0.264763	0.343407
H	-4.094183	-0.254185	-1.242477
O	-2.303858	0.011768	0.939785
O	-1.923729	-0.366214	-2.043853
H	-2.491205	1.427519	-1.287728
H	-3.501432	2.327328	0.561913
O	-3.603823	-1.867339	2.135961
C	-1.038428	-0.321136	1.560640
H	-0.411607	0.561786	1.459738
H	-1.249234	-0.544100	2.606694
H	-0.583632	-1.176029	1.055305
O	-3.959698	2.397641	1.469284
H	-4.634469	3.081475	1.391455
H	-4.274649	1.140454	2.513204
O	-4.504779	0.387085	3.119039
H	-4.010658	-1.081863	2.600462
H	-4.194001	0.631430	3.998295
H	-4.284643	-2.546951	2.048675
N	-2.850110	2.335330	-0.969074
H	-2.057094	2.973777	-0.973618
C	-3.874813	2.813117	-1.908118
H	-4.731874	2.134601	-1.877268
H	-4.221849	3.802394	-1.599909
H	-3.521476	2.875500	-2.943330
C	-0.698416	-0.515058	-1.684531
C	-0.149565	-1.861301	-1.487955
C	0.149806	0.583025	-1.420749
C	1.165320	-2.050867	-0.963460
C	1.428717	0.352788	-0.898202
H	-0.209237	1.588659	-1.586093
C	1.943833	-0.910212	-0.657434
C	-0.940613	-2.993200	-1.790049
C	1.629797	-3.382252	-0.765241
C	0.833611	-4.463612	-1.069291
C	-0.468374	-4.273801	-1.588963
H	-1.936282	-2.821668	-2.190405
H	-1.087240	-5.132625	-1.828740
H	1.209247	-5.470449	-0.911679
H	2.631600	-3.540830	-0.373359
C	3.288224	-0.811960	0.023146
H	4.040906	-1.494294	-0.384801
C	3.680567	0.653438	-0.242436
H	4.175110	1.123938	0.608320
H	4.330147	0.736948	-1.119563
N	2.392268	1.318469	-0.522116
C	3.103144	-1.098866	1.511717
H	2.668989	-2.084619	1.678699
H	2.476370	-0.337838	1.981505
C	2.220571	2.665164	-0.525136
O	1.175675	3.253670	-0.762140
O	3.372127	3.294908	-0.219998
C	3.292130	4.722894	-0.163318
H	2.576522	5.032973	0.599968
H	4.293999	5.058836	0.095546
H	2.992973	5.125073	-1.132503
Cl	4.684142	-1.084438	2.401216

Structure **duoc-A-prod**

C	-2.827843	-0.990746	1.076088
H	-2.070165	-1.755986	0.841839
C	-4.048989	-1.124716	0.196310
H	-4.457195	-2.136971	0.262087
H	-4.811004	-0.410769	0.523318
H	-3.768258	-0.915030	-0.839108
O	-2.290118	0.277846	0.896627
O	-1.991519	0.018726	-2.315662
H	-2.238028	0.994755	-2.008711
H	-2.304294	2.451674	-0.486401
O	-3.162465	-1.097857	2.465675
C	-1.031909	0.452609	1.534796
H	-0.622287	1.396210	1.173492
H	-1.146881	0.492991	2.623423
H	-0.354133	-0.369212	1.264563
O	-2.443335	3.201849	1.465548
H	-2.920406	4.036773	1.527576
H	-2.984145	2.554794	1.961829
O	-4.086379	1.506547	2.995595
H	-3.811447	0.575088	2.898743
H	-4.127593	1.682518	3.942263
H	-3.579737	-1.951457	2.640579
N	-2.668360	2.350732	-1.437573
H	-2.319679	3.141772	-1.974598
C	-4.135992	2.402700	-1.362400
H	-4.476895	1.577013	-0.734695
H	-4.505996	3.340713	-0.935617
H	-4.561409	2.276642	-2.360578
C	-0.800780	-0.317078	-1.806363
C	-0.561649	-1.706992	-1.529881
C	0.176991	0.634149	-1.555856
C	0.677987	-2.107497	-0.946579
C	1.388529	0.205348	-0.980165
H	0.004879	1.677741	-1.779477
C	1.648927	-1.115150	-0.677121
C	-1.547715	-2.686310	-1.811116
C	0.877998	-3.486900	-0.657206
C	-0.100461	-4.412680	-0.932348
C	-1.327587	-4.012070	-1.516928
H	-2.481431	-2.363627	-2.259701
H	-2.089676	-4.753909	-1.732810
H	0.069268	-5.460728	-0.705233
H	1.820976	-3.806285	-0.221275
C	2.977700	-1.234262	0.035002
H	3.591524	-2.065987	-0.323327
C	3.641019	0.117828	-0.288974

H	4.204207	0.528304	0.549731
H	4.303245	0.037469	-1.156693
N	2.501002	0.994881	-0.623483
C	2.716638	-1.403005	1.530841
H	2.100667	-2.278618	1.735872
H	2.237654	-0.513876	1.946579
C	2.579979	2.349524	-0.708234
O	1.661380	3.097281	-1.006710
O	3.820223	2.774835	-0.409234
C	4.003751	4.195027	-0.438133
H	3.345178	4.676605	0.286388
H	5.046096	4.357090	-0.172587
H	3.798730	4.582739	-1.437120
Cl	4.257523	-1.635894	2.451289

Structure **duoc-B-react**

C	-2.835188	-0.110788	-0.611939
O	-2.639314	0.882254	0.345823
H	-2.062227	1.925852	-1.955437
H	-3.389015	3.110856	-0.408978
C	-2.043082	0.453037	1.567308
H	-1.035838	0.055419	1.401092
H	-1.983072	1.334803	2.206311
H	-2.668743	-0.306516	2.045719
O	-4.031379	3.373651	0.278425
H	-4.598476	4.039337	-0.126114
H	-4.624095	1.861487	1.230903
O	-5.091307	1.255443	1.839163
H	-4.901237	-0.776985	1.597276
H	-5.879754	1.731959	2.121388
H	-2.483391	3.202197	-2.730206
O	-2.224955	2.880379	-1.858650
N	-4.698663	-1.777016	1.629958
O	-1.856272	0.039436	-1.667799
C	-0.560526	-0.273930	-1.309416
C	-0.161480	-1.643199	-1.347427
C	0.295530	0.741873	-0.955688
C	1.171762	-1.974981	-0.959558
C	1.613776	0.384395	-0.593966
H	-0.040720	1.769812	-0.937312
C	2.040549	-0.926312	-0.578604
C	-1.044184	-2.675505	-1.763139
C	1.572668	-3.340065	-0.989504
C	0.693583	-4.318237	-1.385200
C	-0.627159	-3.984454	-1.778935
H	-1.305843	-4.767432	-2.100589
H	1.012721	-5.355342	-1.406325
H	2.588268	-3.602677	-0.706849
C	3.452716	-1.004169	-0.040620
H	4.099927	-1.660712	-0.629069
C	3.923941	0.460739	-0.134679
H	4.512953	0.772882	0.728507
H	4.507580	0.634528	-1.043655
N	2.666245	1.231888	-0.206029
C	3.397467	-1.506870	1.401617
H	2.925048	-2.486692	1.467433
H	2.864803	-0.801560	2.042464
C	2.597435	2.587959	-0.104926
O	1.580574	3.253069	-0.220817
O	3.808976	3.107066	0.150133
C	3.845395	4.531525	0.303044
H	3.213888	4.837765	1.138339
H	4.886775	4.773620	0.502871
H	3.507849	5.018027	-0.613194
Cl	5.055380	-1.690972	2.095214
H	-2.048318	-2.413398	-2.081426
H	-5.439553	-2.242758	1.112816
C	-4.713999	-2.224078	3.025210
H	-4.512279	-3.297503	3.071259
H	-3.915172	-1.716674	3.574625
H	-5.658088	-2.032270	3.553379
C	-4.184971	0.057927	-1.267784
H	-4.965560	-0.042312	-0.512109
H	-4.255796	1.055249	-1.711778
H	-4.326359	-0.695776	-2.045488
H	-2.716800	-1.102358	-0.156988

Structure **duoc-B-TS**

C	-3.229041	-0.155391	0.097906
H	-2.531822	-0.947330	0.343776
C	-4.304147	-0.282837	-0.916160
H	-4.330453	-1.294315	-1.320774
H	-5.260200	-0.032288	-0.450357
H	-4.104923	0.429376	-1.720154
O	-3.203102	0.940086	0.767760
H	-2.063506	1.883101	-1.325543
H	-4.017920	2.955888	-0.543614
C	-2.143856	1.097228	1.730771
H	-1.201557	1.215410	1.194020
H	-2.383504	1.993053	2.298074
H	-2.118453	0.221417	2.382967
O	-4.914236	3.098476	-0.162019
H	-5.456863	3.461844	-0.870201
H	-5.608154	1.855268	0.953543
O	-6.044285	1.203389	1.541518
H	-5.129040	-0.600531	1.972488
H	-6.937629	1.533366	1.686756
H	-2.239303	3.268798	-2.071147
O	-2.399937	2.826770	-1.230198
N	-4.511014	-1.385434	1.760910
H	-3.999992	-1.617158	2.608342
C	-5.276611	-2.545659	1.300168
H	-5.882159	-2.256719	0.437434
H	-4.589544	-3.333727	0.980407
H	-5.948448	-2.959767	2.061462
O	-1.689189	0.336700	-1.376123
C	-0.456513	-0.043695	-1.134614
C	-0.132689	-1.458031	-1.162308
C	0.554463	0.880450	-0.847052
C	1.190321	-1.903105	-0.859611
C	1.849151	0.407220	-0.571266
H	0.328448	1.938496	-0.833468
C	2.176448	-0.935934	-0.561583
C	-1.119766	-2.414942	-1.504161
C	1.460724	-3.300921	-0.886630
C	0.475026	-4.205083	-1.206754

C	-0.831451	-3.761415	-1.525178
H	-1.597469	-4.482125	-1.792749
H	0.702167	-5.266745	-1.225758
H	2.465193	-3.650997	-0.662601
C	3.607927	-1.126124	-0.116708
H	4.161394	-1.848594	-0.724565
C	4.191205	0.289446	-0.283527
H	4.876598	0.565181	0.518858
H	4.708650	0.395310	-1.242227
N	3.002310	1.165546	-0.269816
C	3.611907	-1.585889	1.339830
H	3.063174	-2.518744	1.467476
H	3.188859	-0.820491	1.993400
C	3.065992	2.519014	-0.166954
O	2.112922	3.282483	-0.197306
O	4.340104	2.926056	-0.014888
C	4.517307	4.338820	0.135680
H	3.985317	4.695567	1.019063
H	5.589048	4.485133	0.251470
H	4.153577	4.862758	-0.749551
Cl	5.293683	-1.901465	1.934965
H	-2.109743	-2.056907	-1.769946

Structure **duoc-B-prod**

C	-2.870776	-0.346342	0.568242
H	-2.266489	-1.196600	0.222646
C	-3.868029	0.098211	-0.475788
H	-4.439197	-0.746789	-0.866130
H	-4.547127	0.848249	-0.059426
H	-3.293252	0.544947	-1.291938
O	-2.077996	0.732027	0.926223
H	-1.798180	2.412312	-1.688523
H	-3.192829	3.211169	0.121208
C	-0.840406	0.393036	1.544432
H	-0.339447	-0.403025	0.981293
H	-0.221213	1.290069	1.519415
H	-0.980543	0.085345	2.588179
O	-3.857743	3.366927	0.840326
H	-4.536119	3.937716	0.462882
H	-4.364576	2.211268	1.941695
O	-4.663819	1.532944	2.600621
H	-4.065215	0.036880	2.221477
H	-5.618905	1.630211	2.690582
H	-2.110799	3.973873	-1.749596
O	-2.067002	3.230083	-1.139292
N	-3.586734	-0.818791	1.824516
H	-2.884007	-1.115934	2.505031
C	-4.557559	-1.924946	1.618633
H	-5.425766	-1.537249	1.089679
H	-4.081959	-2.715554	1.037412
H	-4.859032	-2.306607	2.592174
O	-1.441198	1.164805	-2.494557
C	-0.466663	0.494771	-1.969157
C	-0.551017	-0.960986	-1.889006
C	0.688374	1.121080	-1.460684
C	0.487312	-1.723496	-1.271168
C	1.694409	0.335742	-0.881148
H	0.775504	2.197576	-1.511903
C	1.622186	-1.043655	-0.773335
C	-1.680678	-1.632128	-2.412568
C	0.326679	-3.136062	-1.177817
C	-0.797106	-3.755631	-1.675624
C	-1.814507	-3.000178	-2.307128
H	-2.691123	-3.499767	-2.707514
H	-0.900123	-4.833605	-1.591780
H	1.110078	-3.728596	-0.711428
C	2.798707	-1.574689	0.011072
H	3.246279	-2.475085	-0.421809
C	3.782941	-0.392843	-0.046464
H	4.334430	-0.250787	0.883732
H	4.497556	-0.512008	-0.867248
N	2.916992	0.772442	-0.316180
C	2.347727	-1.873573	1.439594
H	1.523682	-2.586951	1.461270
H	2.053893	-0.957437	1.956883
C	3.331174	2.060103	-0.196040
O	2.663450	3.053246	-0.442202
O	4.602339	2.113965	0.248517
C	5.126704	3.431630	0.442177
H	4.536355	3.969735	1.185690
H	6.145377	3.288608	0.796390
H	5.123565	3.983506	-0.499070
Cl	3.681393	-2.610112	2.423284
H	-2.447399	-1.031367	-2.891897

Structure **duoc-B-TS**_cycpr

C	-2.986504	-0.302907	0.469081
H	-2.396265	-1.173408	0.150429
C	-3.962623	0.140394	-0.595894
H	-4.543782	-0.700532	-0.979603
H	-4.634117	0.908651	-0.200983
H	-3.371047	0.564375	-1.411260
O	-2.172189	0.767116	0.805516
H	-1.686168	2.471107	-1.545453
H	-3.206218	3.289321	0.100611
C	-0.980373	0.422489	1.504484
H	-0.489041	-0.430578	1.020735
H	-0.323540	1.290994	1.453435
H	-1.180546	0.189448	2.557226
O	-3.952143	3.454001	0.727022
H	-4.629912	3.920231	0.225028
H	-4.481331	2.306718	1.825047
O	-4.783492	1.624454	2.478212
H	-4.196240	0.136888	2.102320
H	-5.737397	1.727445	2.574579
H	-1.883785	4.024621	-1.648993
O	-1.907706	3.291129	-1.024556
N	-3.725731	-0.734122	1.724778
H	-3.038370	-1.032192	2.420461
C	-4.713487	-1.827387	1.527766
H	-5.564947	-1.437160	0.974121
H	-4.242500	-2.641113	0.975608
H	-5.038896	-2.178540	2.505070
O	-1.351067	1.100370	-2.392470
C	-0.397479	0.431402	-1.888838
C	-0.485785	-1.038613	-1.837228

C	0.777490	1.061642	-1.382070
C	0.538470	-1.811496	-1.230231
C	1.771597	0.280823	-0.821636
H	0.873456	2.135144	-1.463031
C	1.665652	-1.122786	-0.683828
C	-1.605731	-1.687541	-2.394813
C	0.393584	-3.219284	-1.184542
C	-0.721780	-3.828844	-1.723898
C	-1.732738	-3.062121	-2.338763
H	-2.600900	-3.551677	-2.767749
H	-0.815616	-4.909678	-1.680728
H	1.171557	-3.823669	-0.725023
C	2.921604	-1.650280	-0.026336
H	3.299566	-2.619814	-0.344836
C	3.905834	-0.490521	-0.117892
H	4.481098	-0.371107	0.800775
H	4.585762	-0.606905	-0.966819
N	3.026404	0.679840	-0.336604
C	2.105345	-1.624926	1.185048
H	1.373221	-2.404366	1.351788
H	1.971799	-0.680620	1.699543
C	3.464394	1.972652	-0.239399
O	2.786168	2.963645	-0.441347
O	4.756595	2.005812	0.117111
C	5.316335	3.316700	0.270654
H	4.780275	3.866437	1.045612
H	6.351594	3.155806	0.562357
H	5.263570	3.861169	-0.673162
Cl	3.442345	-2.425968	2.947333
H	-2.368016	-1.074154	-2.864883

Structure **duoc-B-prod**_cycpr

C	-3.234164	-0.617501	0.607955
H	-2.771857	-1.571866	0.316166
C	-4.165221	-0.080307	-0.454797
H	-4.832984	-0.857573	-0.830395
H	-4.752000	0.754255	-0.059268
H	-3.547297	0.286851	-1.276910
O	-2.272970	0.342676	0.886391
H	-1.988289	2.543787	-1.456642
H	-3.673407	3.325873	0.027916
C	-1.181444	-0.100106	1.687418
H	-0.847208	-1.093676	1.363957
H	-0.375404	0.619340	1.541225
H	-1.443720	-0.122842	2.751150
O	-4.466122	3.365940	0.610563
H	-5.225548	3.500912	0.032508
H	-4.673986	2.252879	1.882093
O	-4.788071	1.589819	2.606043
H	-4.343513	0.042585	2.241616
H	-5.658810	1.739825	2.992226
H	-2.168228	4.087137	-1.572288
O	-2.247190	3.348536	-0.958079
N	-4.003808	-0.895447	1.886259
H	-3.356551	-1.269514	2.583950
C	-5.144623	-1.839860	1.740717
H	-5.936654	-1.351253	1.177085
H	-4.804657	-2.736211	1.221648
H	-5.504466	-2.097311	2.734937
O	-1.563467	1.016226	-2.231954
C	-0.609321	0.392710	-1.736049
C	-0.643711	-1.096141	-1.696219
C	0.574288	1.082492	-1.264033
C	0.388457	-1.824863	-1.081061
C	1.591206	0.362476	-0.721291
H	0.650049	2.145602	-1.444073
C	1.485229	-1.079128	-0.435115
C	-1.716758	-1.772630	-2.293409
C	0.317044	-3.222523	-1.050378
C	-0.757186	-3.882391	-1.635400
C	-1.778239	-3.158413	-2.262503
H	-2.610470	-3.678560	-2.724705
H	-0.797421	-4.966596	-1.610771
H	1.107125	-3.797446	-0.575057
C	2.893290	-1.562473	-0.115876
H	3.233198	-2.530357	-0.465168
C	3.812987	-0.366535	-0.254371
H	4.447447	-0.242228	0.624854
H	4.436169	-0.430811	-1.150608
N	2.872573	0.771652	-0.388580
C	1.917915	-1.456712	0.990353
H	1.468025	-2.368347	1.368667
H	2.033686	-0.649920	1.709581
C	3.293581	2.085019	-0.383451
O	2.568591	3.046441	-0.543588
O	4.609854	2.150799	-0.173323
C	5.159450	3.476297	-0.131460
H	4.702693	4.044679	0.679661
H	6.223454	3.340622	0.046080
H	4.988447	3.981643	-1.082759
Cl	5.793597	-1.868519	2.506786
H	-2.491995	-1.190059	-2.780388

Structure **duoc-B-TS**_prot

C	-2.922671	-0.398865	0.649836
H	-2.319597	-1.286324	0.393115
C	-3.958868	-0.127264	-0.421867
H	-4.501785	-1.037477	-0.687098
H	-4.672894	0.630195	-0.082725
H	-3.429893	0.245503	-1.302233
O	-2.092769	0.728960	0.735846
H	-1.875459	2.112635	-1.733635
H	-3.099738	3.030347	-0.210630
C	-0.896162	0.519497	1.466528
H	-0.386313	-0.387821	1.113921
H	-0.252301	1.379734	1.278155
H	-1.083877	0.445491	2.545676
O	-3.863274	3.203624	0.535813
H	-4.697700	3.398076	0.090753
H	-4.023784	2.465167	1.475534
O	-4.210078	1.793568	2.446506
H	-3.979963	0.721871	2.303953
H	-5.102020	1.909790	2.799416
H	-2.209920	3.730882	-1.806581
O	-2.142050	2.998179	-1.183472
N	-3.561267	-0.598003	1.977155

H	-2.840585	-0.883890	2.639143
C	-4.624222	-1.617773	1.996940
H	-5.506903	-1.237750	1.480973
H	-4.300372	-2.547854	1.516807
H	-4.889103	-1.825627	3.033805
O	-1.547496	0.983288	-2.450669
C	-0.524965	0.356993	-1.939092
C	-0.557285	-1.093600	-1.831165
C	0.613270	1.039201	-1.483444
C	0.518954	-1.802006	-1.214823
C	1.656808	0.307706	-0.896026
H	0.657715	2.116752	-1.561442
C	1.633922	-1.067776	-0.749739
C	-1.667578	-1.819537	-2.323445
C	0.419022	-3.217542	-1.093463
C	-0.683734	-3.891819	-1.565824
C	-1.740234	-3.189706	-2.194681
H	-2.600369	-3.732455	-2.574102
H	-0.740433	-4.971594	-1.463424
H	1.232816	-3.768138	-0.627788
C	2.838782	-1.535303	0.032318
H	3.313160	-2.430089	-0.382995
C	3.779094	-0.320645	-0.067713
H	4.333264	-0.132906	0.852693
H	4.490134	-0.436695	-0.891978
N	2.868869	0.804582	-0.360666
C	2.412075	-1.812769	1.472353
H	1.618937	-2.559051	1.520388
H	2.083325	-0.895779	1.966503
C	3.233034	2.109820	-0.274343
O	2.523348	3.069657	-0.535213
O	4.505763	2.223446	0.153512
C	4.980857	3.564431	0.311272
H	4.379856	4.095496	1.051285
H	6.008915	3.468745	0.654118
H	4.943739	4.094859	-0.641520
Cl	3.783269	-2.465924	2.463321
H	-2.465774	-1.261460	-2.802277

Structure **duoc-B-prod**_prot

C	-1.823825	-0.333985	1.163584
H	-0.795067	-0.609562	0.855991
C	-2.813421	-1.387233	0.704719
H	-2.479413	-2.392343	0.973452
H	-3.793547	-1.198582	1.155757
H	-2.903992	-1.332812	-0.382016
O	-2.180191	0.868613	0.524182
H	-2.413754	0.449777	-2.434251
H	-3.470825	2.026569	-1.210347
C	-1.334753	1.966819	0.815838
H	-0.278988	1.659397	0.826660
H	-1.475521	2.700842	0.020960
H	-1.593582	2.424349	1.779382
O	-4.441458	2.496249	0.224903
H	-5.344731	2.830589	0.233538
H	-4.319067	1.989544	1.056374
O	-4.229873	1.298411	2.738315
H	-3.412748	0.727131	2.779188
H	-4.965742	0.749802	3.032414
H	-3.577155	2.373198	-2.724226
O	-3.002866	1.955252	-2.071965
N	-1.882426	-0.148411	2.621716
H	-1.142491	0.499161	2.888334
C	-1.674482	-1.388868	3.377685
H	-2.563515	-2.019219	3.313819
H	-0.810460	-1.963419	3.013129
H	-1.508690	-1.138260	4.426362
O	-2.071874	-0.472680	-2.580539
C	-0.848924	-0.605017	-2.027296
C	-0.412014	-1.937989	-1.736762
C	-0.063050	0.494100	-1.739833
C	0.835417	-2.130305	-1.071244
C	1.166440	0.269017	-1.089828
H	-0.415579	1.493752	-1.955277
C	1.608232	-0.991982	-0.744873
C	-1.211665	-3.063909	-2.060948
C	1.239875	-3.458312	-0.756207
C	0.446157	-4.530892	-1.085472
C	-0.793389	-4.334992	-1.744622
H	-1.410615	-5.191299	-1.996236
H	0.770545	-5.537655	-0.840915
H	2.192184	-3.618512	-0.257740
C	2.858577	-0.889714	0.098792
H	3.634151	-1.611362	-0.173739
C	3.325057	0.550684	-0.181563
H	3.719639	1.050906	0.703690
H	4.082836	0.576338	-0.970770
N	2.101725	1.230326	-0.656180
C	2.461967	-1.096935	1.560527
H	1.990547	-2.067917	1.713050
H	1.786680	-0.307099	1.899140
C	2.022702	2.572668	-0.872685
O	1.065133	3.159005	-1.350944
O	3.151686	3.184261	-0.475050
C	3.163329	4.608313	-0.630347
H	2.362484	5.059276	-0.042342
H	4.134803	4.931063	-0.262524
H	3.044283	4.874885	-1.681556
Cl	3.902711	-1.051989	2.653693
H	-2.161667	-2.899825	-2.558855

Constant pH Molecular Dynamics (CpHMD) simulations

The pK_a of titratable residues was calculated using the method implemented by MacCammon[9] in the Amber 20 package supplemented with AmberTools 20[10] for unligated Fab and all possible diastereomers of the conjugate Fab-**2**. For the generation of the topology and input coordinate files, the specifically developed *leaprc.constph* file containing all the necessary variables for a CpHMD simulation was used in combination with the leap utility. The generalized Born implicit solvent method (*igb* = 2)[11,12] was used. The underlying force field was *ff10* (equivalent to *ff99SB* for proteins) and the atomic radii (*PBRadii*) were set to mbondi2.[13] The salt concentration was set to 0.1 M. The *cpinutil.py* program was used to generate the constant pH input file (*cpin*) containing the definition of the target titratable residues (Lys-207 as the potentially reactive residue and Lys-350 as a solvent-exposed reference). An initial geometry optimization (1000 steepest-descent steps) in which the positions of the protein backbone atoms (CA, C, O and N) were restrained using a 10 kcal/mol·A^2 restraint force constant, was performed with no change in the protonation states (*icnstph* = 1, *ntcnstph* = 100000) and no cut-off for Lennard-Jones and electrostatic interactions (*cut* = 1000). The systems were then gently heated with no change in the protonation state by linearly incrementing the temperature from 10 to 300 K using the *nmropt* = 1 option. Harmonic restraints of 2 kcal/mol·A^2 were applied to the protein backbone, and temperature was controlled and equalized through Langevin dynamics (*ntt* = 3) with a collision frequency of γ = 5 ps^{-1} (*gamma_ln* = 5) and a time constant for heat bath coupling of τ_p = 2 ps (*tautp* = 2.0). A random seed (*ig* = -1) was used to initialize velocities to avoid synchronization artifacts.[14,15] No constant pressure periodic boundary conditions were used (*ntp* = 0) and the particle-mesh-Ewald method[16] to model long-range electrostatic effects was turned off (*ntb* = 0) with no cut-off for Lennard-Jones and electrostatic interactions. The SHAKE algorithm was used (*ntc* = 2, *ntf* = 2) with a relative geometrical tolerance for coordinate resetting of 1E-6 Å (*tol* = 0.000001), such that the angle between the hydrogen atoms is kept fixed. The time step was kept at 2 fs (*dt* = 0.002) during the 2 ns heating stage. Each system was then equilibrated for 2 ns with a 2 fs timestep at a constant temperature of 300 K, using Langevin dynamics under the same conditions described above. At this point constant pH in implicit solvent is turned on (*icnstph* = 1) and changing protonation states starting from physiological pH (*solvph* = 7.5) is attempted every 5 steps (*ntcnstph* = 5) depending on the number of the titratable residues of the target protein. 100 independent replicas of production trajectories were then run under the same simulation conditions for additional 1 ns to facilitate proper conformational sampling, in a range spanning pH = 0 to pH 14. After exhaustive benchmarking, we observed much more converged results by averaging over multiple short production replicas (100 x 1 ns), than by running longer single production trajectories (1 x 100 ns). The program *cphstats*

was used to analyze the results obtained from the CpHMD simulations. From these data, the pK_a values were computed using the Hill equation:

$$pK_a(Lys) = pH - n \log \frac{[LysNH_2]}{[LysNH_3^+]}$$

where $[LysNH_2]$ and $[LysNH_3^+]$ are the concentrations of the neutral and protonated forms of lysine, respectively, and *n* is the Hill coefficient.

Knowing that the fractions of each deprotonated residues (f_d) are:

$$f_d(Lys) = \frac{[LysNH_2]}{[LysNH_3^+] + [LysNH_2]}$$

Hill equation can be rearranged to:

$$f_d = \frac{1}{1 + 10^{n(pK_a - pH)}}$$

The calculated pK_a values and Hill coefficients for each residue were derived by fitting the protonated fraction ($1 - f_d$) at each considered pH using a non-linear, least-squares algorithm as implemented in the Python library SciPy (http://scipy.org). The calculated values and CpHMD simulations parameters are summarized in Supporting Tables S3-S7. Note that different replicas of identical CpHMD simulations gave different results due to deficient conformational sampling. Thus, reported values are intended to reflect global trends more than exact numbers and must not be over interpreted. All graphical representations of proteins were performed using the PyMol software (http://www.pymol.org).

Alternatively, the deprotonated (f_d) and protonated ($1 - f_d$) fractions of each residue at a given pH can be averaged over the different replicas to mitigate the effect of outliers and significantly reduce the asymptotic standard error in the non-linear fitting (σ) (Figures S24-S28).

Table S3. Individual pK_a values and Hill coefficients (n) calculated for Lys-207 and Lys-350 in unligated Fab through 100-independent 1 ns CpHMD simulations (σ = asymptotic standard error).

# replica	Lys-207				Lys-350			
	pK_a		n		pK_a		n	
	value	σ	value	σ	value	σ	value	σ
1	8.37	0.06	-0.73	0.07	9.70	0.03	-0.77	0.04
2	8.02	0.02	-1.08	0.06	8.87	0.12	-0.89	0.20
3	8.03	0.03	-1.07	0.08	9.58	0.05	-0.79	0.06
4	8.13	0.03	-0.89	0.06	9.58	0.02	-1.12	0.04
5	8.30	0.01	-0.88	0.01	9.42	0.02	-1.18	0.04
6	8.57	0.04	-1.53	0.13	9.32	0.03	-1.28	0.08
7	8.23	0.01	-0.82	0.02	9.03	0.02	-0.74	0.03
8	8.15	0.02	-1.06	0.06	9.39	0.01	-0.89	0.01
9	8.23	0.07	-1.38	0.29	9.39	0.05	-0.85	0.08
10	8.43	0.02	-1.94	0.07	9.83	0.03	-0.81	0.03

11	7.78	0.07	-1.25	0.22	9.38	0.04	-1.36	0.13
12	8.35	0.00	-0.96	0.01	9.20	0.01	-1.07	0.03
13	8.27	0.02	-1.17	0.06	9.45	0.02	-1.23	0.05
14	8.17	0.03	-0.98	0.06	9.44	0.02	-0.76	0.03
15	8.13	0.01	-1.48	0.06	9.15	0.04	-0.87	0.06
16	8.17	0.01	-1.00	0.03	8.84	0.10	-0.57	0.06
17	7.69	0.18	-0.54	0.11	9.20	0.01	-1.04	0.03
18	8.51	0.06	-1.90	0.22	9.17	0.05	-0.79	0.06
19	8.21	0.04	-1.14	0.10	9.50	0.02	-0.97	0.03
20	7.72	0.02	-1.13	0.06	9.02	0.01	-1.32	0.08
21	8.13	0.02	-1.08	0.06	9.23	0.05	-0.77	0.06
22	7.82	0.02	-1.87	0.20	9.24	0.02	-0.72	0.02
23	8.47	0.01	-1.31	0.02	9.66	0.02	-1.44	0.05
24	7.71	0.06	-1.23	0.16	9.24	0.01	-1.20	0.03
25	7.38	0.09	-1.06	0.19	9.06	0.02	-1.11	0.07
26	8.57	0.06	-1.35	0.15	9.42	0.01	-1.01	0.01
27	8.41	0.02	-0.79	0.02	9.13	0.02	-0.90	0.04
28	8.20	0.04	-0.82	0.05	10.06	0.06	-1.05	0.17
29	8.68	0.02	-1.25	0.06	9.49	0.02	-0.74	0.03
30	8.42	0.01	-0.88	0.02	9.48	0.01	-1.30	0.03
31	8.30	0.01	-1.67	0.05	9.22	0.02	-0.84	0.02
32	7.65	0.02	-1.28	0.06	9.49	0.03	-0.81	0.04
33	7.83	0.02	-0.94	0.04	9.46	0.03	-1.02	0.06
34	8.54	0.03	-1.88	0.10	9.43	0.02	-1.46	0.05
35	8.22	0.01	-1.29	0.05	9.72	0.02	-0.96	0.03
36	8.21	0.01	-1.78	0.09	9.40	0.02	-1.10	0.04
37	8.28	0.02	-1.90	0.15	9.45	0.07	-0.67	0.07
38	8.41	0.05	-1.35	0.13	10.10	0.09	-0.98	0.19
39	7.75	0.11	-2.15	0.89	9.38	0.07	-0.79	0.09
40	7.85	0.04	-1.01	0.09	9.13	0.01	-1.36	0.05
41	8.11	0.12	-0.84	0.17	9.50	0.01	-0.91	0.02
42	8.24	0.02	-1.42	0.07	9.67	0.00	-1.08	0.01
43	8.21	0.02	-0.88	0.03	9.63	0.00	-0.94	0.00
44	7.78	0.02	-1.72	0.15	9.82	0.03	-0.87	0.05
45	8.50	0.07	-0.65	0.06	9.75	0.03	-1.81	0.17
46	8.45	0.01	-0.92	0.02	9.41	0.02	-0.88	0.03
47	8.17	0.04	-0.82	0.06	9.47	0.04	-1.33	0.12
48	8.24	0.02	-1.03	0.04	9.60	0.01	-1.00	0.01
49	8.06	0.09	-0.77	0.11	9.49	0.04	-1.48	0.12
50	7.87	0.01	-1.00	0.02	9.42	0.02	-0.86	0.03
51	8.31	0.06	-0.72	0.07	8.73	0.05	-0.89	0.08
52	8.43	0.01	-1.19	0.03	9.50	0.02	-0.83	0.03
53	8.18	0.01	-1.36	0.06	9.42	0.00	-1.00	0.01
54	8.44	0.04	-0.73	0.04	9.44	0.02	-1.23	0.04
55	7.87	0.08	-0.93	0.14	9.20	0.01	-1.39	0.06
56	8.18	0.03	-0.87	0.06	9.26	0.01	-1.14	0.03
57	8.43	0.01	-1.74	0.05	9.62	0.02	-0.86	0.02
58	7.80	0.03	-0.81	0.03	8.94	0.01	-1.21	0.04
59	7.95	0.01	-1.33	0.08	9.16	0.02	-1.04	0.05
60	8.41	0.03	-1.18	0.07	9.57	0.07	-1.44	0.21
61	7.44	0.06	-2.19	0.26	9.30	0.03	-0.74	0.04
62	8.32	0.04	-1.33	0.13	9.41	0.01	-1.45	0.04
63	8.55	0.03	-0.92	0.04	9.54	0.01	-0.90	0.02
64	8.77	0.08	-0.73	0.08	9.67	0.02	-1.05	0.04
65	8.18	0.01	-1.53	0.05	9.46	0.01	-1.39	0.04
66	8.30	0.02	-1.40	0.06	9.59	0.01	-1.00	0.03
67	8.28	0.01	-1.08	0.01	8.83	0.06	-1.25	0.24
68	8.37	0.01	-1.09	0.02	9.83	0.05	-0.77	0.07

69	8.38	0.03	-1.64	0.12	9.63	0.01	-0.92	0.02
70	8.78	0.00	-0.92	0.01	9.33	0.03	-1.32	0.08
71	7.96	0.06	-0.91	0.11	9.40	0.02	-1.11	0.04
72	8.43	0.02	-0.79	0.02	9.57	0.01	-1.21	0.02
73	8.28	0.02	-1.73	0.09	10.10	0.04	-1.05	0.11
74	8.45	0.01	-1.68	0.04	9.69	0.02	-0.83	0.03
75	8.32	0.01	-1.08	0.03	9.49	0.00	-1.06	0.01
76	7.90	0.02	-0.94	0.04	9.22	0.02	-0.77	0.02
77	8.07	0.02	-0.89	0.03	9.43	0.01	-0.97	0.02
78	8.30	0.04	-1.72	0.19	9.17	0.05	-0.82	0.07
79	8.34	0.01	-1.46	0.02	9.63	0.01	-1.15	0.02
80	8.16	0.02	-0.94	0.04	9.38	0.01	-1.16	0.03
81	8.46	0.02	-1.52	0.05	9.28	0.03	-0.88	0.05
82	8.24	0.01	-0.94	0.02	9.43	0.02	-0.83	0.03
83	8.84	0.01	-1.34	0.06	9.71	0.02	-1.32	0.08
84	8.49	0.01	-0.97	0.02	9.65	0.02	-1.17	0.04
85	7.92	0.12	-0.72	0.12	9.28	0.03	-0.87	0.05
86	7.98	0.02	-1.26	0.07	9.00	0.06	-0.90	0.10
87	8.63	0.03	-0.80	0.04	9.02	0.01	-1.18	0.06
88	8.20	0.02	-0.97	0.03	9.41	0.02	-0.79	0.03
89	8.28	0.06	-0.90	0.09	9.23	0.07	-0.65	0.06
90	8.26	0.02	-1.21	0.06	9.30	0.02	-0.92	0.03
91	8.33	0.05	-1.26	0.13	9.41	0.01	-1.12	0.01
92	8.09	0.04	-0.96	0.08	9.69	0.02	-0.93	0.03
93	8.00	0.02	-1.29	0.10	9.29	0.03	-0.81	0.04
94	8.38	0.02	-1.11	0.03	8.80	0.06	-1.49	0.29
95	8.24	0.00	-1.18	0.01	9.63	0.06	-0.68	0.05
96	7.58	0.06	-0.64	0.05	9.55	0.01	-1.11	0.02
97	8.55	0.01	-0.90	0.01	9.35	0.07	-1.87	0.33
98	8.18	0.09	-1.28	0.34	9.33	0.02	-0.85	0.04
99	8.17	0.00	-0.86	0.01	9.03	0.01	-1.02	0.02
100	8.25	0.02	-0.87	0.03	9.41	0.03	-0.77	0.04
average	8.21	0.28	-1.17	0.36	9.40	0.26	-1.03	0.25

Table S4. Individual pK_a values and Hill coefficients (n) calculated for Lys-207 and Lys-350 in Fab-**2** (*RR* diastereomer) through 100-independent 1 ns CpHMD simulations (σ = asymptotic standard error).

**# replica**	**Lys-207**				**Lys-350**			
	**pK_a**		**n**		**pK_a**		**n**	
	**value**	**σ**	**value**	**σ**	**value**	**σ**	**value**	**σ**
1	6.67	0.28	-0.65	0.24	9.41	0.02	-1.44	0.05
2	7.35	0.02	-2.53	0.17	9.27	0.02	-1.38	0.06
3	8.61	0.02	-2.04	0.08	9.58	0.01	-0.91	0.02
4	8.92	0.06	-0.90	0.11	9.37	0.02	-0.91	0.04
5	7.63	0.03	-1.01	0.06	9.33	0.07	-0.73	0.08
6	7.47	0.02	-1.32	0.06	9.70	0.01	-1.56	0.05
7	8.28	0.06	-0.91	0.10	9.77	0.02	-0.85	0.03
8	7.13	0.01	-1.91	0.15	9.14	0.02	-1.09	0.05
9	7.19	0.07	-0.80	0.09	9.18	0.01	-1.76	0.10
10	7.38	0.06	-1.04	0.12	9.38	0.04	-1.91	0.19
11	6.80	0.03	-0.91	0.05	9.19	0.01	-1.46	0.04
12	5.92	0.43	-0.50	0.22	9.08	0.00	-1.62	0.04
13	7.10	0.06	-1.29	0.29	9.32	0.02	-1.06	0.03
14	8.46	0.00	-0.99	0.01	9.68	0.01	-1.15	0.03

15	7.17	0.01	-1.61	0.06	9.31	0.02	-0.99	0.04
16	7.64	0.04	-2.25	0.22	9.71	0.10	-0.67	0.09
17	8.44	0.17	-1.58	0.57	9.38	0.01	-0.88	0.02
18	7.61	0.24	-0.54	0.14	9.27	0.04	-1.42	0.16
19	8.35	0.03	-0.86	0.05	9.25	0.03	-0.76	0.04
20	8.19	0.63	-3.42	11.10	9.25	0.01	-0.95	0.03
21	8.65	0.03	-2.02	0.17	9.32	0.02	-0.87	0.03
22	7.72	0.01	-1.19	0.03	9.55	0.01	-0.88	0.01
23	6.97	0.14	-0.71	0.14	9.64	0.07	-1.31	0.21
24	7.67	0.05	-0.85	0.07	9.40	0.02	-1.45	0.06
25	7.70	2.37	-4.14	33.16	9.45	0.00	-1.37	0.01
26	6.80	0.11	-2.00	0.96	9.50	0.01	-1.16	0.02
27	7.87	0.05	-1.30	0.23	8.98	0.04	-0.90	0.08
28	7.38	0.04	-1.12	0.08	9.70	0.02	-1.24	0.05
29	7.70	0.26	-0.48	0.13	9.41	0.06	-0.69	0.06
30	7.50	0.02	-0.95	0.03	9.51	0.05	-1.75	0.18
31	9.11	0.31	-0.53	0.18	9.89	0.06	-0.84	0.08
32	7.42	0.02	-0.95	0.03	9.42	0.02	-0.84	0.03
33	7.22	0.10	-1.09	0.26	9.04	0.02	-1.14	0.07
34	9.16	0.24	-0.44	0.09	9.56	0.01	-1.28	0.01
35	7.44	0.16	-1.96	0.65	9.07	0.06	-0.75	0.06
36	7.96	0.03	-1.11	0.10	9.42	0.02	-1.45	0.06
37	7.65	0.44	-0.41	0.15	9.48	0.03	-1.10	0.07
38	7.49	0.09	-2.23	0.39	9.40	0.03	-0.68	0.03
39	9.92	0.27	-0.63	0.22	9.43	0.01	-0.93	0.01
40	7.96	0.05	-0.95	0.10	9.29	0.01	-1.16	0.03
41	7.66	0.14	-1.27	0.39	9.78	0.05	-1.09	0.13
42	6.61	2.80	-4.53	32.06	9.59	0.09	-0.65	0.08
43	7.47	0.01	-2.38	0.03	9.47	0.02	-1.31	0.05
44	6.61	0.52	-3.30	4.19	9.80	0.00	-1.14	0.00
45	7.34	0.03	-0.84	0.05	9.74	0.01	-1.53	0.04
46	7.70	0.59	-4.38	8.61	9.57	0.05	-1.02	0.09
47	6.32	0.20	-0.54	0.12	9.07	0.02	-1.45	0.12
48	7.25	0.11	-0.66	0.10	9.57	0.01	-0.91	0.02
49	8.09	0.04	-1.11	0.12	9.62	0.01	-1.26	0.04
50	8.16	0.12	-0.85	0.18	10.14	0.02	-1.01	0.05
51	8.61	0.02	-1.47	0.06	9.44	0.05	-0.74	0.06
52	8.03	0.02	-0.77	0.03	9.58	0.02	-1.06	0.05
53	7.64	0.35	-0.47	0.16	9.69	0.01	-1.22	0.02
54	8.42	0.10	-2.19	0.50	9.23	0.03	-1.16	0.10
55	7.42	0.47	-0.43	0.18	9.43	0.05	-1.49	0.15
56	8.43	0.21	-0.53	0.12	9.90	0.02	-1.10	0.06
57	7.75	0.02	-1.49	0.10	9.35	0.00	-0.99	0.00
58	9.29	0.82	-2.92	8.05	9.86	0.01	-1.29	0.02
59	7.70	0.10	-0.67	0.09	9.24	0.01	-1.34	0.02
60	6.60	0.02	-1.61	0.07	9.63	0.01	-1.33	0.02
61	6.77	0.03	-0.79	0.04	9.12	0.02	-1.13	0.06
62	5.82	0.28	-0.46	0.12	9.44	0.01	-1.17	0.02
63	9.25	0.03	-1.44	0.15	9.65	0.14	-0.49	0.07
64	8.22	0.06	-2.52	0.66	9.38	0.01	-0.95	0.01
65	7.52	0.03	-2.82	0.16	8.85	0.12	-0.86	0.19
66	6.55	0.21	-2.66	1.17	9.37	0.02	-1.94	0.08
67	7.71	0.06	-1.45	0.22	9.83	0.01	-1.39	0.03
68	7.77	0.04	-2.17	0.35	9.16	0.02	-1.08	0.04
69	8.24	0.41	-0.50	0.20	9.39	0.02	-1.79	0.08
70	7.27	0.02	-1.10	0.04	9.03	0.03	-0.97	0.07
71	7.45	0.01	-1.07	0.02	9.64	0.01	-1.24	0.03
72	7.46	0.05	-2.73	0.27	9.93	0.04	-0.90	0.08

73	7.81	0.05	-0.80	0.07	9.33	0.16	-0.59	0.11
74	7.62	0.04	-0.83	0.06	9.25	0.08	-0.72	0.09
75	7.86	0.03	-2.62	0.60	9.48	0.03	-0.75	0.03
76	7.62	0.00	-1.23	0.01	9.06	0.06	-0.90	0.11
77	6.92	0.20	-0.68	0.19	9.38	0.01	-1.50	0.05
78	6.40	0.11	-0.68	0.10	9.35	0.04	-0.72	0.05
79	8.37	0.15	-1.86	0.64	9.37	0.03	-1.40	0.09
80	6.70	0.16	-1.75	0.78	9.41	0.01	-1.13	0.02
81	7.61	0.01	-1.05	0.02	9.53	0.01	-1.01	0.01
82	7.02	0.00	-1.70	0.02	9.39	0.01	-1.13	0.03
83	7.50	0.28	-0.53	0.16	9.19	0.01	-1.04	0.03
84	8.34	0.02	-1.89	0.11	9.23	0.01	-1.54	0.04
85	6.40	0.27	-0.47	0.12	10.16	0.05	-1.57	0.35
86	8.17	0.03	-1.07	0.09	9.43	0.04	-0.76	0.04
87	6.76	0.03	-1.13	0.08	9.77	0.01	-1.31	0.04
88	7.86	0.03	-1.13	0.10	9.30	0.00	-0.91	0.01
89	7.83	0.20	-0.64	0.17	9.13	0.06	-0.65	0.05
90	7.62	0.01	-1.19	0.03	9.46	0.01	-1.07	0.02
91	8.08	0.39	-0.51	0.20	9.48	0.03	-0.84	0.04
92	7.65	0.33	-2.38	2.10	9.63	0.03	-0.86	0.04
93	6.97	0.07	-0.84	0.10	9.18	0.01	-1.11	0.04
94	7.51	0.00	-1.80	0.02	9.36	0.02	-1.23	0.04
95	8.27	0.10	-1.02	0.21	9.73	0.03	-1.31	0.10
96	9.21	0.09	-1.15	0.27	9.49	0.01	-1.16	0.03
97	7.37	0.25	-0.55	0.16	8.95	0.07	-0.99	0.15
98	8.28	0.38	-0.46	0.16	9.51	0.01	-1.00	0.01
99	7.91	0.02	-1.11	0.05	9.34	0.02	-1.11	0.05
100	7.96	0.10	-0.77	0.12	9.38	0.01	-1.25	0.01
average	7.66	0.74	-1.36	0.88	9.44	0.25	-1.12	0.30

Table S5. Individual pK_a values and Hill coefficients (n) calculated for Lys-207 and Lys-350 in Fab-**2** (*RS* diastereomer) through 100-independent 1 ns CpHMD simulations (σ = asymptotic standard error).

**# replica**	**Lys-207**				**Lys-350**			
	**pK_a**		**n**		**pK_a**		**n**	
	**value**	**σ**	**value**	**σ**	**value**	**σ**	**value**	**σ**
1	7.33	0.15	-2.11	0.90	9.31	0.01	-1.41	0.02
2	8.21	0.09	-1.64	0.55	9.43	0.01	-1.04	0.03
3	7.24	0.06	-0.77	0.08	9.25	0.06	-0.69	0.06
4	7.76	0.21	-2.46	2.05	9.60	0.01	-1.15	0.03
5	7.15	0.01	-1.14	0.03	9.73	0.02	-1.30	0.07
6	7.30	0.06	-2.35	0.44	9.60	0.02	-1.09	0.05
7	7.60	0.07	-1.36	0.20	9.16	0.01	-1.23	0.05
8	6.60	0.03	-0.73	0.04	9.48	0.02	-1.47	0.06
9	7.22	0.05	-3.16	0.68	9.61	0.01	-1.00	0.02
10	7.01	0.05	-1.28	0.25	9.41	0.02	-1.30	0.05
11	7.02	0.03	-1.29	0.14	9.45	0.03	-0.99	0.05
12	7.23	0.09	-0.59	0.06	9.54	0.05	-0.66	0.05
13	6.82	0.11	-0.74	0.12	9.60	0.01	-1.06	0.01
14	7.08	0.01	-1.89	0.22	9.39	0.01	-1.05	0.01
15	6.68	0.11	-1.85	0.53	9.67	0.04	-0.72	0.05
16	6.84	0.03	-2.04	0.40	9.82	0.01	-0.85	0.02
17	6.82	0.21	-0.55	0.13	9.28	0.00	-1.19	0.01
18	6.84	0.02	-1.84	0.23	9.64	0.01	-1.07	0.02

19	6.48	0.15	-2.10	0.65	9.45	0.03	-0.83	0.04
20	6.64	0.11	-2.91	0.86	9.66	0.01	-1.47	0.02
21	7.12	0.07	-0.82	0.10	9.73	0.01	-1.34	0.04
22	8.19	0.09	-0.81	0.12	9.58	0.01	-1.05	0.02
23	6.57	0.07	-0.78	0.08	9.68	0.01	-1.03	0.02
24	6.60	0.07	-3.32	0.56	9.19	0.15	-0.49	0.07
25	7.52	0.07	-2.43	0.34	9.75	0.01	-1.26	0.05
26	6.63	0.31	-2.66	2.16	9.50	0.03	-1.15	0.06
27	6.62	0.06	-0.75	0.06	9.74	0.00	-1.24	0.02
28	6.72	0.03	-0.92	0.04	9.51	0.01	-1.01	0.02
29	7.90	0.08	-3.12	2.43	9.72	0.01	-0.92	0.02
30	7.20	0.01	-1.11	0.04	9.73	0.03	-0.91	0.06
31	6.89	0.04	-1.02	0.10	9.47	0.05	-1.48	0.14
32	6.50	0.43	-3.20	2.76	9.78	0.05	-0.73	0.06
33	6.74	0.09	-1.97	0.61	9.46	0.01	-1.24	0.01
34	7.18	0.03	-1.24	0.09	9.15	0.07	-0.79	0.09
35	7.41	0.07	-1.92	0.31	9.90	0.05	-0.86	0.08
36	6.34	0.31	-0.49	0.15	9.89	0.03	-0.88	0.05
37	5.92	0.11	-0.69	0.11	9.24	0.09	-0.67	0.09
38	6.41	0.03	-2.91	0.21	9.69	0.02	-1.10	0.05
39	7.38	0.02	-2.13	0.11	9.68	0.03	-1.28	0.09
40	5.35	0.16	-2.95	1.33	9.49	0.01	-1.11	0.02
41	6.98	0.02	-1.41	0.13	9.19	0.01	-1.77	0.10
42	7.10	0.03	-1.56	0.27	9.48	0.04	-0.79	0.04
43	6.97	0.03	-1.26	0.12	9.30	0.03	-0.81	0.04
44	6.41	0.08	-0.71	0.08	9.39	0.00	-1.01	0.01
45	6.22	0.06	-1.65	0.37	9.96	0.03	-1.12	0.08
46	7.80	1.04	-3.10	16.08	9.65	0.02	-1.03	0.04
47	6.36	0.51	-3.30	4.64	9.58	0.01	-1.42	0.04
48	6.10	0.02	-1.04	0.05	9.63	0.01	-1.50	0.04
49	7.00	0.20	-0.54	0.12	9.67	0.01	-1.04	0.01
50	6.93	0.06	-1.18	0.22	9.32	0.03	-1.74	0.16
51	7.52	0.01	-1.20	0.02	9.56	0.04	-1.53	0.13
52	6.61	0.01	-0.97	0.03	9.34	0.02	-0.89	0.04
53	6.43	0.11	-0.59	0.08	9.43	0.00	-1.38	0.01
54	7.95	0.07	-0.83	0.10	9.51	0.02	-1.12	0.04
55	7.26	0.09	-0.64	0.07	9.15	0.04	-0.79	0.05
56	6.89	0.03	-1.32	0.15	9.71	0.01	-1.18	0.03
57	8.10	0.07	-0.73	0.07	9.83	0.06	-0.75	0.07
58	6.62	0.04	-0.75	0.04	9.53	0.02	-1.50	0.07
59	6.40	0.17	-0.59	0.12	9.71	0.01	-1.02	0.02
60	6.65	0.02	-0.93	0.04	9.32	0.04	-0.73	0.05
61	6.86	0.02	-1.11	0.04	9.59	0.03	-1.22	0.07
62	6.85	0.07	-0.86	0.10	9.74	0.02	-1.00	0.04
63	7.20	0.17	-0.60	0.13	9.55	0.01	-0.94	0.02
64	7.33	0.10	-2.40	0.69	9.76	0.02	-1.03	0.04
65	6.66	0.15	-0.58	0.10	8.69	0.05	-1.99	0.26
66	6.78	0.14	-0.65	0.12	9.52	0.07	-0.62	0.06
67	7.31	0.01	-1.21	0.02	9.49	0.01	-0.92	0.01
68	7.75	0.02	-0.98	0.04	9.44	0.02	-1.08	0.04
69	6.63	0.01	-1.48	0.02	9.44	0.02	-1.17	0.04
70	7.69	0.02	-2.77	0.19	9.20	0.07	-0.77	0.08
71	7.79	0.11	-1.64	0.69	9.47	0.02	-1.09	0.04
72	7.22	0.06	-1.79	0.42	9.68	0.02	-1.12	0.04
73	8.04	0.06	-1.01	0.15	9.77	0.01	-1.04	0.03
74	6.46	0.06	-1.60	0.20	9.13	0.07	-0.73	0.07
75	6.44	0.26	-3.11	1.75	9.69	0.04	-0.80	0.05
76	6.69	0.02	-1.40	0.07	8.64	0.09	-2.20	0.54

77	6.33	0.18	-1.89	0.90	9.52	0.04	-0.74	0.04
78	7.18	0.05	-0.67	0.05	9.34	0.02	-0.89	0.02
79	7.19	0.04	-0.78	0.05	9.27	0.00	-1.40	0.01
80	6.12	0.13	-2.47	2.64	9.59	0.01	-1.27	0.03
81	6.46	0.15	-0.65	0.13	9.59	0.03	-1.28	0.09
82	6.85	0.03	-1.75	0.31	9.53	0.01	-1.26	0.01
83	7.08	0.10	-0.84	0.14	9.43	0.04	-0.78	0.04
84	7.34	0.04	-1.69	0.15	9.39	0.01	-1.20	0.01
85	7.73	0.04	-0.83	0.06	9.82	0.04	-0.86	0.07
86	6.01	0.03	-1.23	0.12	9.65	0.02	-1.29	0.05
87	7.03	0.03	-1.02	0.06	9.60	0.04	-0.76	0.05
88	5.73	0.09	-1.58	0.42	9.55	0.01	-1.30	0.04
89	7.40	0.04	-1.41	0.13	9.54	0.02	-0.79	0.03
90	6.85	0.12	-2.97	2.36	9.56	0.00	-0.92	0.01
91	6.23	0.02	-0.70	0.02	9.44	0.01	-1.09	0.01
92	7.26	0.31	-2.93	3.47	9.24	0.01	-1.29	0.04
93	7.71	0.06	-0.75	0.06	9.55	0.00	-1.18	0.01
94	5.99	0.05	-1.46	0.44	9.20	0.01	-0.85	0.02
95	6.05	0.05	-0.71	0.06	9.62	0.02	-0.91	0.02
96	6.48	0.01	-1.49	0.02	9.68	0.02	-1.19	0.04
97	6.80	0.01	-1.79	0.10	9.53	0.01	-1.29	0.02
98	6.56	0.04	-1.17	0.09	9.67	0.01	-1.07	0.03
99	6.80	0.03	-1.50	0.17	9.70	0.02	-0.91	0.04
100	6.80	0.43	-0.46	0.19	9.02	0.02	-1.04	0.05
average	6.93	0.56	-1.48	0.80	9.51	0.23	-1.08	0.29

Table S6. Individual pK_a values and Hill coefficients (n) calculated for Lys-207 and Lys-350 in Fab-**2** (*SR* diastereomer) through 100-independent 1 ns CpHMD simulations (σ = asymptotic standard error).

# replica	Lys-207				Lys-350			
	pK_a		n		pK_a		n	
	value	σ	value	σ	value	σ	value	σ
1	6.26	1.15	-4.26	19.03	9.66	0.02	-0.91	0.03
2	9.46	0.01	-2.35	0.05	9.24	0.07	-0.86	0.10
3	6.26	0.03	-0.96	0.06	9.33	0.02	-0.91	0.04
4	6.39	0.07	-0.94	0.11	9.64	0.05	-0.72	0.05
5	8.32	0.36	-0.37	0.10	9.22	0.01	-0.89	0.02
6	11.78	13.51	-6.13	374.98	8.92	0.11	-0.63	0.09
7	5.66	0.16	-2.01	0.86	9.38	0.06	-1.25	0.15
8	7.04	0.14	-0.74	0.16	9.44	0.05	-0.74	0.06
9	6.51	0.27	-3.33	1.79	8.81	0.03	-1.18	0.08
10	7.31	0.06	-1.31	0.19	9.26	0.08	-0.70	0.08
11	6.49	0.12	-0.64	0.10	9.52	0.02	-1.10	0.04
12	6.40	0.60	-0.39	0.18	9.61	0.09	-0.68	0.08
13	5.25	0.02	-1.89	0.15	9.49	0.00	-1.44	0.01
14	9.76	0.12	-1.92	0.87	8.62	0.07	-2.54	0.46
15	6.86	0.03	-2.34	0.54	9.67	0.00	-1.11	0.01
16	6.00	0.07	-0.89	0.11	9.53	0.01	-1.06	0.03
17	7.41	0.30	-0.40	0.10	8.90	0.01	-1.10	0.04
18	5.13	0.03	-1.29	0.15	9.62	0.01	-1.12	0.03
19	6.13	0.08	-0.83	0.12	9.37	0.03	-2.17	0.14
20	7.61	0.13	-3.89	1.26	9.42	0.02	-0.94	0.03
21	7.59	0.04	-2.52	0.22	8.75	0.09	-1.81	0.57
22	6.54	0.03	-0.92	0.05	9.42	0.02	-1.39	0.05

23	5.78	0.48	-3.15	6.88	8.96	0.05	-0.96	0.11
24	7.03	0.59	-0.35	0.15	8.86	0.02	-1.56	0.17
25	5.82	0.16	-2.27	2.01	9.01	0.06	-0.57	0.04
26	7.73	1.43	-3.31	17.37	9.65	0.00	-1.11	0.00
27	7.52	0.03	-1.52	0.09	8.95	0.05	-0.93	0.09
28	5.89	0.02	-1.31	0.10	9.53	0.01	-0.91	0.02
29	5.24	1.00	-3.62	14.88	9.75	0.02	-1.24	0.05
30	6.44	0.15	-2.94	0.98	9.56	0.12	-0.60	0.08
31	6.15	0.05	-2.03	0.68	8.76	0.19	-0.62	0.15
32	6.02	0.23	-1.05	0.61	8.99	0.01	-1.31	0.06
33	7.13	0.05	-3.00	1.09	9.48	0.03	-1.45	0.08
34	9.52	0.34	-0.40	0.11	9.56	0.03	-1.62	0.09
35	7.52	0.40	-0.46	0.17	9.38	0.11	-1.85	0.49
36	7.24	0.16	-0.47	0.07	9.62	0.01	-1.13	0.03
37	6.98	0.04	-2.45	4.23	9.14	0.03	-0.90	0.06
38	7.57	0.16	-2.80	0.95	9.03	0.05	-0.91	0.10
39	5.63	0.04	-2.91	0.33	9.59	0.02	-1.80	0.09
40	7.60	0.19	-3.54	1.64	9.11	0.02	-1.21	0.07
41	5.26	0.03	-2.47	0.25	8.96	0.05	-1.00	0.11
42	7.76	0.05	-1.93	0.39	9.55	0.01	-0.83	0.01
43	5.98	0.05	-0.80	0.07	9.47	0.02	-1.38	0.05
44	6.59	0.16	-0.53	0.09	9.68	0.02	-0.99	0.03
45	7.22	0.16	-1.83	1.12	8.66	0.34	-0.44	0.13
46	6.15	0.08	-1.99	0.97	9.79	0.02	-1.00	0.04
47	6.65	0.31	-0.47	0.14	9.42	0.02	-0.94	0.03
48	6.91	0.06	-0.92	0.11	9.63	0.01	-0.97	0.02
49	7.28	0.09	-0.91	0.15	9.58	0.02	-0.87	0.03
50	6.42	0.07	-1.65	0.25	9.51	0.05	-0.75	0.05
51	6.69	0.02	-2.22	0.15	9.25	0.02	-1.10	0.04
52	6.16	11.61	-5.01	361.03	9.72	0.00	-1.16	0.01
53	6.23	0.03	-1.27	0.10	8.94	0.17	-0.59	0.12
54	6.27	0.09	-1.75	0.47	9.56	0.02	-0.97	0.03
55	7.50	0.15	-2.40	0.73	9.30	0.01	-1.30	0.04
56	8.35	0.04	-1.28	0.11	8.75	0.04	-0.96	0.07
57	5.64	0.20	-0.52	0.11	9.53	0.03	-1.17	0.08
58	10.81	29.38	-5.01	757.69	8.43	0.10	-0.64	0.08
59	6.69	0.18	-2.42	1.34	9.46	0.10	-2.12	0.42
60	6.97	0.11	-0.57	0.07	9.72	0.00	-1.02	0.01
61	6.75	0.02	-0.76	0.02	9.41	0.00	-1.06	0.01
62	6.70	0.59	-0.31	0.11	9.09	0.01	-1.45	0.04
63	6.27	0.18	-3.41	2.23	9.75	0.03	-0.88	0.05
64	5.18	0.08	-1.76	0.61	9.43	0.00	-1.01	0.01
65	10.79	0.02	-3.28	0.35	9.45	0.02	-0.99	0.03
66	6.64	0.04	-1.84	0.17	9.42	0.01	-1.12	0.03
67	6.76	0.01	-1.33	0.04	9.76	0.02	-1.01	0.03
68	10.76	0.90	-2.96	11.04	9.41	0.07	-1.52	0.22
69	7.04	0.21	-0.56	0.13	9.25	0.02	-1.09	0.04
70	6.64	0.01	-1.34	0.03	9.37	0.01	-1.37	0.02
71	6.14	0.04	-2.11	0.49	9.56	0.10	-0.61	0.07
72	7.34	0.09	-0.60	0.07	9.15	0.01	-1.10	0.02
73	7.51	0.02	-1.43	0.06	9.15	0.02	-0.92	0.04
74	5.26	0.11	-4.29	1.89	9.19	0.01	-1.28	0.05
75	7.01	0.10	-0.75	0.11	9.40	0.01	-0.96	0.02
76	8.33	0.16	-4.31	2.10	9.66	0.04	-1.17	0.09
77	6.33	0.02	-1.98	0.09	9.59	0.01	-1.24	0.02
78	10.47	0.09	-0.78	0.11	8.85	0.01	-0.87	0.02
79	6.86	0.21	-0.39	0.07	8.63	0.06	-2.40	0.35
80	8.58	0.28	-0.45	0.11	9.22	0.04	-0.82	0.05

81	6.83	0.03	-0.76	0.04	9.72	0.04	-0.81	0.05
82	5.70	0.11	-1.55	0.43	9.43	0.01	-1.61	0.03
83	5.47	0.05	-1.58	0.16	9.24	0.05	-0.80	0.07
84	5.55	0.65	-4.45	6.28	9.57	0.02	-1.34	0.05
85	7.82	0.06	-2.16	0.70	9.15	0.06	-0.92	0.11
86	7.38	0.56	-3.50	5.03	9.04	0.02	-0.89	0.04
87	6.96	0.47	-0.36	0.12	9.56	0.01	-1.11	0.02
88	7.33	0.14	-2.20	0.89	9.37	0.05	-0.74	0.05
89	9.19	0.04	-2.90	0.56	9.07	0.15	-0.65	0.13
90	5.60	0.05	-0.80	0.07	9.49	0.00	-1.09	0.00
91	6.13	0.46	-2.42	8.63	8.81	0.03	-1.93	0.22
92	9.01	0.01	-1.56	0.16	9.60	0.00	-1.13	0.01
93	6.32	0.23	-0.53	0.13	9.64	0.05	-0.73	0.06
94	7.62	3.76	-4.89	48.53	9.74	0.06	-0.75	0.07
95	5.50	0.10	-0.68	0.09	9.37	0.00	-1.19	0.01
96	5.12	0.04	-2.24	0.73	9.05	0.01	-1.64	0.13
97	6.50	0.13	-0.58	0.09	8.76	0.02	-1.94	0.12
98	6.43	0.07	-2.73	0.42	9.51	0.01	-0.94	0.02
99	11.42	0.10	-0.86	0.14	8.29	0.00	-1.25	0.01
100	6.64	0.01	-0.96	0.02	9.78	0.02	-0.95	0.04
average	7.02	1.40	-1.86	1.29	9.32	0.33	-1.11	0.40

Table S7. Individual pK_a values and Hill coefficients (n) calculated for Lys-207 and Lys-350 in Fab-**2** (*SS* diastereomer) through 100-independent 1 ns CpHMD simulations (σ = asymptotic standard error).

**# replica**	**Lys-207**				**Lys-350**			
	**pK_a**		**n**		**pK_a**		**n**	
	**value**	**σ**	**value**	**σ**	**value**	**σ**	**value**	**σ**
1	5.12	0.27	-1.02	0.62	9.45	0.01	-1.10	0.02
2	7.09	0.07	-1.09	0.20	9.27	0.02	-1.53	0.07
3	5.74	0.01	-2.94	0.15	9.29	0.02	-1.49	0.10
4	6.83	0.07	-2.27	0.92	9.53	0.01	-1.28	0.03
5	6.12	0.21	-4.33	7.49	9.63	0.01	-0.84	0.01
6	7.14	0.09	-0.96	0.17	9.68	0.00	-1.04	0.01
7	6.63	0.05	-1.66	0.21	9.71	0.01	-0.96	0.02
8	7.57	0.01	-1.22	0.02	9.59	0.01	-0.99	0.01
9	6.83	0.02	-1.33	0.09	9.43	0.01	-1.03	0.01
10	8.75	0.02	-1.77	0.11	9.18	0.02	-1.10	0.05
11	6.75	0.06	-0.83	0.08	9.48	0.02	-1.69	0.06
12	10.48	0.10	-2.04	0.43	8.22	0.08	-0.70	0.08
13	7.26	0.11	-1.41	0.43	9.57	0.08	-0.86	0.11
14	6.35	0.02	-2.04	0.12	8.65	0.13	-0.55	0.08
15	7.85	0.06	-2.41	0.93	8.74	0.10	-0.96	0.19
16	6.84	0.04	-0.83	0.06	9.45	0.07	-0.68	0.06
17	6.43	1.03	-4.43	10.39	9.45	0.01	-1.13	0.03
18	8.19	0.07	-1.44	0.36	9.46	0.03	-0.80	0.03
19	6.68	0.05	-2.25	0.32	9.86	0.01	-1.40	0.07
20	7.01	0.02	-0.94	0.04	9.25	0.06	-1.22	0.18
21	8.38	0.04	-2.17	0.20	9.53	0.03	-0.84	0.04
22	7.20	0.96	-5.10	24.89	9.43	0.02	-1.18	0.05
23	8.82	0.06	-1.77	0.45	9.00	0.07	-0.86	0.12
24	6.06	0.06	-0.79	0.08	9.79	0.02	-0.99	0.03
25	6.31	0.07	-1.46	0.25	8.82	0.01	-1.18	0.03
26	6.17	0.16	-0.64	0.14	8.67	0.01	-1.02	0.03

27	6.69	0.09	-1.21	0.24	9.43	0.05	-0.76	0.06
28	6.44	0.02	-1.23	0.05	9.34	0.01	-1.01	0.02
29	7.04	0.09	-1.02	0.21	9.76	0.01	-1.00	0.03
30	7.73	0.02	-1.90	0.11	9.19	0.17	-3.33	3.01
31	6.98	0.07	-1.22	0.27	9.76	0.11	-0.63	0.09
32	10.29	0.61	-0.33	0.13	9.19	0.06	-1.14	0.17
33	7.37	0.00	-1.73	0.02	8.42	0.09	-2.02	0.41
34	5.57	0.19	-0.49	0.09	9.33	0.01	-1.06	0.01
35	6.15	0.14	-2.30	1.97	8.40	0.07	-0.66	0.06
36	6.70	0.03	-0.96	0.06	9.30	0.05	-0.76	0.06
37	6.19	0.55	-3.84	11.42	9.48	0.02	-1.56	0.05
38	6.23	0.28	-2.53	3.00	9.43	0.01	-1.04	0.02
39	6.94	0.03	-0.79	0.04	8.97	0.07	-0.93	0.14
40	7.88	0.10	-0.77	0.12	9.46	0.02	-1.59	0.07
41	6.96	0.01	-1.80	0.17	9.89	0.06	-0.78	0.08
42	6.97	0.45	-0.35	0.11	9.71	0.02	-1.07	0.05
43	7.44	0.40	-5.25	4.71	9.44	0.02	-0.90	0.03
44	7.41	0.54	-3.35	4.23	9.35	0.03	-1.34	0.08
45	6.92	0.01	-2.37	0.19	9.37	0.03	-2.11	0.15
46	6.67	0.15	-1.31	0.44	9.71	0.01	-1.12	0.03
47	7.71	0.28	-0.48	0.13	9.25	0.00	-1.10	0.01
48	5.99	0.06	-1.32	0.34	9.51	0.03	-1.45	0.09
49	6.53	0.10	-1.22	0.23	9.77	0.05	-0.79	0.06
50	6.07	0.04	-1.45	0.30	9.52	0.03	-0.78	0.04
51	6.48	0.03	-3.41	0.19	9.71	0.02	-0.81	0.03
52	7.29	0.02	-1.42	0.06	9.37	0.02	-1.00	0.03
53	8.49	0.01	-1.36	0.01	9.94	0.07	-0.82	0.10
54	6.54	0.08	-0.86	0.11	9.46	0.01	-1.15	0.02
55	7.46	0.01	-1.45	0.03	9.30	0.02	-1.01	0.04
56	9.55	0.01	-3.45	0.11	8.95	0.02	-1.18	0.07
57	7.55	0.27	-0.45	0.11	9.30	0.01	-0.92	0.02
58	7.56	0.06	-3.50	0.49	9.24	0.00	-1.07	0.01
59	7.40	0.12	-0.59	0.09	9.74	0.02	-1.00	0.04
60	6.54	0.04	-2.31	0.18	9.47	0.01	-1.08	0.02
61	6.70	0.03	-1.25	0.09	9.53	0.01	-1.13	0.02
62	7.51	0.03	-1.03	0.05	9.67	0.02	-0.79	0.03
63	6.64	0.09	-1.08	0.19	9.83	0.03	-1.04	0.08
64	7.34	0.01	-0.97	0.02	9.61	0.03	-0.81	0.04
65	6.89	0.14	-0.47	0.06	9.60	0.01	-0.98	0.02
66	7.02	0.06	-0.60	0.04	9.48	0.02	-0.96	0.03
67	6.15	0.02	-2.72	0.44	9.47	0.01	-1.09	0.02
68	6.62	0.03	-0.89	0.05	9.37	0.04	-1.51	0.14
69	7.26	0.02	-2.31	0.20	8.97	0.04	-0.84	0.06
70	6.79	0.01	-3.71	0.25	9.36	0.02	-0.92	0.04
71	6.84	0.14	-0.62	0.11	9.48	0.09	-0.66	0.08
72	6.94	0.09	-0.78	0.11	9.71	0.01	-0.84	0.02
73	6.80	0.03	-2.14	0.33	9.61	0.06	-0.80	0.07
74	6.87	0.04	-0.58	0.03	9.14	0.05	-0.83	0.08
75	6.55	0.01	-2.12	0.06	9.41	0.01	-1.07	0.02
76	6.56	0.02	-1.60	0.08	9.47	0.01	-1.58	0.02
77	7.37	0.13	-2.44	0.82	9.45	0.01	-1.35	0.02
78	6.52	0.08	-1.23	0.20	9.44	0.02	-1.29	0.05
79	6.14	0.12	-0.63	0.10	9.61	0.02	-1.21	0.04
80	7.52	0.03	-1.68	0.10	8.84	0.00	-1.84	0.03
81	5.28	0.27	-0.43	0.10	9.22	0.02	-1.57	0.09
82	8.07	0.29	-0.57	0.19	9.55	0.02	-1.75	0.06
83	7.44	0.16	-0.54	0.09	9.76	0.02	-0.96	0.05
84	7.38	0.18	-2.56	1.11	9.26	0.01	-1.39	0.03

85	7.17	0.02	-2.29	0.31	9.47	0.01	-0.89	0.02
86	6.88	0.06	-1.69	0.54	9.57	0.01	-0.92	0.02
87	8.56	0.10	-0.77	0.12	9.43	0.01	-1.20	0.02
88	10.33	0.40	-0.31	0.08	9.46	0.01	-1.61	0.04
89	8.24	0.22	-0.45	0.09	9.54	0.02	-1.16	0.05
90	6.71	0.02	-2.11	0.17	9.51	0.01	-1.10	0.03
91	6.30	0.15	-2.86	1.38	9.08	0.07	-0.88	0.11
92	6.34	0.04	-3.09	0.37	9.46	0.08	-0.66	0.07
93	5.12	1.02	-3.11	26.35	9.32	0.00	-1.18	0.01
94	8.01	0.07	-0.95	0.15	9.39	0.09	-0.58	0.06
95	5.43	0.29	-0.45	0.12	9.07	0.03	-1.00	0.08
96	5.29	0.14	-2.95	1.44	8.76	0.05	-1.99	0.35
97	6.89	0.07	-0.77	0.09	9.75	0.01	-1.21	0.02
98	5.96	0.25	-0.49	0.13	9.64	0.01	-0.93	0.02
99	7.13	0.01	-1.29	0.06	9.55	0.01	-1.12	0.02
100	7.75	1.30	-4.77	25.09	9.24	0.02	-1.63	0.11
average	7.04	0.99	-1.68	1.13	9.39	0.32	-1.12	0.39

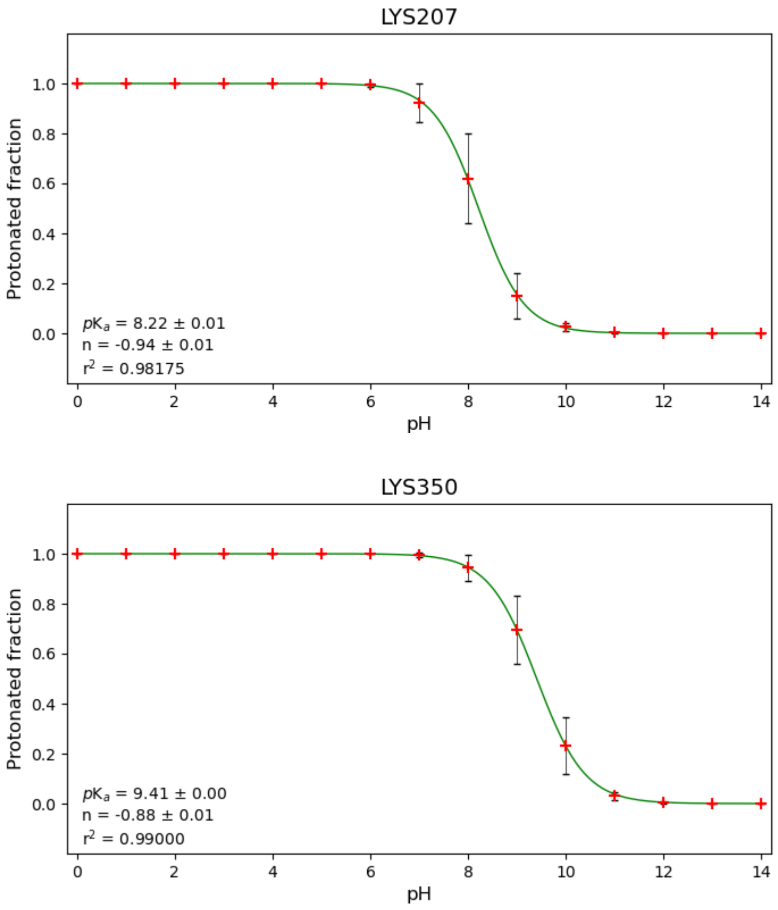

Figure S24. pK_a values and Hill coefficients (n) calculated for Lys-207 and Lys-350 in unligated Fab-**2** by averaging their protonated fractions (1 – f_d) as a function of the pH across 100-independent 1 ns CpHMD simulations. Error bars indicate the dispersion (standard deviation) of (1 – f_d) at a given pH and are maximum near the calculated pK_a. The asymptotic standard errors for the calculated pK_a and n values (± σ) derived from the non-linear fitting, as well as the correlation coefficient (r^2), are provided.

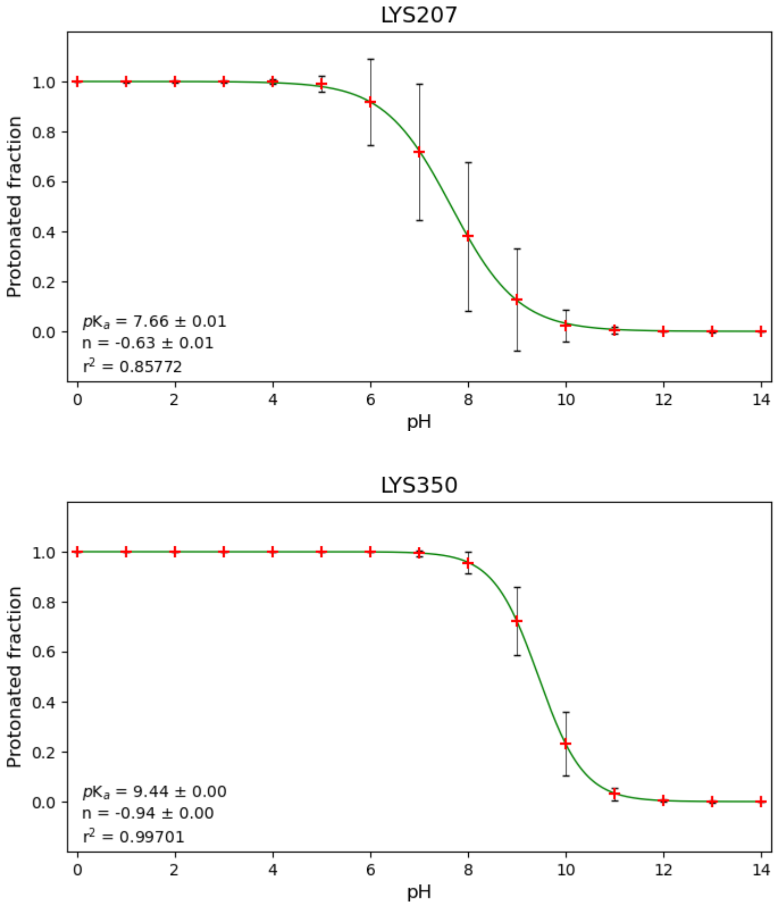

Figure S25. pK_a values and Hill coefficients (n) calculated for Lys-207 and Lys-350 in Fab-**2** (*RR* diastereomer) by averaging their protonated fractions ($1 - f_d$) as a function of the pH across 100-independent 1 ns CpHMD simulations. Error bars indicate the dispersion (standard deviation) of ($1 - f_d$) at a given pH and are maximum near the calculated pK_a. The asymptotic standard errors for the calculated pK_a and n values (± σ) derived from the non-linear fitting, as well as the correlation coefficient (r^2), are provided.

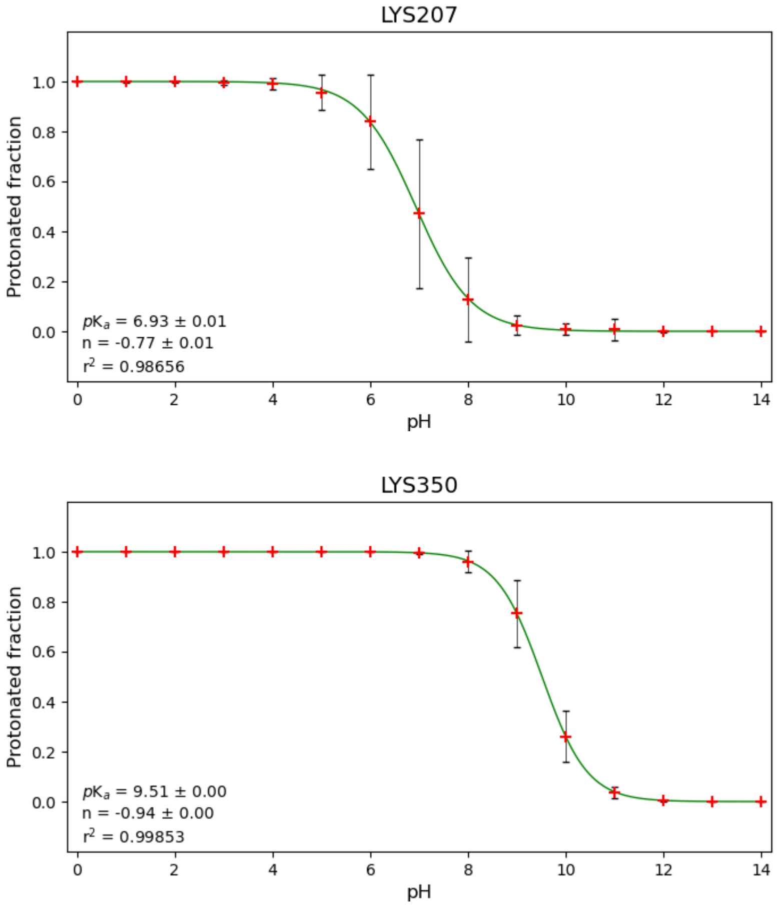

Figure S26. pK_a values and Hill coefficients (n) calculated for Lys-207 and Lys-350 in Fab-**2** (*RS* diastereomer) by averaging their protonated fractions (1 – f_d) as a function of the pH across 100-independent 1 ns CpHMD simulations. Error bars indicate the dispersion (standard deviation) of (1 – f_d) at a given pH and are maximum near the calculated pK_a. The asymptotic standard errors for the calculated pK_a and n values (± σ) derived from the non-linear fitting, as well as the correlation coefficient (r^2), are provided.

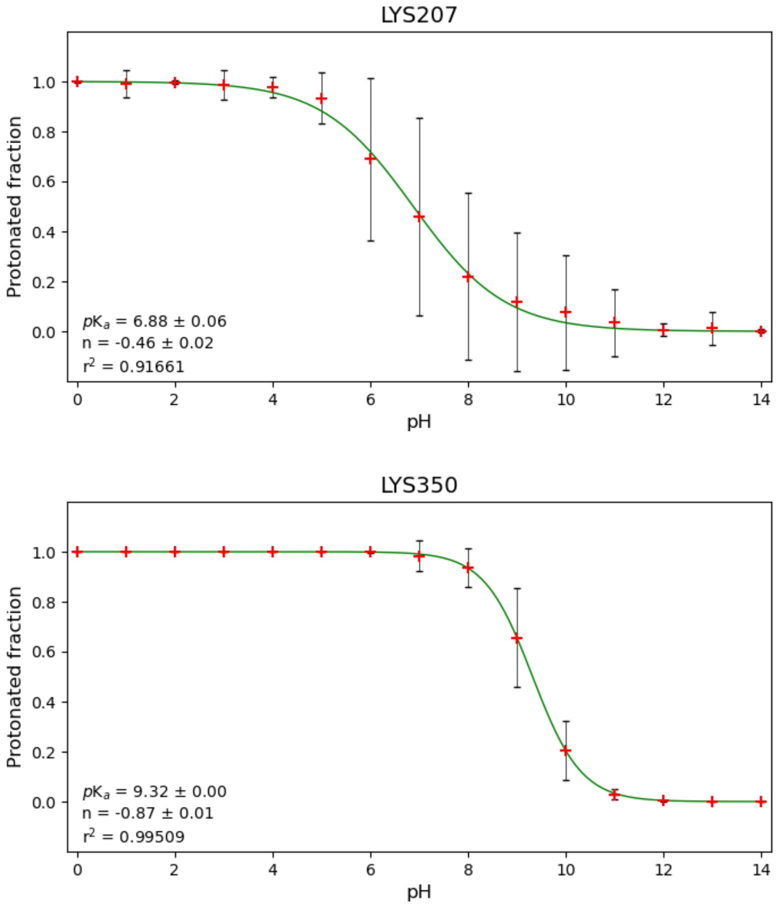

Figure S27. pK_a values and Hill coefficients (n) calculated for Lys-207 and Lys-350 in Fab-**2** (*SR* diastereomer) by averaging their protonated fractions (1 – f_d) as a function of the pH across 100-independent 1 ns CpHMD simulations. Error bars indicate the dispersion (standard deviation) of (1 – f_d) at a given pH and are maximum near the calculated pK_a. The asymptotic standard errors for the calculated pK_a and n values (± σ) derived from the non-linear fitting, as well as the correlation coefficient (r^2), are provided.

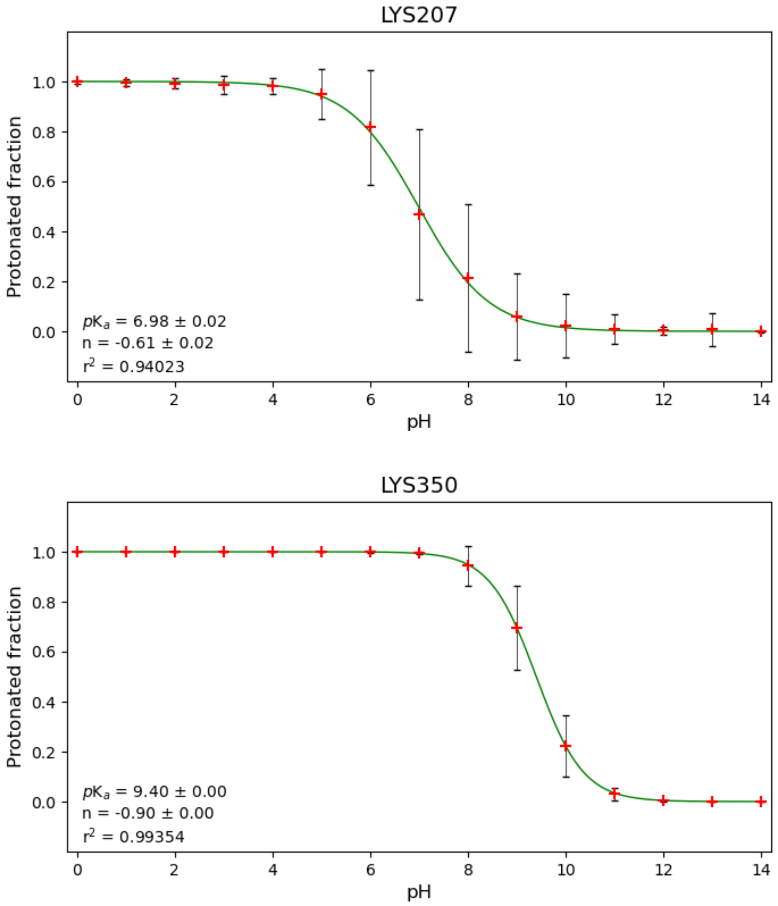

Figure S28. pK_a values and Hill coefficients (n) calculated for Lys-207 and Lys-350 in Fab-**2** (*SS* diastereomer) by averaging their protonated fractions ($1 - f_d$) as a function of the pH across 100-independent 1 ns CpHMD simulations. Error bars indicate the dispersion (standard deviation) of ($1 - f_d$) at a given pH and are maximum near the calculated pK_a. The asymptotic standard errors for the calculated pK_a and n values (± σ) derived from the non-linear fitting, as well as the correlation coefficient (r^2), are provided.

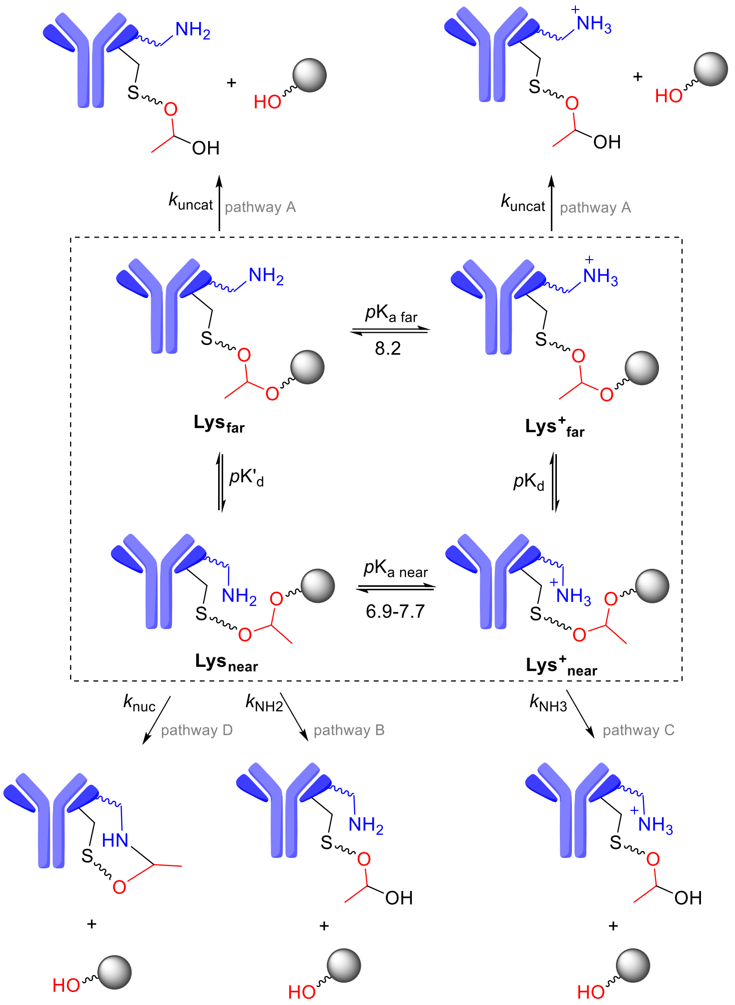

Scheme S7. All possible reaction pathways for pro-duocarmycin (grey circle) release studied computationally from ligated Fab-**2**. Dashed rectangle highlights the reactive species that are in equilibrium in solution, including neutral or protonated Lys-207 (Lys and Lys^+) close to (Lys_{near}) or away from (Lys_{far}) the acetal group. If Lys-207 is far from the acetal group, the only available pathway is the uncatalyzed hydrolysis of the acetal group (pathway A). On the other hand, if Lys-207 is near the acetal group and protonated (Lys^+_{near}), it can follow pathway B, and if it is neutral (Lys_{near}), it can follow either pathway C or D.

Derivation of k_{theo} as a function of *pH*

There are four proposed pathways (A-D) leading to lysine-assisted or non-assisted pro-duocarmycin release (Scheme S7). Hence, the global reaction rate can be written as:

$$k_{theo}[Fab2] = k_{uncat}[Lys_{far}] + k_{uncat}[Lys^+_{far}] + k_{NH3}[Lys^+_{near}] + k_{NH2}[Lys_{near}] + k_{nuc}[Lys_{near}] \quad \text{(S2)}$$

The lysine acidity (K_a) and lysine-acetal pair formation (K_d) equilibrium constants, and the mass-balance equation are:

$$K_{a\,far} = \frac{[Lys_{far}][H^+]}{[Lys^+_{far}]} \qquad K_{a\,near} = \frac{[Lys_{near}][H^+]}{[Lys^+_{near}]} \qquad K_d = \frac{[Lys^+_{near}]}{[Lys^+_{far}]} \qquad K'_d = \frac{[Lys_{near}]}{[Lys_{far}]}$$

$$[Fab2] = [Lys_{far}] + [Lys^+_{far}] + [Lys^+_{near}] + [Lys_{near}]$$

Therefore, substituting [Fab2] in equation S2:

$$k_{theo} = \frac{k_{uncat}[Lys_{far}] + k_{uncat}[Lys^+_{far}] + k_{NH3}[Lys^+_{near}] + k_{NH2}[Lys_{near}] + k_{nuc}[Lys_{near}]}{[Lys_{far}] + [Lys^+_{far}] + [Lys^+_{near}] + [Lys_{near}]} \quad \text{(S3)}$$

Using equilibrium constants to write all the concentrations as a function of [Lys$_{far}$] and substituting in Equation S3:

$$k_{theo} = \frac{k_{uncat}[Lys_{far}] + k_{uncat}\frac{[H^+]}{K_{a\,far}}[Lys_{far}] + k_{NH3}\frac{K_d[H^+]}{K_{a\,far}}[Lys_{far}] + k_{NH2}\frac{K_dK_{a\,near}}{K_{a\,far}}[Lys_{far}] + k_{nuc}\frac{K_dK_{a\,near}}{K_{a\,far}}[Lys_{far}]}{[Lys_{far}] + \frac{[H^+]}{K_{a\,far}}[Lys_{far}] + \frac{K_d[H^+]}{K_{a\,far}}[Lys_{far}] + \frac{K_dK_{a\,near}}{K_{a\,far}}[Lys_{far}]} \quad \text{(S4)}$$

[Lys$_{far}$] is a common term in both the numerator and denominator of Equation S4. Hence:

$$k_{theo} = \frac{k_{uncat} + k_{uncat}\frac{[H^+]}{K_{a\,far}} + k_{NH3}\frac{K_d[H^+]}{K_{a\,far}} + k_{NH2}\frac{K_dK_{a\,near}}{K_{a\,far}} + k_{nuc}\frac{K_dK_{a\,near}}{K_{a\,far}}}{1 + \frac{[H^+]}{K_{a\,far}} + \frac{K_d[H^+]}{K_{a\,far}} + \frac{K_dK_{a\,near}}{K_{a\,far}}} \quad \text{(S5)}$$

Multiplying the right side of Equation S5 by $\frac{K_{a\,far}}{K_{a\,far}}$:

$$k_{theo} = \frac{k_{uncat}K_{a\,far} + k_{uncat}[H^+] + k_{NH3}\,K_d[H^+] + k_{NH2}K_dK_{a\,near} + k_{nuc}K_dK_{a\,near}}{K_{a\,far} + [H^+] + K_d[H^+] + K_dK_{a\,near}} \quad \text{(S6)}$$

Rearranging Equation S6:

$$k_{theo} = \frac{(k_{uncat} + k_{NH3}\,K_d)[H^+] + (k_{NH2} + k_{nuc})K_dK_{a\,near} + k_{uncat}K_{a\,far}}{(1 + K_d)[H^+] + K_{a\,far} + K_dK_{a\,near}} \quad \text{(S7)}$$

$$\boldsymbol{k_{theo} = \frac{(k_{uncat} + k_{NH3}\,K_d) \cdot 10^{-pH} + (k_{NH2} + k_{nuc})K_dK_{a\,near} + k_{uncat}K_{a\,far}}{(1 + K_d) \cdot 10^{-pH} + K_{a\,far} + K_dK_{a\,near}}} \quad \text{(S8)}$$

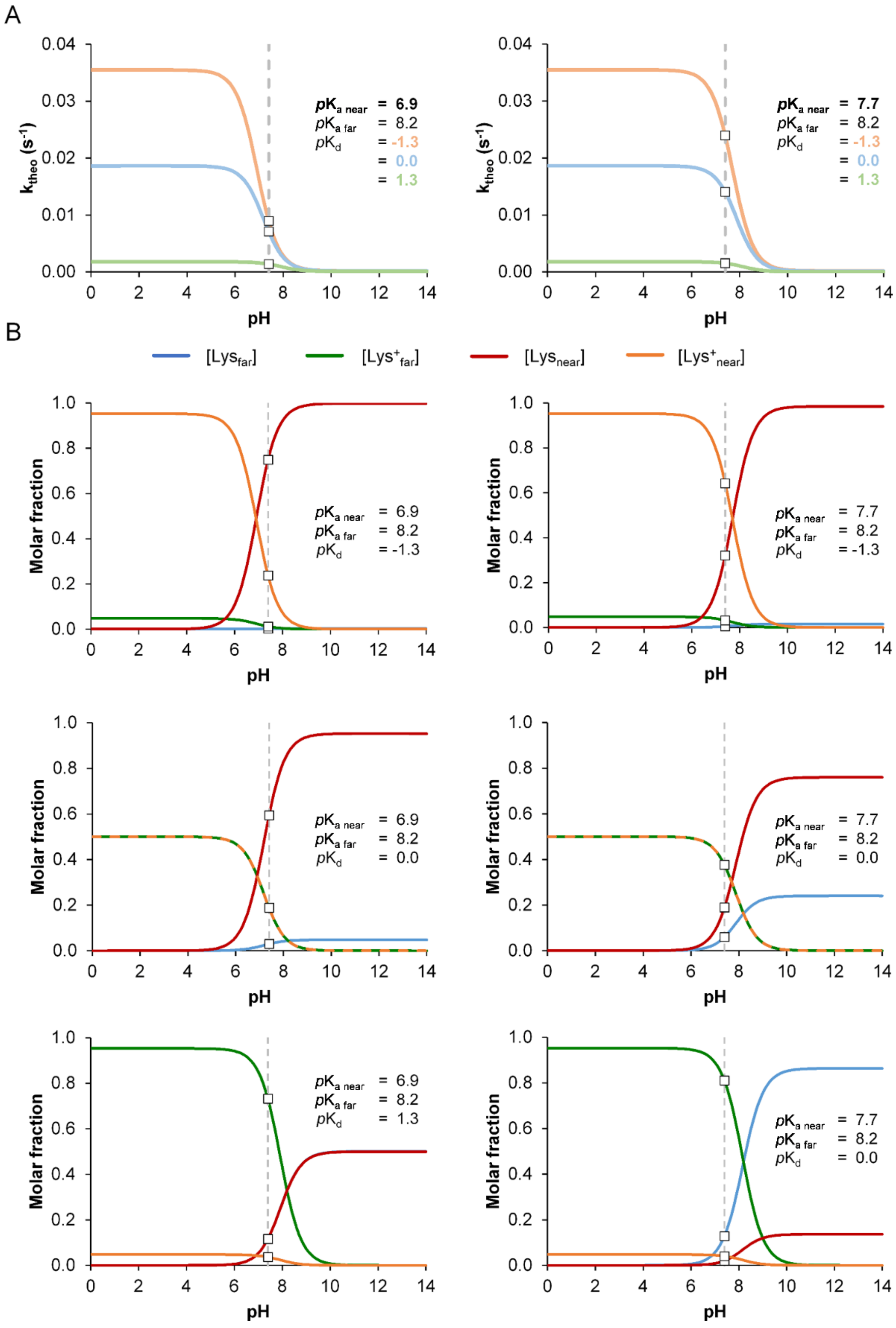

k_{uncat} = 2.6 · 10^{-8} s^{-1} (from calculated ΔG‡ = 27.8 kcal mol^{-1}; T = 298.15 K)

k_{NH3} = 3.7 · 10^{-2} s^{-1} (from calculated ΔG‡ = 19.4 kcal mol^{-1}; T = 298.15 K)

k_{NH2} = 8.3 · 10^{-11} s^{-1} (from calculated ΔG‡ = 31.2 kcal mol^{-1}; T = 298.15 K)

k_{nuc} = 1.7 · 10^{-4} s-^{1} (from calculated ΔG‡ = 22.6 kcal mol^{-1}; T = 298.15 K)

Figure S29. A) Variation of theoretical reaction rates (k_{theo}) for pro-duocarmycin release as a function of pH (equation 7). B) Variation of the theoretical molar fraction of the four reactive species (Lys_{far}, Lys^{+}_{far}, Lys_{near}, and Lys^{+}_{near}) as a function of pH. For illustrative purposes, different lysine acidities derived from CpHMD simulations (see below) for Lys-207 in conformations close to the acetal group ($pK_{a\ near}$) and distal Lys-350 ($pK_{a\ far}$) were considered. Arbitrary values to account for scenarios in which Lys^{+} is mostly away from (K_d = 0.05; pK_d = 1.3), close to (K_d = 20; pK_d = -1.3) or equally away from/close to (K_d = 1; pK_d = 0) the acetal group were considered. White squares represent k_{theo} values at physiological pH (7.4, dashed grey line). As expected, k_{theo} is maximum at low pH values, where protonated Lys^{+} is the main species and pathway C dominates. On the other hand, at high pH values where neutral Lys is the main species and pathway D becomes dominant, lower k_{theo} values are obtained. At physiological pH 7.4, k_{theo} as well as the concentrations of the reactive species are extremely sensitive to slight variations on the values of the different equilibrium constants, highlighting the importance of considering all the plausible mechanisms involved in acetal cleavage to obtain a realistic picture of the observed reaction.

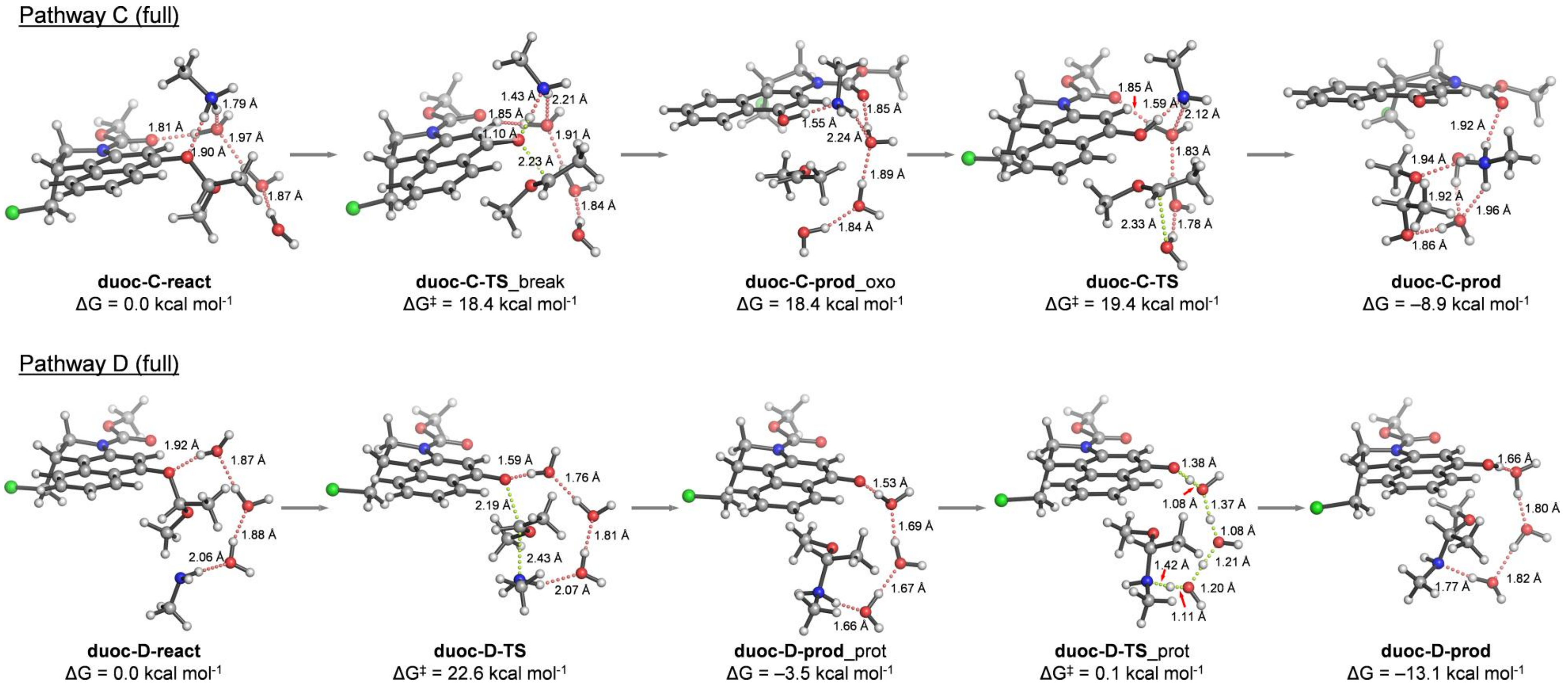

Figure S30. Geometries and relatives stabilities for the reactants, transition states, intermediates and products for the whole computed pathways C (ammonium-assisted hydrolysis) and D (water-assisted aminolysis) for acetal cleavage calculated with PCM(H_2O)/M06-2X/6-31+G(d,p). Relative free energies (ΔG and $\Delta G^{\ddagger}$) are given in kcal mol^{-1} and interatomic distances in angstroms. Hydrogen-bond interactions are shown as dotted red lines. Breaking/forming bonds on the transition states are shown as dotted green lines.

Table S8. Energies, entropies, and lowest frequencies of the lowest energy calculated structures.[a]

Structure	E_{elec} (Hartree)	E_{elec} + ZPE (Hartree)	H (Hartree)	S (cal mol^{-1} K^{-1})	G (Hartree)	Lowest freq. (cm^{-1})	# imag freq.
**duoc-*OH*-react**	–1320.365451	–1320.096238	–1320.077823	136.0	–1320.140081	35.8	0
**duoc-*OH*-TS**_cycpr	–1320.321592	–1320.054487	–1320.036129	135.8	–1320.098244	–548.7	1
**duoc-*OH*-prod**_cycpr	–859.989109	–859.720984	–859.704648	124.0	–859.762256	41.6	0
**duoc-*OH*-TS**_H-shift	–1320.296544	–1320.032783	–1320.014034	139.4	–1320.076784	–919.1	1
**duoc-*OH*-prod**_H-shift	–860.011857	–859.745649	–859.728371	129.1	–859.787774	33.7	0
**duoc-*OH*-TS**_ring-exp	–1320.283933	–1320.018696	–1319.999592	141.8	–1320.063210	–323.8	1
**duoc-*OH*-prod**_ring-exp	–859.994390	–859.726106	–859.709484	127.1	–859.767544	20.6	0
**duoc-*O*-react**	–1319.892466	–1319.636899	–1319.618768	135.3	–1319.680645	35.6	0
**duoc-*O*-TS**_cycpr	–1319.867119	–1319.613280	–1319.595224	134.8	–1319.656884	–565.6	1
**duoc-*O*-prod**_cycpr	–859.559708	–859.304495	–859.288371	123.5	–859.345701	39.1	0
**duoc-*O*-TS**_H-shift	–1319.836184	–1319.585614	–1319.567222	137.7	–1319.629480	–984.1	1
**duoc-*O*-prod**_H-shift	–859.578708	–859.325140	–859.308057	128.8	–859.367232	29.1	0
**duoc-*O*-TS**_ring-exp	–1319.819801	–1319.567656	–1319.548979	139.9	–1319.611878	–324.2	1
**duoc-*O*-prod**_ring-exp	–859.565521	–859.310068	–859.293651	126.3	–859.351470	25.6	0
**Cl-**	–460.346502	–460.346502	–460.344142	36.6	–460.361525		0
**duoc-A-react**	–1819.083863	–1818.626701	–1818.590300	225.2	–1818.687418	16.8	0
**duoc-A-TS**	–1819.038563	–1818.583685	–1818.548660	216.4	–1818.643044	–241.4	1
**duoc-A-prod**	–1819.096993	–1818.638201	–1818.603082	215.7	–1818.697582	17.9	0
**duoc-B-react**	–1838.497475	–1837.998780	–1837.960981	233.7	–1838.061571	13.3	0
**duoc-B-TS**	–1838.448623	–1837.951362	–1837.914858	226.0	–1838.012377	–258.4	1
**duoc-B-prod**	–1838.510273	–1838.010552	–1837.973688	227.0	–1838.071831	18.8	0
**duoc-C-react**	–1838.955855	–1838.440230	–1838.403750	222.3	–1838.501023	16.7	0
**duoc-C-TS**_break	–1838.919091	–1838.409759	–1838.372485	225.5	–1838.471653	–164.1	1
**duoc-C-prod**_oxo	–1838.919666	–1838.409137	–1838.371087	228.4	–1838.471672	21.0	0
**duoc-C-TS**	–1838.919017	–1838.408466	–1838.371277	225.7	–1838.470059	–34.8	1
**duoc-C-prod**	–1838.972487	–1838.454822	–1838.418658	216.1	–1838.515260	27.1	0
**duoc-D-react**	–1838.491413	–1837.993065	–1837.954509	237.8	–1838.056510	13.8	0
**duoc-D-TS**	–1838.454268	–1837.958073	–1837.920360	233.4	–1838.020472	–358.4	1
**duoc-D-prod**_prot	–1838.502762	–1838.001848	–1837.965912	221.3	–1838.062027	18.0	0
**duoc-D-TS**_prot	–1838.491380	–1837.998359	–1837.964715	209.8	–1838.056375	–836.7	1
**duoc-D-prod**	–1838.517986	–1838.017191	–1837.981133	219.6	–1838.077393	23.2	0

[a]Energy values calculated at the PCM(H_2O)/M06-2X/6-31+G(d,p) level. 1 Hartree = 627.51 kcal mol^{-1}. Thermal corrections at 298.15 K.

Determination of the hydrolysis rate constants

Determination of the hydrolysis rate constant (k_1) of acetal 5

The hydrolysis rate constants (*k1*) of acetal **5** was determined at pH = 5.7 (NaP_i buffer 0.1 M, pH = 5.7) and 37 ºC by monitoring the reaction through Ultra Performance Liquid Chromatography-Mass Spectrometer (UPLC-MS) (Bruker micrOTOF-Q). Column: ACQUITY UPLC BEH C18 1,7; diameter: 2.1 mm and length: 100 mm). The samples (60 μL of the acetal solution diluted with 600 μL of MeOH) were eluted using the following gradient: 1:99 to 60:40 $CH_3CN:H_2O$ for 6 min, then 60:40 to 100:0 $CH_3CN:H_2O$ over 0.5 minutes, with a flow of 0.45 mL/min

Initial concentration (t = 0 s) of acetal **5** was 600 μM (see Table S9 and Figure S31).

Retention time (acetal **5**) = 5.05 min

Retention time (duocarmycin derivative **I**) = 4.48 min

Table S9. Peak area (A) and average peak area ($Å^2$) at different times.

A	t_0	t_{12h}	t_{24h}	t_{36h}	t_{48h}
A_1	2817997	2610334	1873248	1240861	971058
A_2	2878015	2659622	1873048	1280706	968943
A_3	2963179	2667561	1891647	1272442	886288
$Å^2$	2886397	2645839	1879314	1264670	942096

k_1 (**5**) = $5.0 \times 10^{-6} \pm 0.8 \times 10^{-6}$ s^{-1}

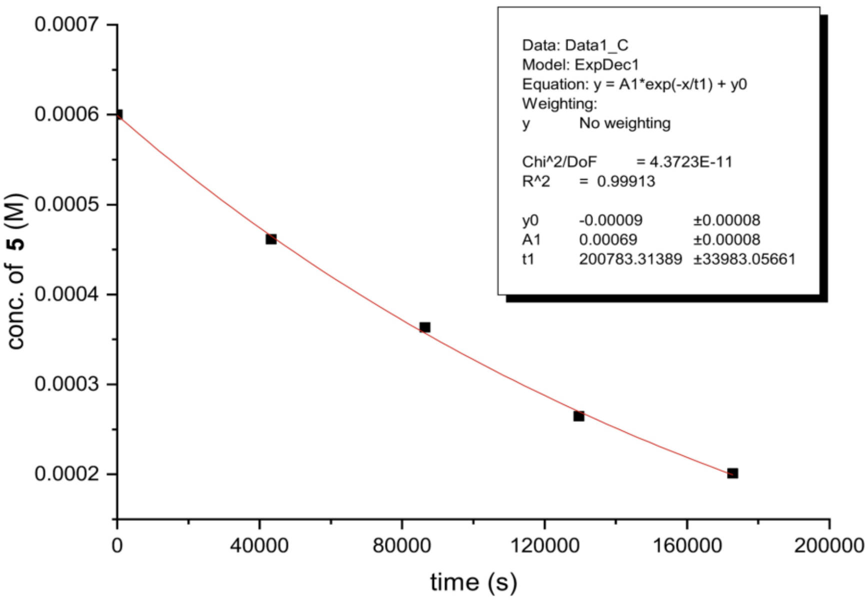

Figure S31. Hydrolysis rate constants (k_1) determined by UPLC-MS for acetal **5** at 37 ºC in NaP_i buffer 0.1 M, pH = 5.7.

Determination of the hydrolysis rate constant (k_1) of acetal S3

Acetal **S3** was dissolved in 540 µL of NaP_i buffer (0.1 M, pH = 5.7) containing 60 µL of D_2O to a concentration of 16-17 mM. Percentage degradation of the acetal moiety was calculated by the integration in the ^{1}H NMR spectrum of the peak corresponding to H3 of coumarin attached to the acetal. The hydrolysis reaction obeyed first-order reaction kinetics and hydrolysis rate constants (k_1) were obtained through an exponential fit with good correlation functions ($R^2 > 0.99$, Figure S32).

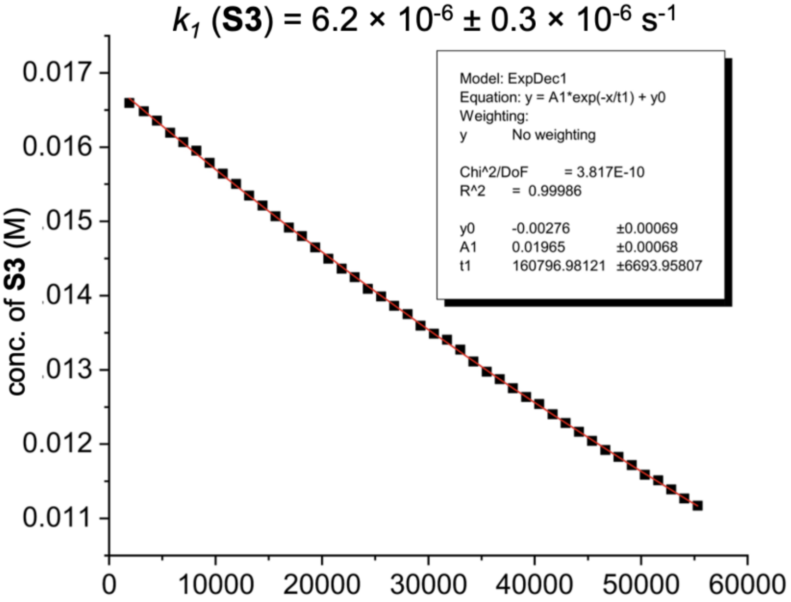

Figure S32. Hydrolysis rate constants (k_1) determined by ^{1}H NMR for acetal **S3** at 37 ºC in NaP_i buffer 0.1 M, pH = 5.7.

Stability of acetal 5 in plasma

Stability of the acetal **5** was assessed in plasma (pH = 7.2, 20% human serum diluted in deionized H_2O) at 37 °C. To this purpose, a stock solution (10 mM) in DMSO was diluted in plasma to a final concentration of 0.6 mM. The solution was incubated at 37 °C and the remaining amount of the acetal was determined by UPLC-MS) using the same conditions as described above for the the hydrolysis rate constant of acetal **5** (Figure S33).

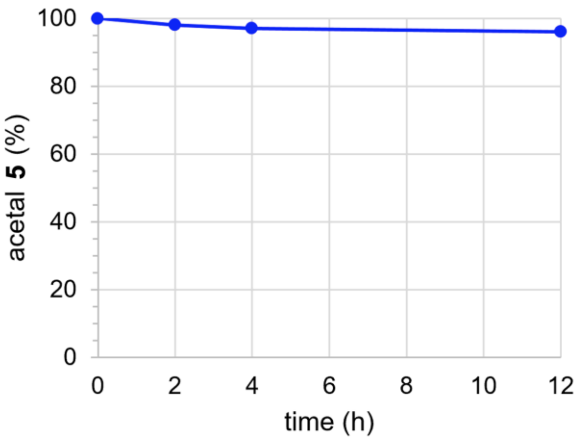

Figure S33. Stability of acetal **5** in plasma (pH = 7.2, 20% human serum diluted in deionized H_2O) tracked by UPLC-MS.

LC–MS method for analysis of protein conjugation

LC–MS was performed on a Water Acquity UPLC system equipped with a single quadrupole mass detector using an Acquity UPLC protein BEH C4 column, 300 Å, (1.7 mm, 2.1 × 50 mm). Solvents A, water with 0.01% formic acid and B, 71% CH_3CN, 29% H_2O and 0.075% HCO_2H were used as the mobile phase at a flow rate of 0.2 mL/min from 0–20 min, and 0.05 mL/min from 20–30 min. The electrospray source was operated with a capillary voltage of 3.0 kV and a cone voltage of 20 V. Nitrogen was used as the desolvation gas at a total flow of 700 L/h. Total mass spectra were reconstructed from the ion series using the MaxEnt algorithm preinstalled on MassLynx software (v. 4.1 from Waters) according to the manufacturer's instructions. To obtain the ion series described, the major peak(s) of the chromatogram were selected for integration and further analysis.

Analysis of protein conjugation by LC–MS

The total ion chromatogram, combined ion series and deconvoluted spectra were measured for the starting material and the product of the bioconjugation reaction. This allowed to monitor progress of the conversion of the non-modified antibody to the conjugated antibody. *Note: Thiomab Tras-LC-V205C and Thiomab Tras-LC-V205C-K207A will be named as IgG and IgG-K207A, respectively.*

Fab-LC-V205C and Fab-LC-V205C-K207A will be named Fab and Fab-K207A, respectively.

Conjugation reactions and characterization of ADCs

Conjugation reaction and characterization of IgG-1

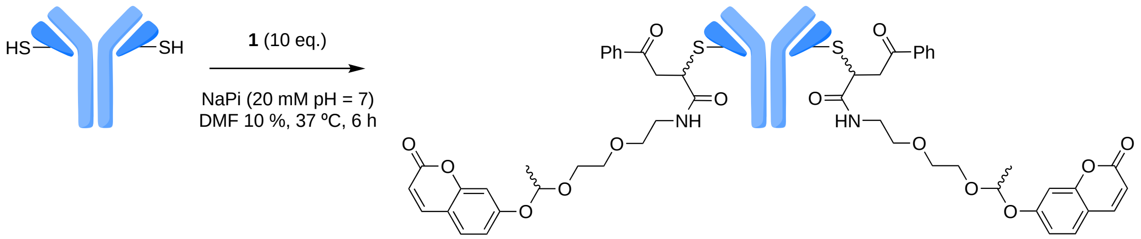

Scheme S8. Conjugation reaction to prepare IgG-**1**.

To an eppendorf containing 23.5 µL of NaP_i (20 mM, pH 7.0) and 2.78 µL of DMF, a 12.5 µL aliquot of a stock solution of IgG (80 µM) was added and the resulting mixture was vortexed for 10 s. Afterwards, 1.2 µL (10 eq.) of an 8.26 mM solution of compound **1** in DMF was added and the reaction mixed for 6 h at 37 °C. Small molecules were removed from the reaction mixture by loading the sample onto a Zeba Spin Desalting Column previously equilibrated with NaP_i (20 mM, pH 7.0). The sample was eluted via centrifugation (2 min, 1500xg). Time points were taken by analysing 10 µL aliquots by LC-MS. Complete conversion was observed after 6 h (calculated mass light chain 23892, observed mass 23897).

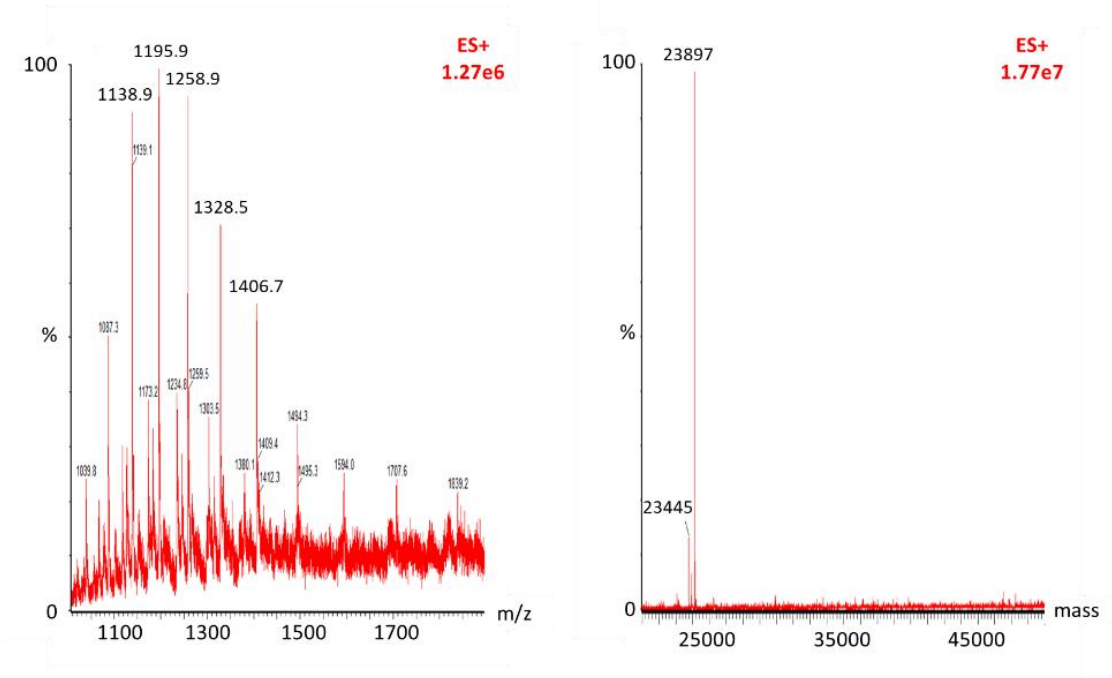

Figure S34. Combined ion series and deconvoluted mass spectra of the light chain of IgG after reaction with compound **1** for 6 h, at pH 7, 37 ºC.

Conjugation reaction and characterization of IgG-K207A-1

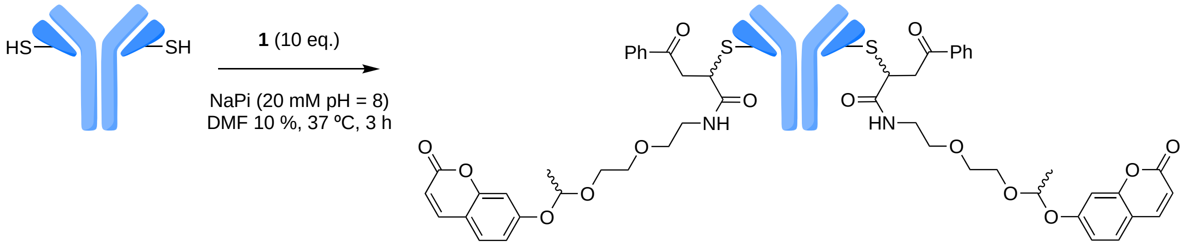

Scheme S9. Conjugation reaction to prepare IgG-K207A-**1**.

To an eppendorf containing 24.75 µL of NaP_i (20 mM, pH 8.0) and 3.64 µL of DMF, a 11.25 µL aliquot of a stock solution of IgG-K207A (64 µM) was added and the resulting mixture was vortexed for 10 s. Afterwards, 0.36 µL (10 eq.) of an 20 mM solution of compound **1** in DMF was added and the reaction mixed for 3 h at 37 °C. Small molecules were removed from the reaction mixture by loading the sample onto a Zeba Spin Desalting Column previously equilibrated with NaP_i (20 mM, pH 8.0). The sample was eluted via centrifugation (2 min, 1500xg). Time points were taken by analysing 10 µL aliquots by LC-MS. Complete conversion was observed after 3 h (calculated mass light chain 23835, observed mass 23844).

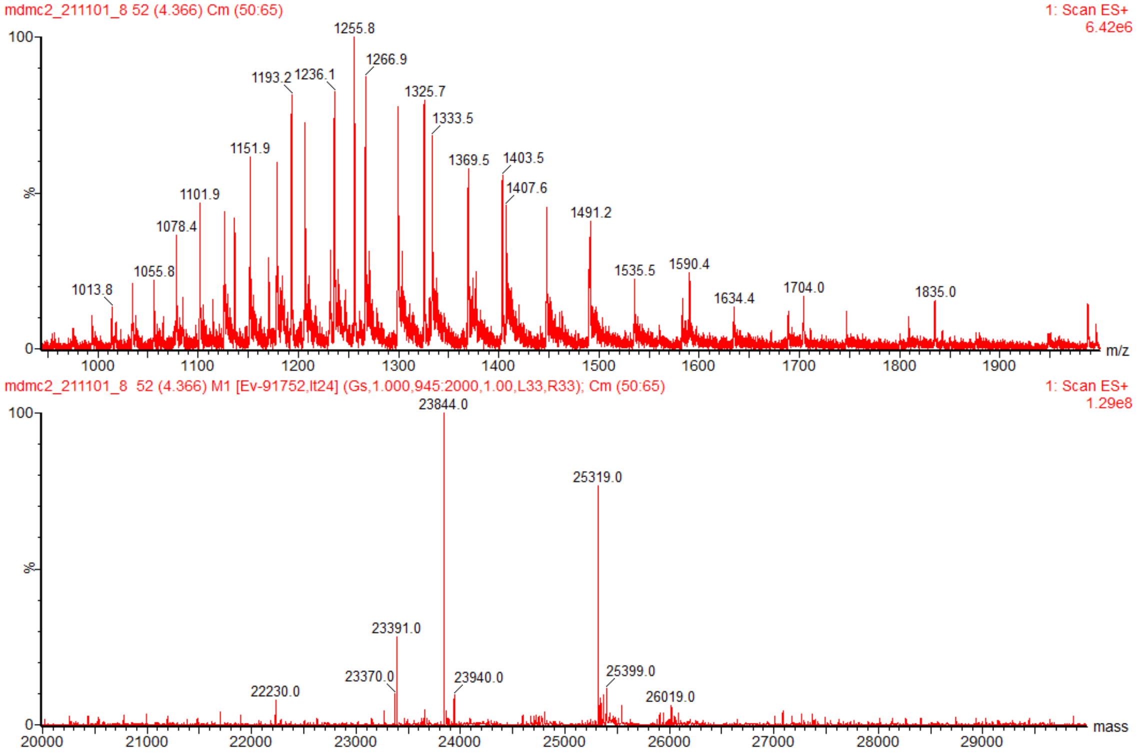

Figure S35. Combined ion series and deconvoluted mass spectra of the light chain of IgG-K207A after reaction with compound **1** for 3 h, at pH 8, 37 ºC.

Conjugation reaction and characterization of IgG-2

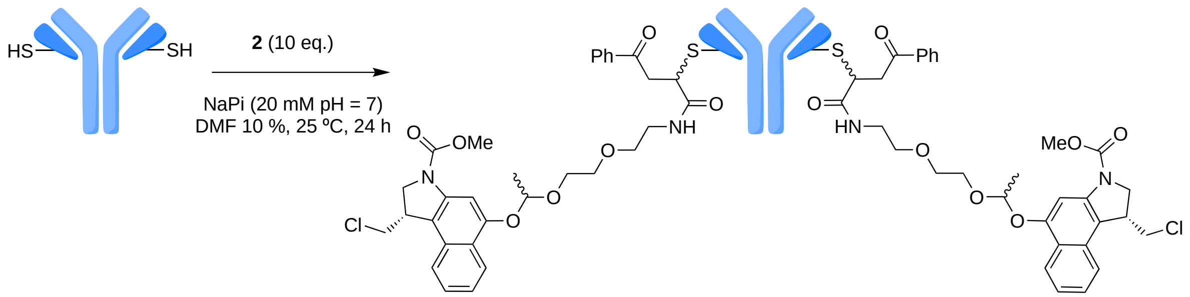

Scheme S10. Conjugation reaction to prepare IgG-**2.**

To an eppendorf containing 17.57 μL of NaP$_i$ (20 mM, pH 7.0) and 3.6 μL of DMF, a 18.43 μL aliquot of a stock solution of IgG (43.4 μM) was added and the resulting mixture was vortexed for 10 s. Afterwards, 0.4 μL (10 eq.) of an 20 mM solution of compound **2** in DMF was added and the reaction mixed for 24 h at 25 ºC. Small molecules were removed from the reaction mixture by loading the sample onto a Zeba Spin Desalting Column previously equilibrated with NaP$_i$ (20 mM, pH 7.0). The sample was eluted via centrifugation (2 min, 1500xg). Time points were taken by analysing 10 μL aliquots by LC-MS. The reaction was stopped after 24 h incubation (calculated mass light chain 24021, observed mass 24030; IgG light chain unmodified calculated mass 23441, observed mass 23449; acetal hydrolysis light chain calculated mass 23704, observed mass 23713).

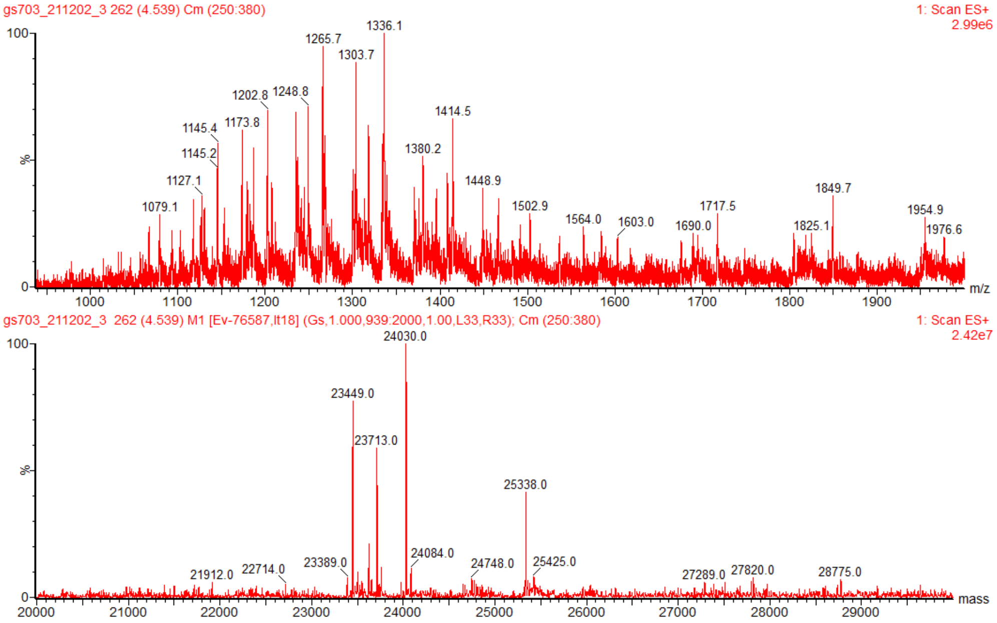

Figure S36. Combined ion series and deconvoluted mass spectra of the light chain of IgG after reaction with compound **2** for 24 h, at pH 7 and 25 ºC.

Conjugation reaction and characterization of IgG-K207A-2

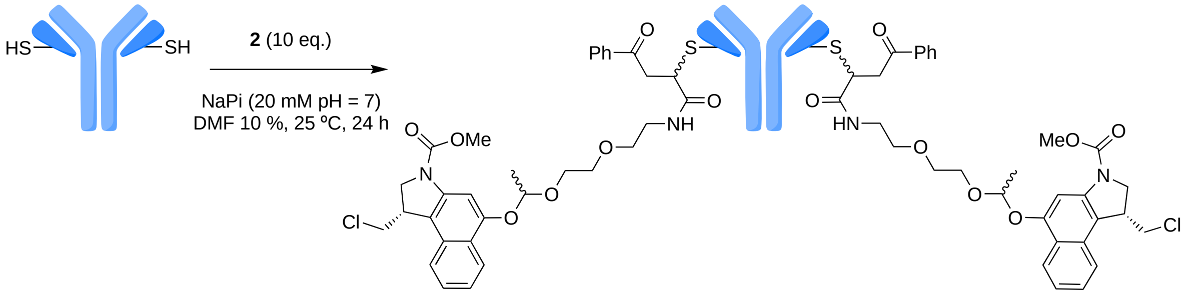

Scheme S11. Conjugation reaction to prepare IgG-K207-**2**.

To an eppendorf containing 21.89 µL of NaP_i (20 mM, pH 7.0) a 14.11 µL aliquot of a stock solution of IgG-V205C/K207A (56.7 µM in NaPi 20 mM, pH 7.0) was added and the resulting mixture was vortexed for 10 s. Afterwards, 0.4 µL (10 eq.) of a 20 mM solution of compound **2** in DMF was added and the reaction mixed for 24 h at 25 ºC. Small molecules were removed from the reaction mixture by loading the sample onto a Zeba Spin Desalting Column previously equilibrated with NaP_i (20 mM, pH 7.0). The sample was eluted via centrifugation (2 min, 1500xg). Time points were taken by analysing 10 µL aliquots by LC-MS. The reaction was stopped after 24 h incubation (calculated mass light chain 23964, observed mass 23973; IgG light chain unmodified calculated mass 23384, observed mass 23390; acetal hydrolysis light chain calculated mass 23647, observed mass 23655).

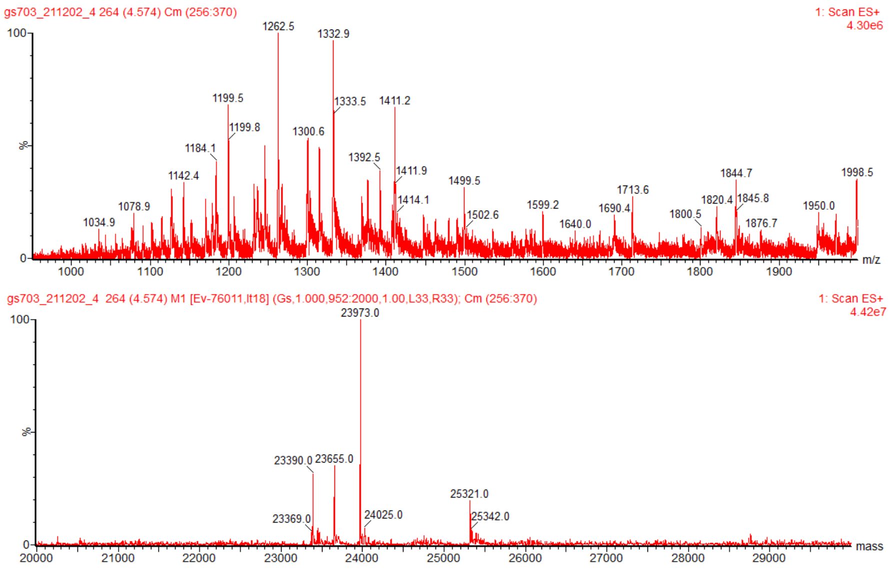

Figure S37. Combined ion series and deconvoluted mass spectra of the light chain of IgG-K207-**2** after reaction with compound **2** for 24 h, at pH 7 and 25 ºC.

Conjugation reaction and characterization of Fab-K207A-2

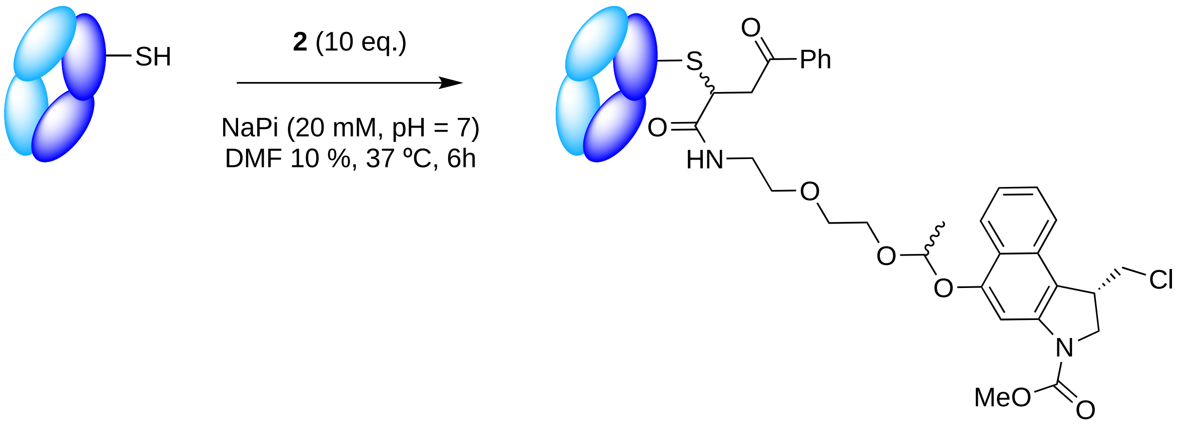

Scheme S12. Conjugation reaction to prepare Fab-K207A-**2**.

To an eppendorf containing 6.4 µL of NaP_i (20 mM, pH 7.0) a 29.6 µL aliquot of a stock solution of Fab-K207A (27 µM in NaP_i 20 mM, pH 7.0) was added and the resulting mixture was vortexed for 10 s. Afterwards, 4 µL (10 eq.) of a 2 mM solution of compound **2** in DMF was added and the reaction mixed for 6 h at 37 ºC. Small molecules were removed from the reaction mixture by loading the sample onto a Zeba Spin Desalting Column previously equilibrated with NaP_i (20 mM, pH 7.0). The sample was eluted via centrifugation (2 min, 1500xg). Time points were taken by analyzing 10 µL aliquots by LC-MS (calculated mass 48197, observed mass 48200; Fab unmodified calculated mass 47161, observed mass 47167; Fab hydrolysis calculated mass 47880, observed mass 47882).

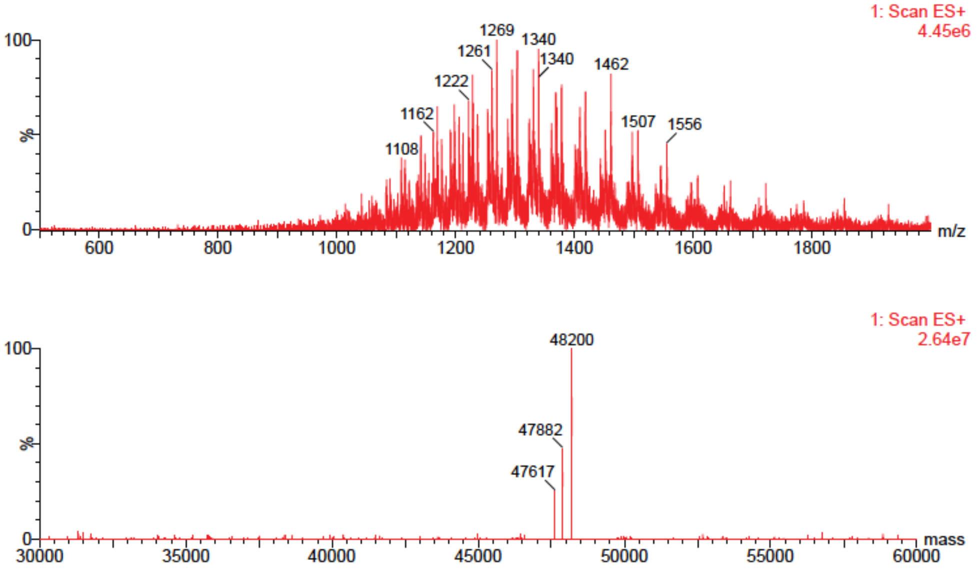

Figure S38. Combined ion series and deconvoluted mass spectra of the Fab-K207A after reaction with compound **2** for 6 h, at pH 7 and 37 ºC.

Conjugation reaction and characterization of IgG-3

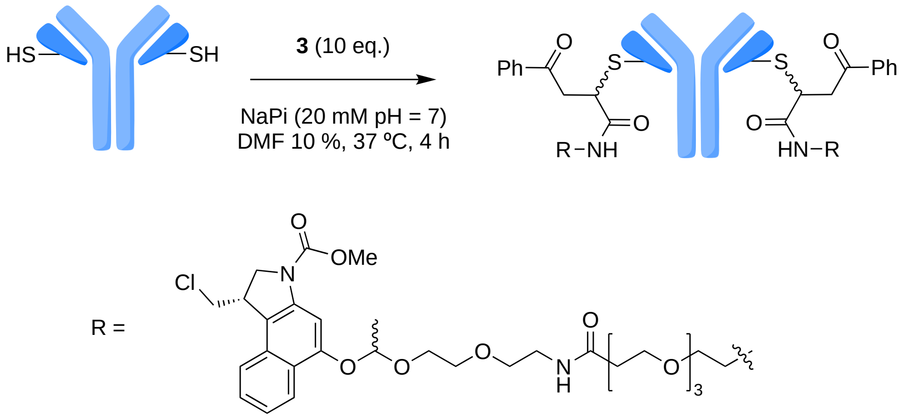

Scheme S13. Conjugation reaction to prepare IgG-**3.**

To an eppendorf containing 26 μL of NaP_i (20 mM, pH 7.0) and 3.6 μL of DMF, a 10 μL aliquot of a stock solution of IgG (80 μM) was added and the resulting mixture was vortexed for 10 s. Afterwards, 0.4 μL (10 eq.) of a 20 mM solution of compound **3** in DMF was added and the reaction mixed for 4 h at 37 ºC. Small molecules were removed from the reaction mixture by loading the sample onto a Zeba Spin Desalting Column previously equilibrated with NaP_i (20 mM, pH 7.0). The sample was eluted via centrifugation (2 min, 1500xg). Time points were taken by analyzing 10 μL aliquots by LC-MS. Complete conversion was observed after 6 h (calculated mass light chain 24224, observed mass 24227).

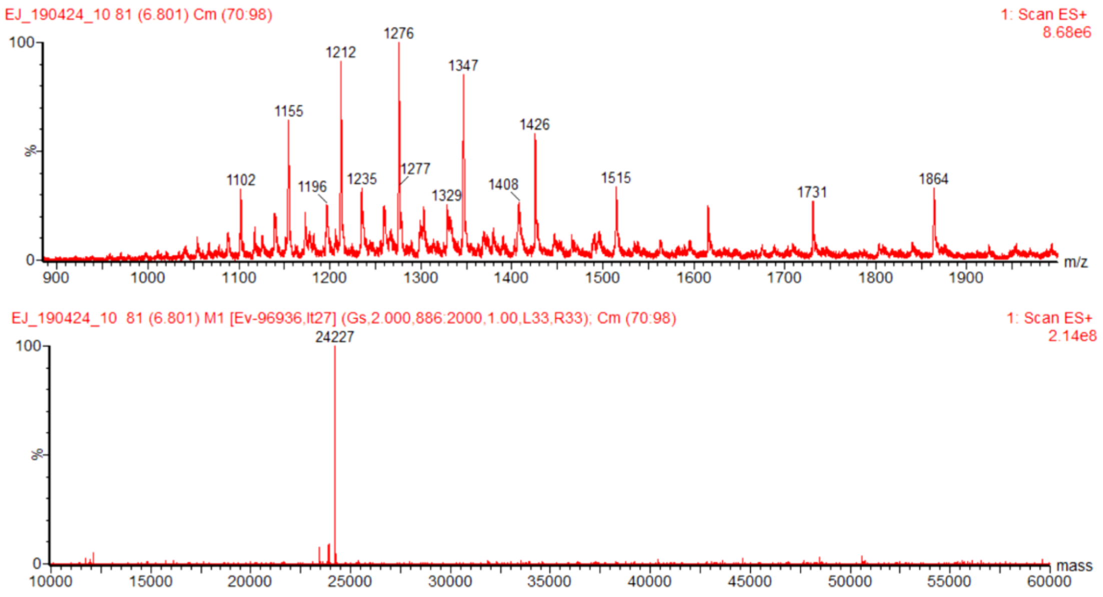

Figure S39. Combined ion series and deconvoluted mass spectra of the light chain of IgG after reaction with compound **3** for 4 h, at pH 7 and 37 ºC.

Conjugation reaction and characterization of Fab-K207A-3

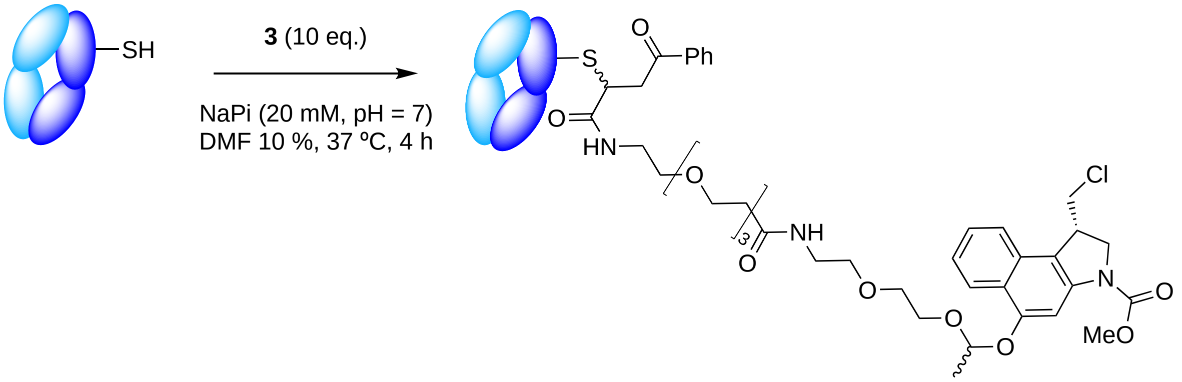

Scheme S14. Conjugation reaction to prepare Fab-K207A-**3.**

To an eppendorf containing 6.4 μL of NaP_i (20 mM, pH 7.0) a 29.6 μL aliquot of a stock solution of Fab-K207A (27 μM in NaPi 20 mM, pH 7.0) was added and the resulting mixture was vortexed for 10 s. Afterwards, 4 μL (10 eq.) of a 2 mM solution of compound **3** in DMF was added and the reaction mixed for 4 h at 37 ºC. Small molecules were removed from the reaction mixture by loading the sample onto a Zeba Spin Desalting Column previously equilibrated with NaP_i (20 mM, pH 7.0). The sample was eluted via centrifugation (2 min, 1500xg). Time points were taken by analysing 10 μL aliquots by LC-MS. Complete conversion was observed after 4 h (calculated mass 48394, observed mass 48404).

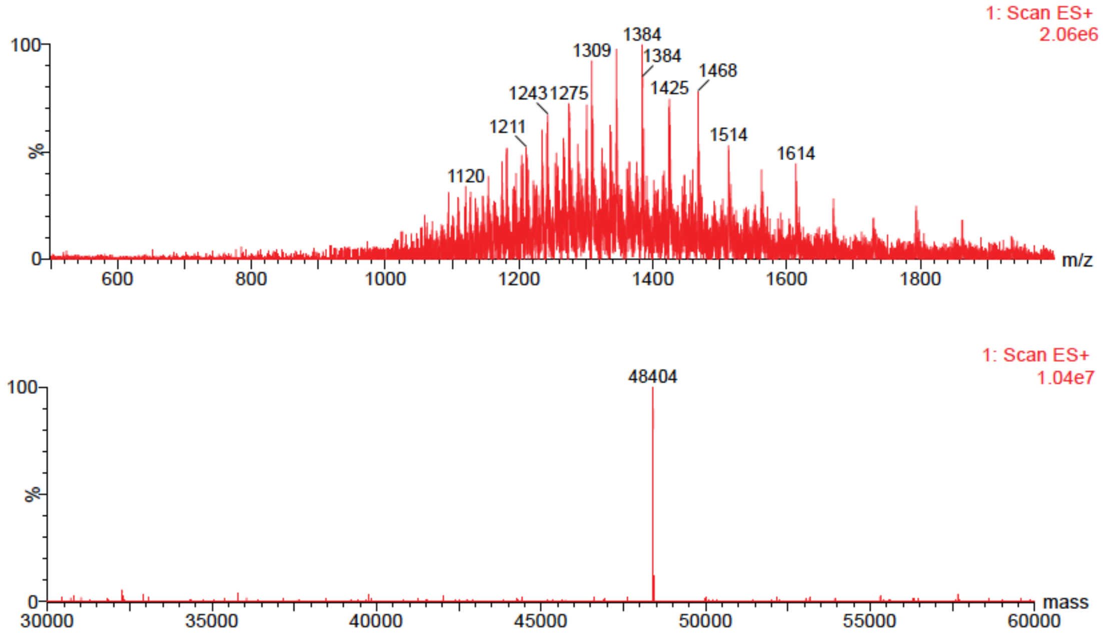

Figure S40. Combined ion series and deconvoluted mass spectra of the Fab-K207A after reaction with compound **3** for 4 h, at pH 7 and 37 ºC.

Conjugation reaction and characterization of IgG-K207A-3

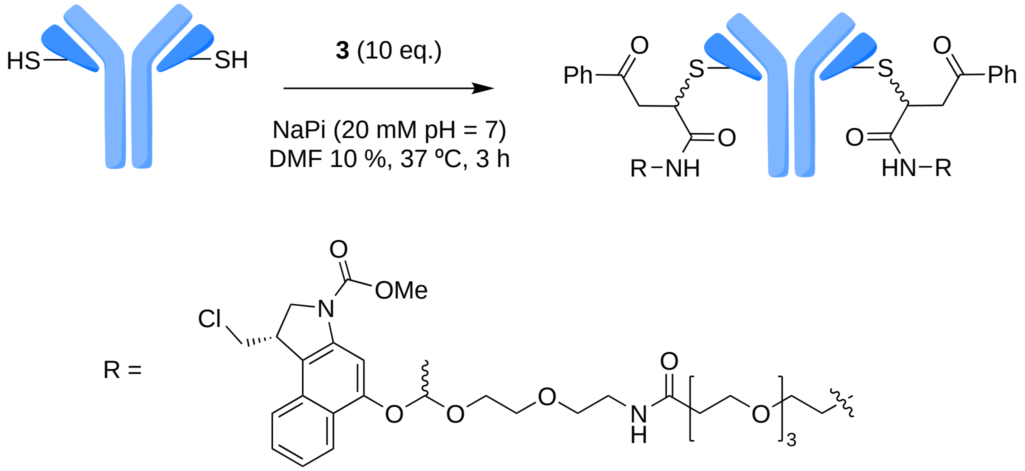

Scheme S15. Conjugation reaction to prepare IgG-K207A-**3.**

To an eppendorf containing 21.5 µL of NaP_i (20 mM, pH 7.0) a 14.5 µL aliquot of a stock solution of IgG-V205C/K207A (55 µM in NaPi 20 mM, pH 7.0) was added and the resulting mixture was vortexed for 10 s. Afterwards, 4 µL (10 eq.) of a 2 mM solution of compound **3** in DMF was added and the reaction mixed for 3 h at 37 ºC. Small molecules were removed from the reaction mixture by loading the sample onto a Zeba Spin Desalting Column previously equilibrated with NaP_i (20 mM, pH 7.0). The sample was eluted via centrifugation (2 min, 1500xg). Time points were taken by analysing 10 µL aliquots by LC-MS. Complete conversion was observed after 3 h (calculated mass light chain 24168, observed mass 24175).

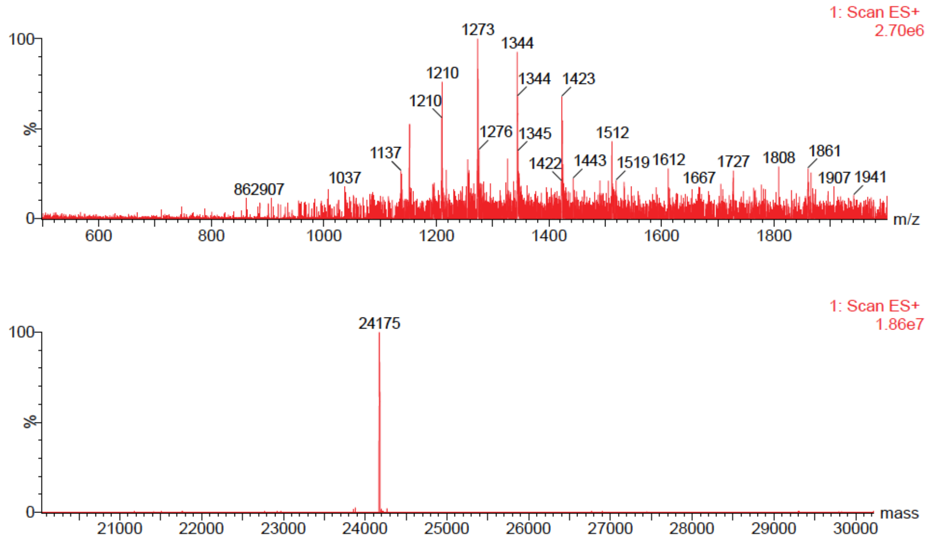

Figure S41. Combined ion series and deconvoluted mass spectra of the IgG-K207A after reaction with compound **3** for 3 h, at pH 7 and 37 ºC.

Conjugation reaction and characterization of IgG-4

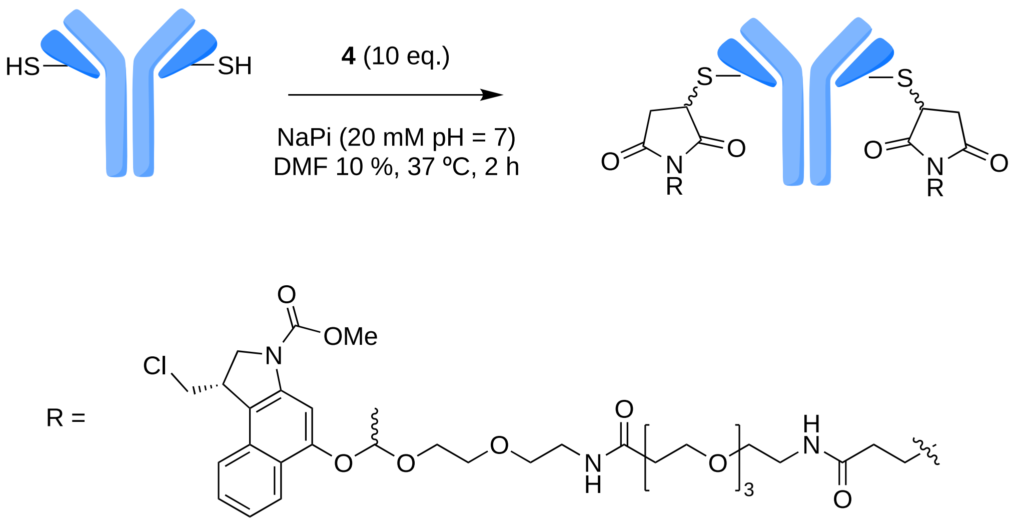

Scheme S16. Conjugation reaction to prepare IgG-**4**.

To an eppendorf containing 123.7 μL of NaP_i (20 mM, pH 7.0) and 15 μL of DMF, a 56.3 μL aliquot of a stock solution of IgG-V205C (71 μM in NaPi 20 mM, pH 7.0) was added and the resulting mixture was vortexed for 10 s. Afterwards, 5 μL (10 eq.) of an 8 mM solution of compound **4** in DMF was added and the reaction mixed for 2 h at 37 ºC. Small molecules were removed from the reaction mixture by loading the sample onto a Zeba Spin Desalting Column previously equilibrated with NaP_i (20 mM, pH 7.0). The sample was eluted via centrifugation (2 min, 1500xg). Time points were taken by analysing 10 μL aliquots by LC-MS. Complete conversion was observed after 2 h (calculated mass light chain 24217, observed mass 24228).

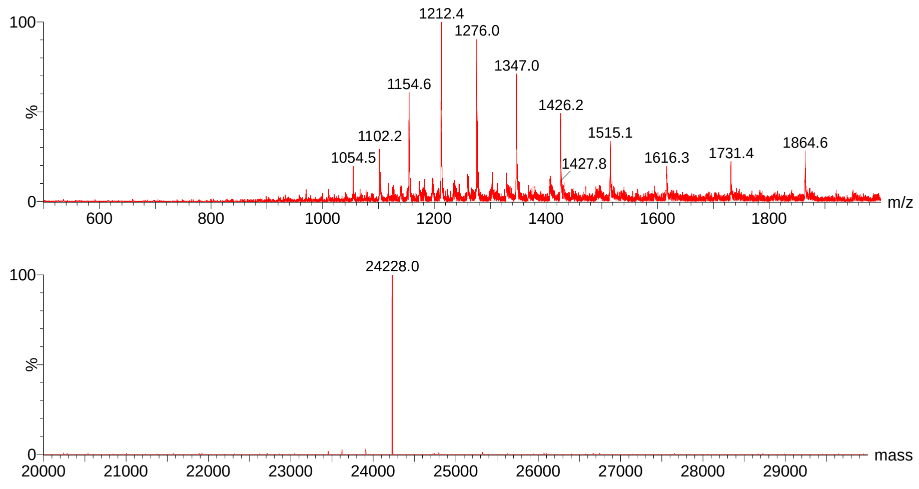

Figure S42. Combined ion series and deconvoluted mass spectra of the IgG after reaction with compound **4** for 2 h, at pH 7 and 37 ºC.

Conjugation reaction and characterization of IgG-K207A-4

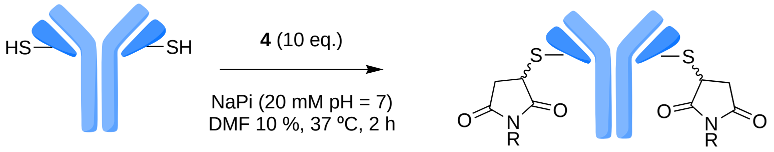

R =

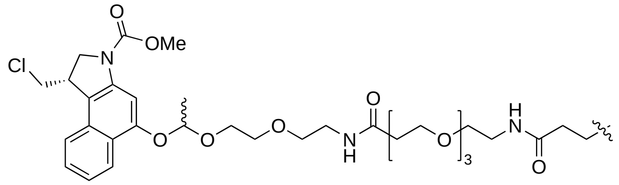

Scheme S17. Conjugation reaction to prepare IgG-K207A-**4**.

To an eppendorf containing 124.4 µL of NaP_i (20 mM, pH 7.0) and 15 µL of DMF, a 55.6 µL aliquot of a stock solution of IgG-V205C/K207A (72 µM in NaPi 20 mM, pH 7.0) was added and the resulting mixture was vortexed for 10 s. Afterwards, 5 µL (10 eq.) of a 8 mM solution of compound **3** in DMF was added and the reaction mixed for 2 h at 37 ºC. Small molecules were removed from the reaction mixture by loading the sample onto a Zeba Spin Desalting Column previously equilibrated with NaP_i (20 mM, pH 7.0). The sample was eluted via centrifugation (2 min, 1500xg). Time points were taken by analysing 10 µL aliquots by LC-MS. Complete conversion was observed after 2 h (calculated mass light chain 24160, observed mass 24170).

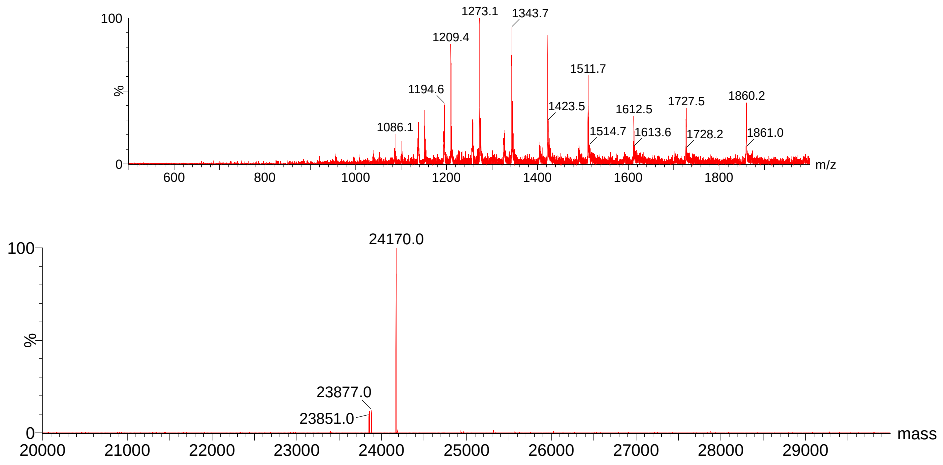

Figure S43. Combined ion series and deconvoluted mass spectra of the IgG V205C/K207A after reaction with compound **4** for 2 h, at pH 7 and 37 ºC. The peak at 23851 corresponds to the acetal hydrolysis and the peak at 23877 to corresponds to the IgG-K207A-**4** conjugate that has undergone both acetal and maleimide hydrolysis.

Stability of ADCs in human plasma and PBS

*Stability of IgG-**1** in human plasma*

A 20 µL aliquot of the IgG-**1** (10 µM) in NaP_i buffer (20 mM, pH 7.0) was thawed. 1 µL of reconstituted human plasma was added at rt and the resulting mixture vortexed for 10 s. The resulting reaction mixture was then stirred at 37 °C. Similarly, to determine the release of coumarin **S1** in acid conditions, buffer was changed to NaP_i (20 mM, pH 5.7) using AmiconR Ultra Centrifuge filters and the concentration adjusted to 10 µM. The resulting reaction mixtures were then stirred at 37 °C. After 24 h, coumarin release was determined by measuring the fluorescence spectrum using a SpectraMax i3x plate reader (λ_{exc}=325 nm for free coumarin **S1**). To avoid interferences with fluorescence, protein content was precipitated with 3-fold excess methanol, separated by centrifugation for 5 min at 14000 rcf and the fluorescence spectrum of **S1** in solution was recorded (Figure 3c in the main text).

*Stability of bioconjugate IgG-**4** in PBS*

A 40 µL aliquot of IgG-**4** (19 µM) in PBS was thawed. The solution was then incubated at 37 °C. After 24 h, a 10 µL aliquot of the reaction mixture was analysed by LC–MS.

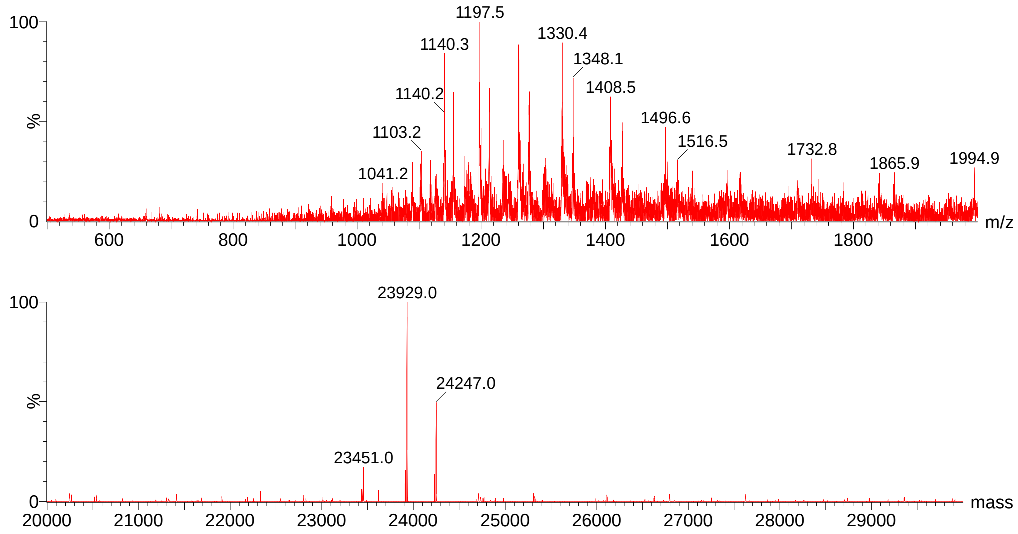

Figure S44. Combined ion series and deconvoluted mass spectra of IgG-**4** in PBS after 24 h (IgG light chain calculated mass 23441, observed mass 23451; IgG-**4** light chain acetal hydrolysis and maleimide hydrolysis calculated mass 23918, observed mass 23929; IgG-**4** light chain maleimide hydrolysis calculated mass 24235, observed mass 24247).

*Stability of bioconjugate IgG-K207A-**4** in PBS*

A 40 μL aliquot of IgG-K207A-4 (19 μM) in PBS was thawed. Then, the solution was incubated at 37 °C. After 24 h, a 10 μL aliquot of the reaction mixture was analyzed by LC–MS.

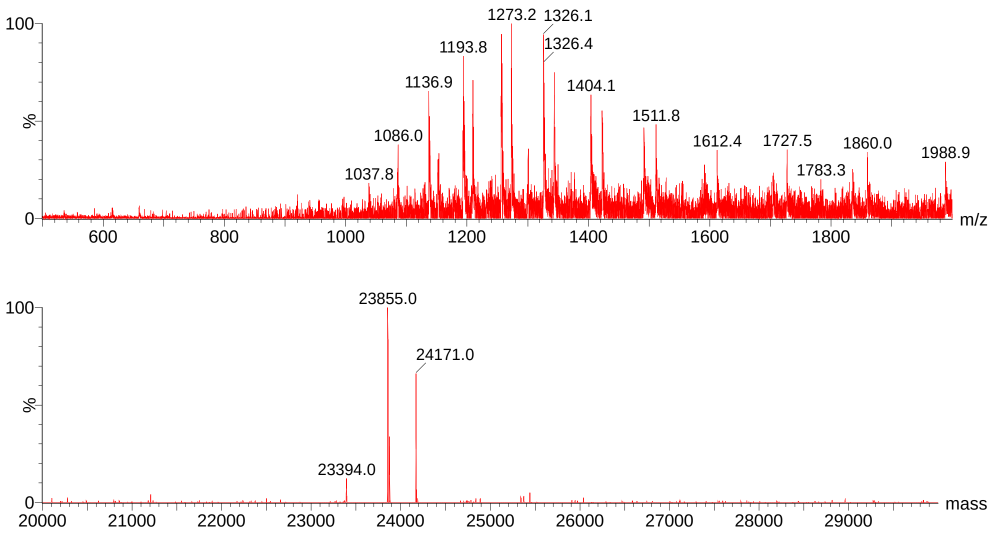

Figure S45. Combined ion series and deconvoluted mass spectra of IgG-K207A-**4** in PBS after 24 h (IgG light chain calculated mass 23384, observed mass 23394; IgG-K205A-**4** light chain acetal hydrolysis calculated mass 23843, observed mass 23855; IgG-K207A-**4** light chain acetal hydrolysis and maleimide hydrolysis calculated mass 23861 observed mass 23875; IgG-K207A-4 light chain calculated mass 24160, observed mass 24171).

Cell culture conditions

SKBR3 (Her2 positive) and MDA-MB 231 cells (Her2 negative) were grown using 1x DMEM (Dulbecco's modified Eagle medium) with sodium pyruvate and without L-Glutamine (Invitrogen, Life Technologies) supplemented with 10% heat-inactivated fetal bovine serum (FBS) (Gibco, Life Technologies), 1x MEM NEAA (Gibco, Life Technologies) ,1x GlutaMAX (Gibco, Life Technologies), 200 units/mL penicillin and 200 µg/mL streptomycin (Gibco, Life Technologies) and 10 mM HEPES (Gibco, Life Technologies).

Flow cytometry assays

Surface staining of Her2 antigen on SKBR3 and MDA-MB 231 cells.

Cells were cultured routinely as mentioned in previous section. Cells were detached and stained for the surface antigen Her2. In brief, cells were harvested with Accutase Cell Detachment Solution (BD Biosciences), collected in FACS buffer (3% FBS, 0.1% sodium azide, in PBS), and incubated with Thiomab (IgG) and K207A-mutated Thiomab (IgG-K207A) conjugated with acetals **1**, **2**, **3**, and **4** at 4 °C for 1 h. Cells were then washed with ice-cold FACS buffer, incubated with a commercial AF488-conjugated F(ab')2-goat anti-human IgG FC secondary antibody (ThermoFisher Scientific) for 30 min. in the dark, washed again and fixed with 2% PFA in PBS for 10 min. in the dark. Samples were acquired using a CytoFLEX flow cytometer (Beckman Coulter) with a 488 nm laser and a 525/40 band-pass filter. Data were analyzed using FlowJo software, only single cell data are shown.

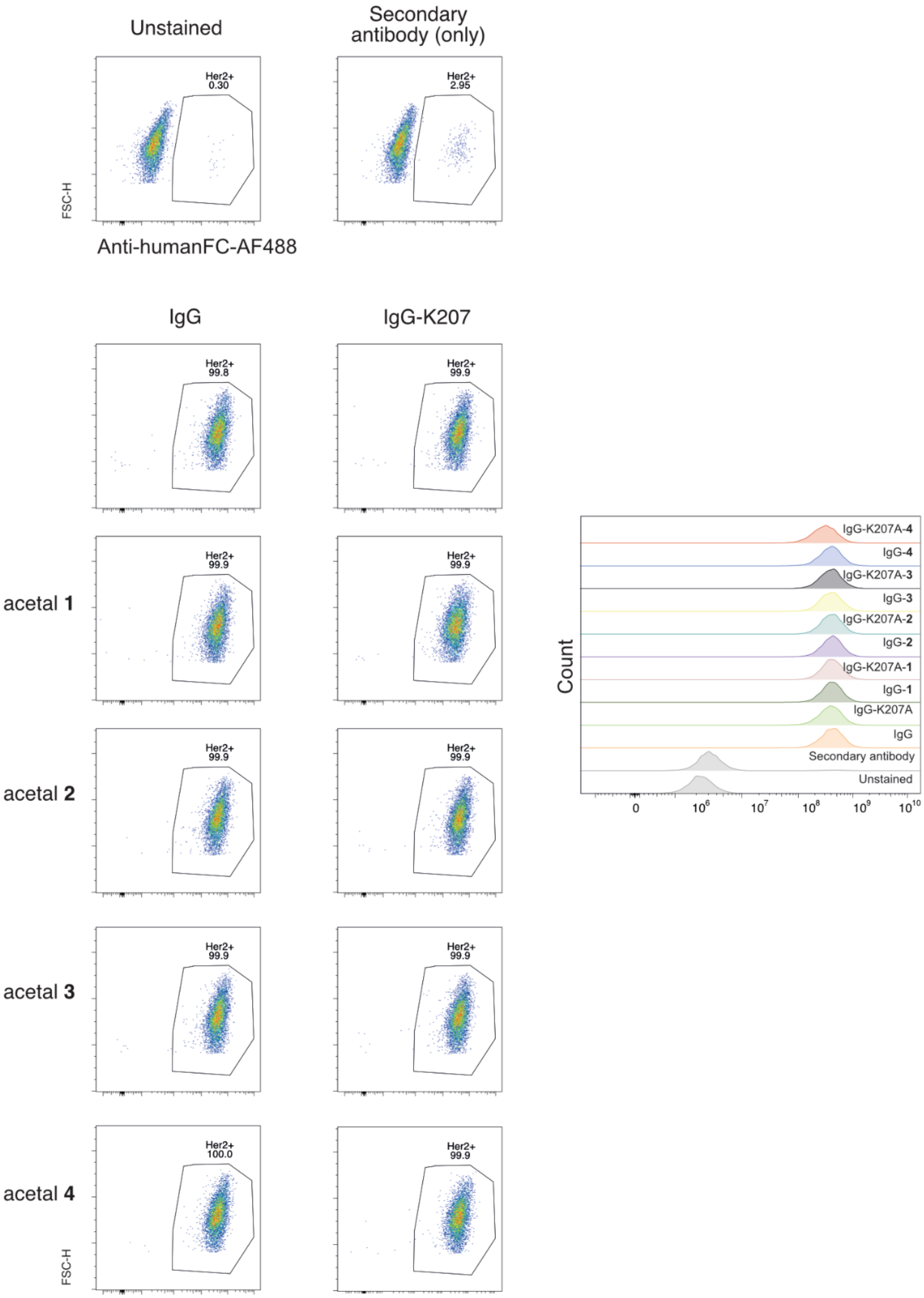

Figure S46. Flow cytometry plots obtained by fluorescence activated cell sorting (FACS) with Her2 expressing cells (SKBR3 cell line).

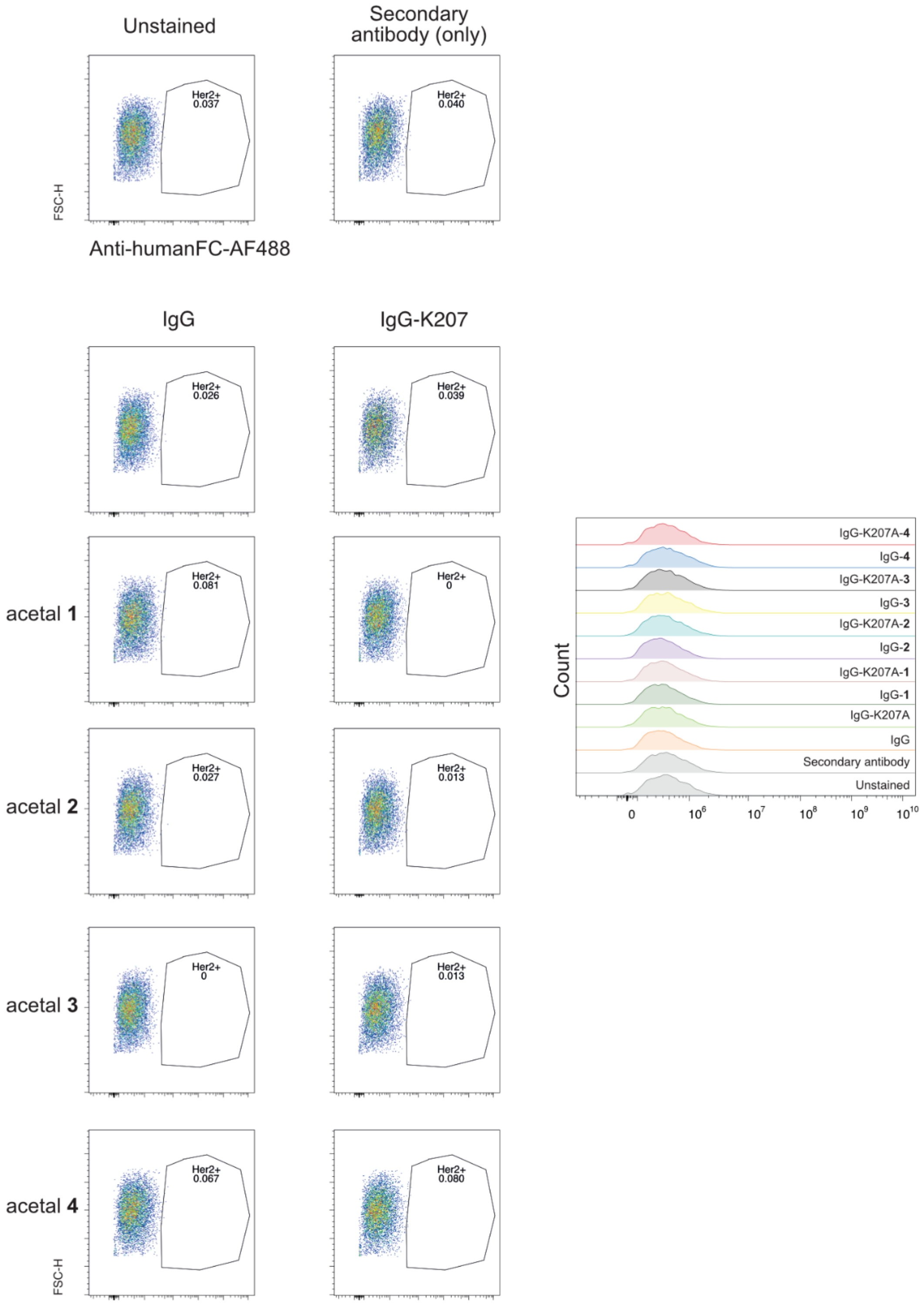

Figure S47. Flow cytometry plots obtained by fluorescence activated cell sorting (FACS) with MDA-MB 231 cell line as a negative control.

Biolayer interferometry

Affinity assays were performed using an Octet Red Instrument (fortéBIO). Ligand immobilization, binding reactions and washes were prepared in wells of black polypropylene 96-well microplates. Biotinylated human Her2 protein (SinoBiological) at 100 nM was immobilized on Streptavidin (SA) Biosensors in PBS pH 7.4 with 0.1% Tween-20 at 25 °C during 70 s. Binding analysis was carried out at 25 °C, 1,000 r.p.m. in PBS pH 7.4 0.1% Tween-20, with a 300 s of association followed by a 450 s of dissociation. 20 nM, 10 nM and 2.5 nM concentration of the different conjugates were used to obtain the binding curves. Data were analysed using Data Analysis HT 11.1 (fortéBIO) software. Binding was fitted to a 1:1 binding model with local partial fitting and dissociation baseline to zero.

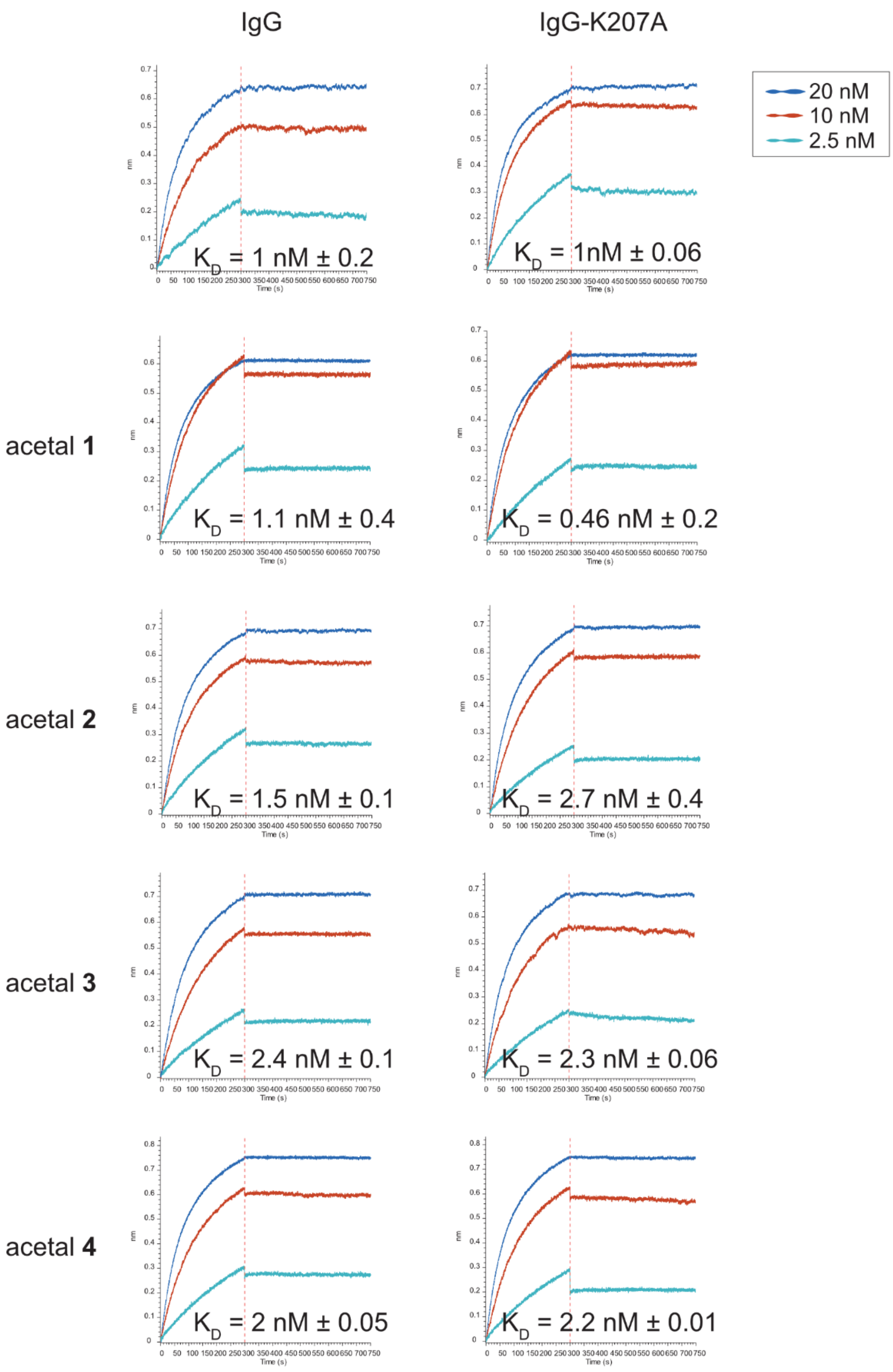

Figure S48. K_D constants derived from BLI experiments for the conjugates studied in this work with with Her2 expressing cells (SKBR3 cell line).

Molecular dynamics (MD) simulations

The crystal structure of the Fab of Trastuzumab (PDB id = 1N8Z)[17] was used as starting coordinates for the antibody. All possible diastereomers of conjugates Fab-**1**, Fab-**2**, Fab-K207R-**2**, Fab-K207H-**2**, Fab-K207D-**2**, Fab-K207E-**2** were considered in the MD simulations. The *R* configuration at both stereocenters of the linker was consider in the calculations Fab-**3**, Fab-**4**, Fab-K207R-**4**, Fab-K207H-**4**, Fab-K207D-**4** and Fab-K207E-**4**. The protonated side chain of Arg and His was used in the simulations. In contrast, the carboxylate form of the side chains of Asp and Glu acids was considered for these calculations. MD simulations were performed with AMBER 18 package,[10] implemented with ff14SB,[18] and GAFF[19] force fields. Parameters of the unnatural residues were generated with the antechamber module of AMBER with partial charges set to fit the electrostatic potential generated with HF/6-31G(d) by RESP.[20] The charges were calculated according to the Merz-Singh-Kollman scheme using Gaussian 16[2] Each conjugate was immersed in a water box with a 10 Å buffer of TIP3P water molecules.[21] The system was neutralized by adding explicit counter ions. A two-stage geometry optimization approach was performed. The first stage minimizes only the positions of solvent molecules and ions, and the second stage is an unrestrained minimization of all the atoms in the simulation cell. The systems were then heated by incrementing the temperature from 0 to 300 K under a constant pressure of 1 atm and periodic boundary conditions. Harmonic restraints of 10 kcal/mol were applied to the solute, and the Andersen temperature coupling scheme[22] was used to control and equalize the temperature. The time step was kept at 1 fs during the heating stages, allowing potential inhomogeneities to self-adjust. Hydrogen atoms were kept fixed through the simulations using the SHAKE algorithm.[23] Long-range electrostatic effects were modelled using the particle-mesh-Ewald method.[16] An 8 Å cutoff was applied to Lennard-Jones interactions. Each system was equilibrated for 2 ns with a 2-fs time step at a constant volume and temperature of 300 K. Production trajectories were then run for additional 500 ns under the same simulation conditions.

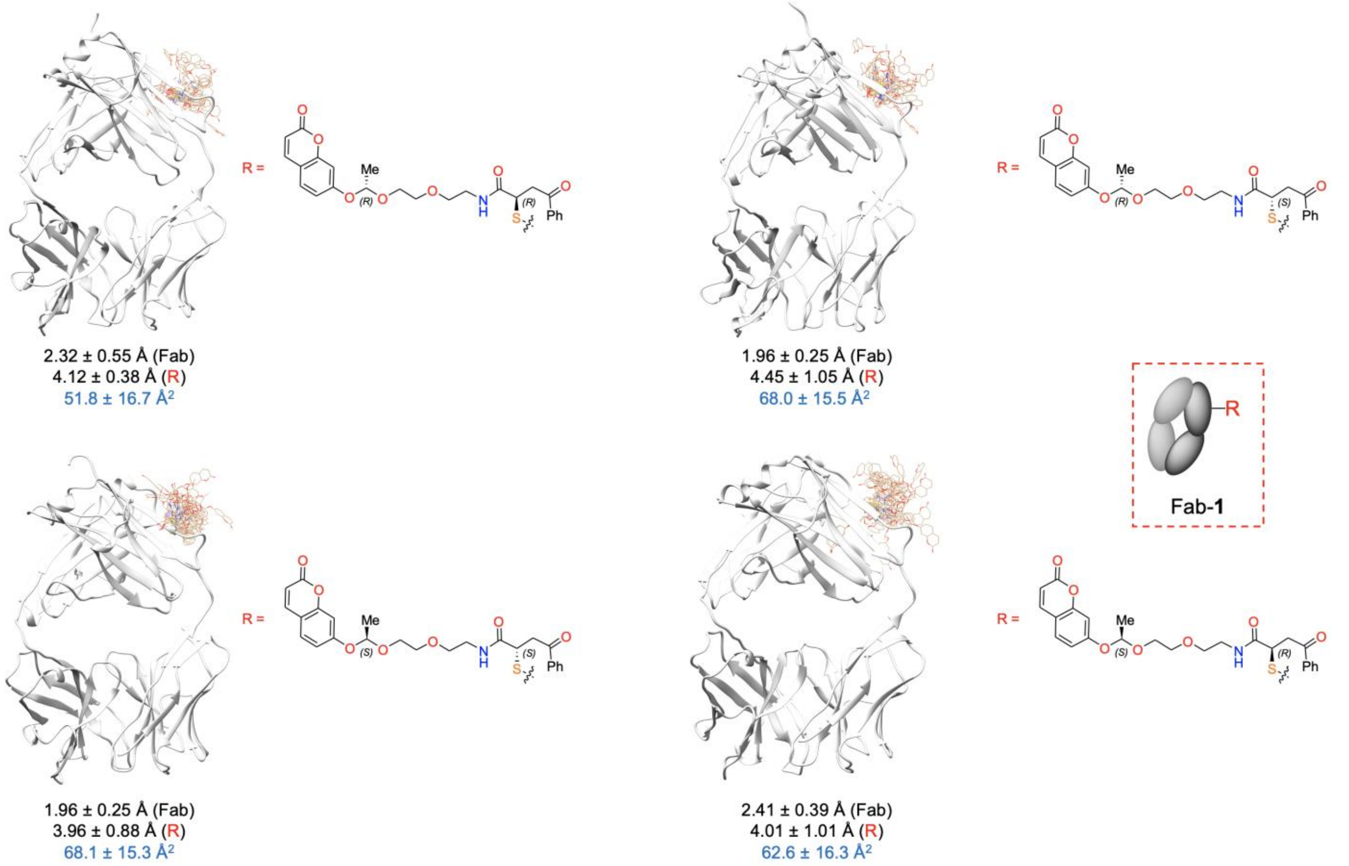

Figure S49. Structural ensembles obtained from 500 ns MD simulations performed on all possible diastereomers resulting from the conjugation of acetal **1** to Fab fragment of Trastuzumab (conjugate: Fab-**1**; Fab PDB entry: 1N8Z). The numbers indicate the root-mean-square deviation of the Fab fragment (backbone) and R group (heavy atoms) with respect to the starting structure. The solvent accessible surface area values obtained from the simulations for the acetal group are shown in blue. Only the first frame of the Fab is shown for clarity.

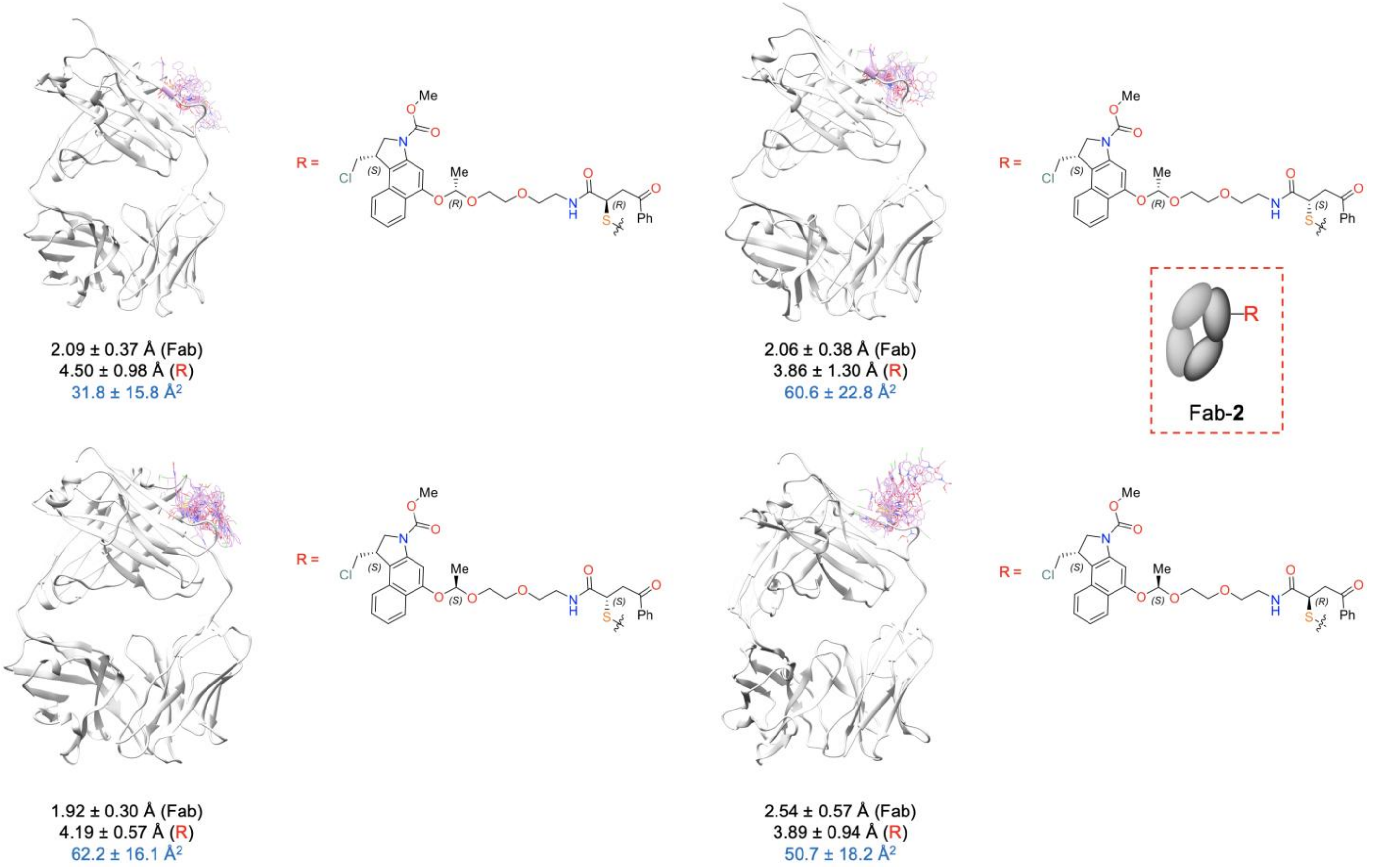

Figure S50. Structural ensembles obtained from 500 ns MD simulations performed on all possible diastereomers resulting from the conjugation of acetal **2** to Fab fragment of Trastuzumab (conjugate: Fab-**2**; Fab PDB entry: 1N8Z). The numbers indicate the root-mean-square deviation of the Fab fragment (backbone) and R group (heavy atoms) with respect to the starting structure. The solvent accessible surface area values obtained from the simulations for the acetal group are shown in blue. Only the first frame of the Fab is shown for clarity.

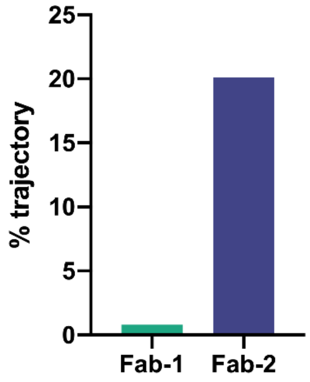

Figure S51. Percentage of the total trajectory time of 500 ns MD simulations on Fab-**1** and Fab-2 where the distance Nε (Lys-207) – C (acetal group) ≤ 4.5 Å.

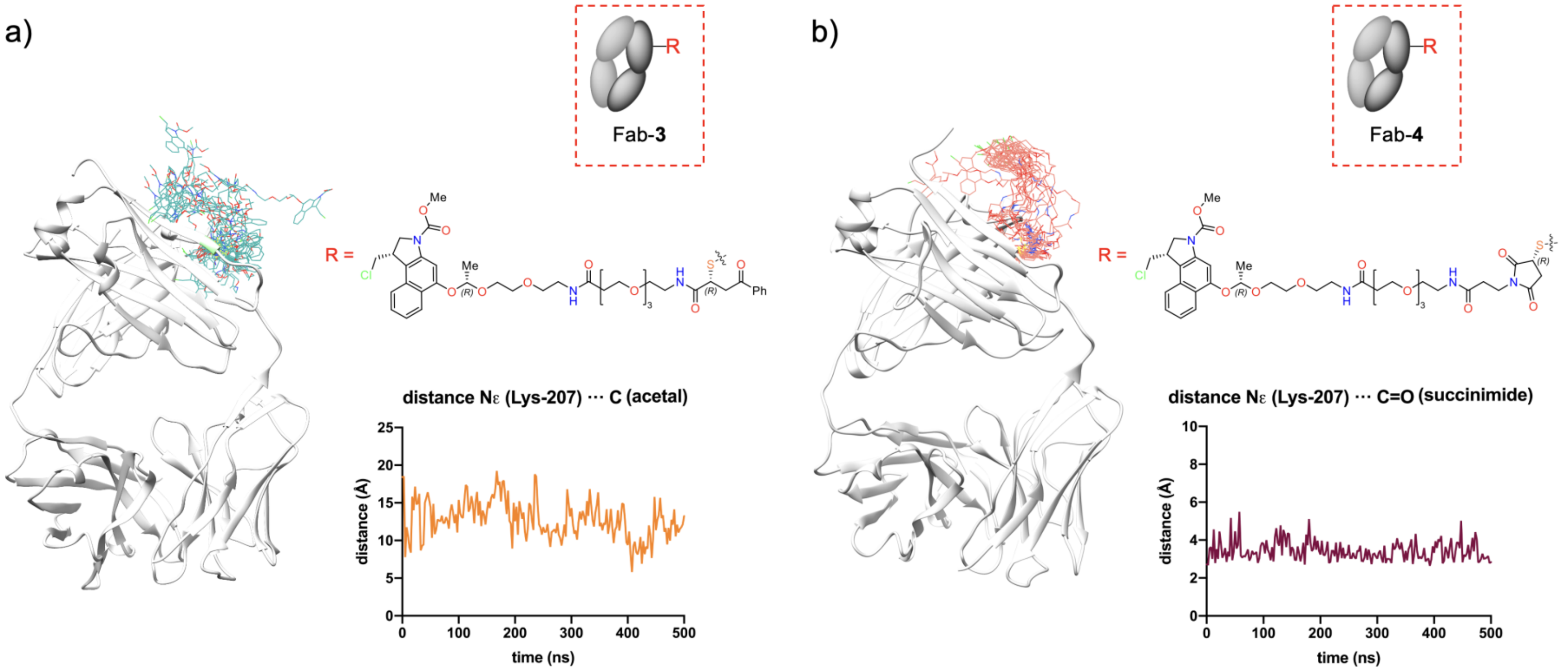

Figure S52. (a) Evolution of the distances between the acetal group and Nε of Lys-207 along the 500 ns MD simulations for Fab-**3**. The structural ensemble of the MD simulations is also shown. The carbon atoms of residue **3** are in green. Only the first frame of the Fab is shown for clarity. (b) Evolution of the distances between the Nε of Lys-207 and the oxygen of the carbonyl group of the succinimide ring and along the 500 ns MD simulations for Fab-**3**. The structural ensemble of the MD simulations is also shown. The carbon atoms of residue **4** are in red. Only the first frame of the Fab is shown for clarity. The *R* configuration at both stereocenters of the linker was consider in these calculations.

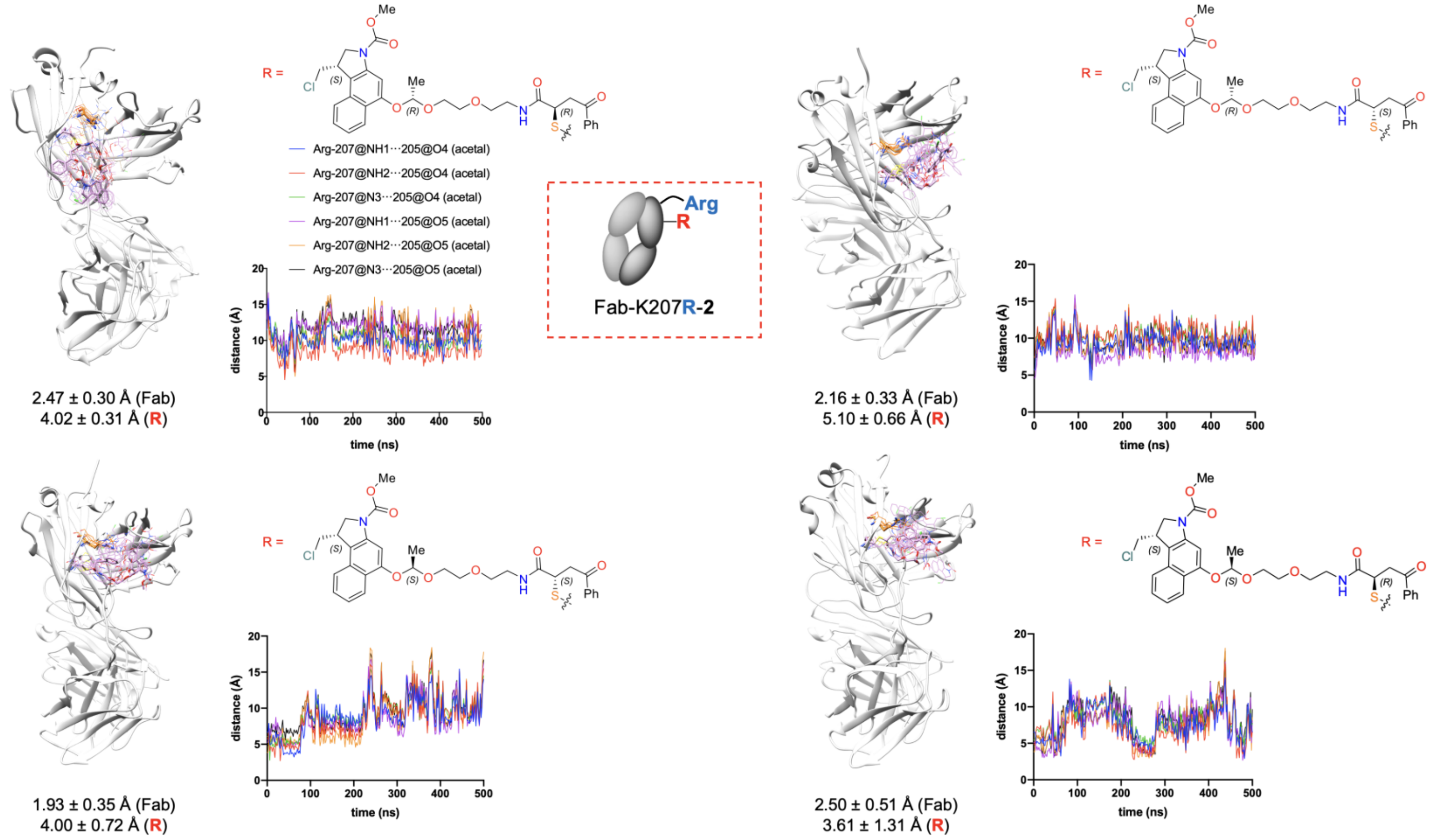

Figure S53. Structural ensembles obtained from 500 ns MD simulations performed on all possible diastereomers resulting from the conjugation of acetal **2** to mutant Fab-K207**R**, together with the root-mean-square deviation of the Fab fragment (backbone) and R group (heavy atoms) with respect to the starting structure. Only the first frame of the Fab is shown for clarity. The evolution of the distance between the side chain of Arg-207 and the oxygen of the acetal group along the 500 ns MD simulations for the conjugates is also shown.

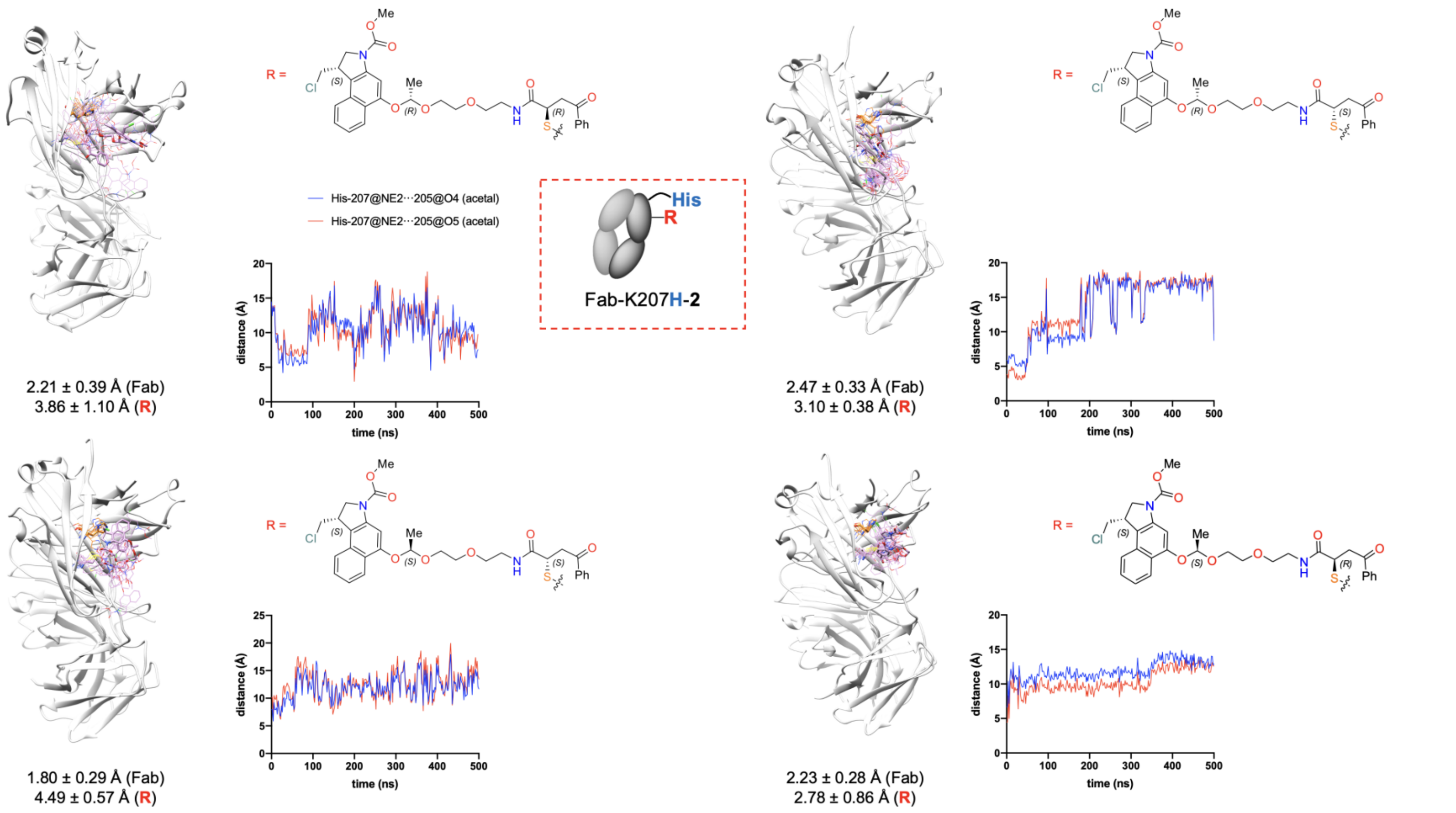

Figure S54. Structural ensembles obtained from 500 ns MD simulations performed on all possible diastereomers resulting from the conjugation of acetal **2** to mutant Fab-K207**H**, together with the root-mean-square deviation of the Fab fragment (backbone) and R group (heavy atoms) with respect to the starting structure. Only the first frame of the Fab is shown for clarity. The evolution of the distance between the side chain of His-207 and the oxygen of the acetal group along the 500 ns MD simulations for the conjugates is also shown.

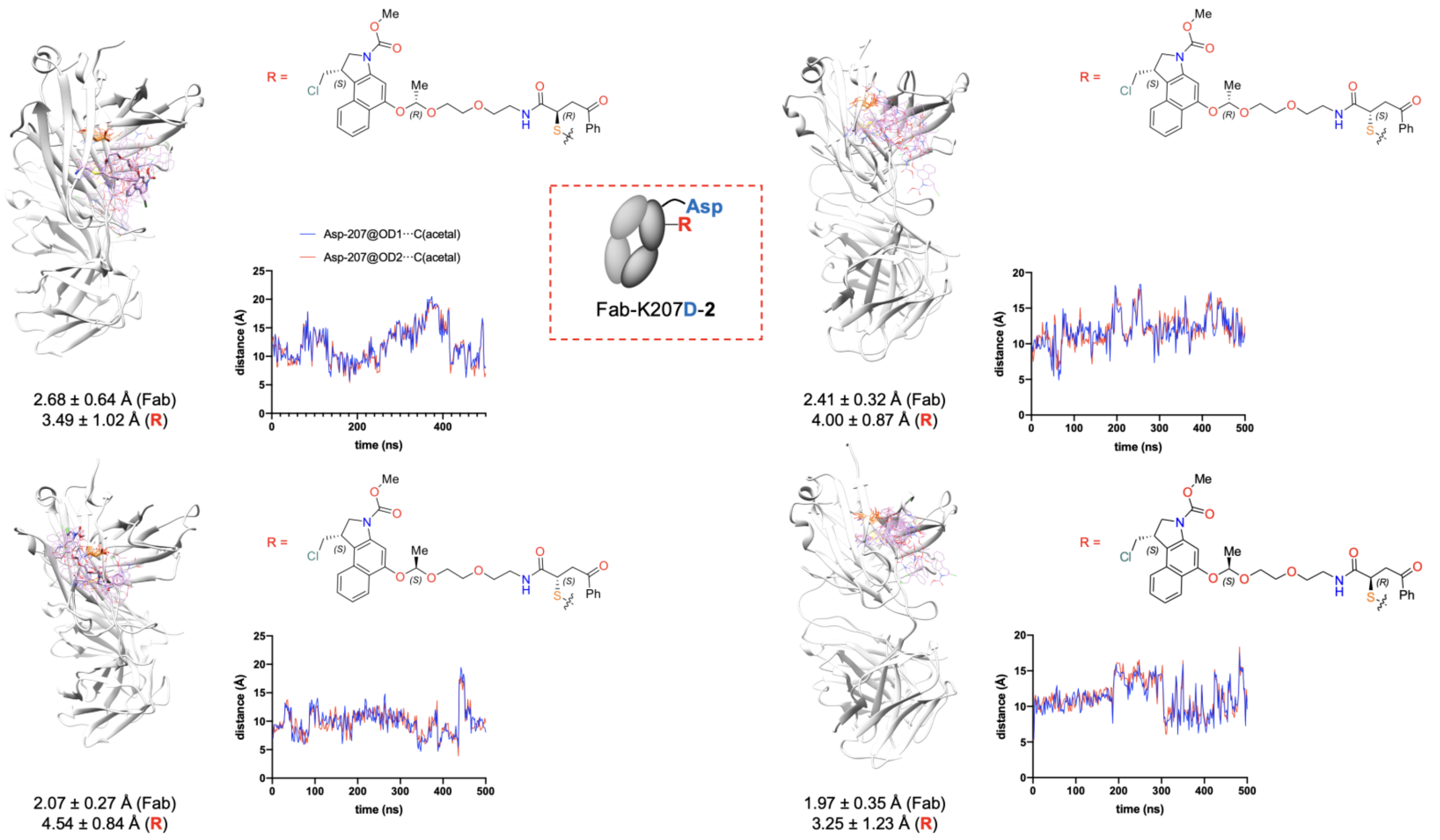

Figure S55. Structural ensembles obtained from 500 ns MD simulations performed on all possible diastereomers resulting from the conjugation of acetal **2** to mutant Fab-K207D, together with the root-mean-square deviation of the Fab fragment (backbone) and R group (heavy atoms) with respect to the starting structure. Only the first frame of the Fab is shown for clarity. The evolution of the distance between the side chain of Asp-207 and the carbon of the acetal group along the 500 ns MD simulations for the conjugates is also shown.

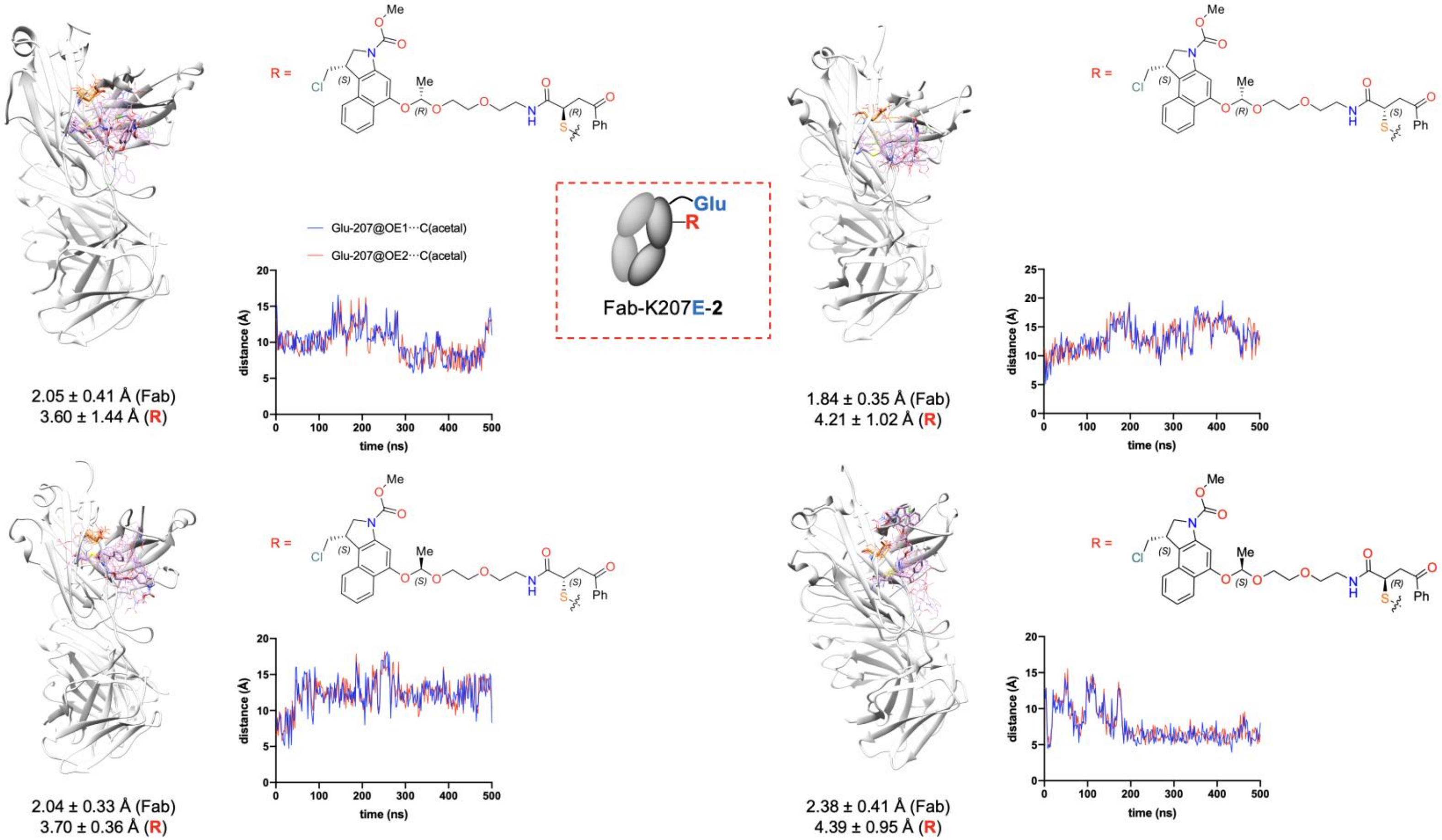

Figure S56. Structural ensembles obtained from 500 ns MD simulations performed on all possible diastereomers resulting from the conjugation of acetal **2** to mutant Fab-K207**E**, together with the root-mean-square deviation of the Fab fragment (backbone) and R group (heavy atoms) with respect to the starting structure. Only the first frame of the Fab is shown for clarity. The evolution of the distance between the side chain of Glu-207 and the carbon of the acetal group along the 500 ns MD simulations for the conjugates is also shown.

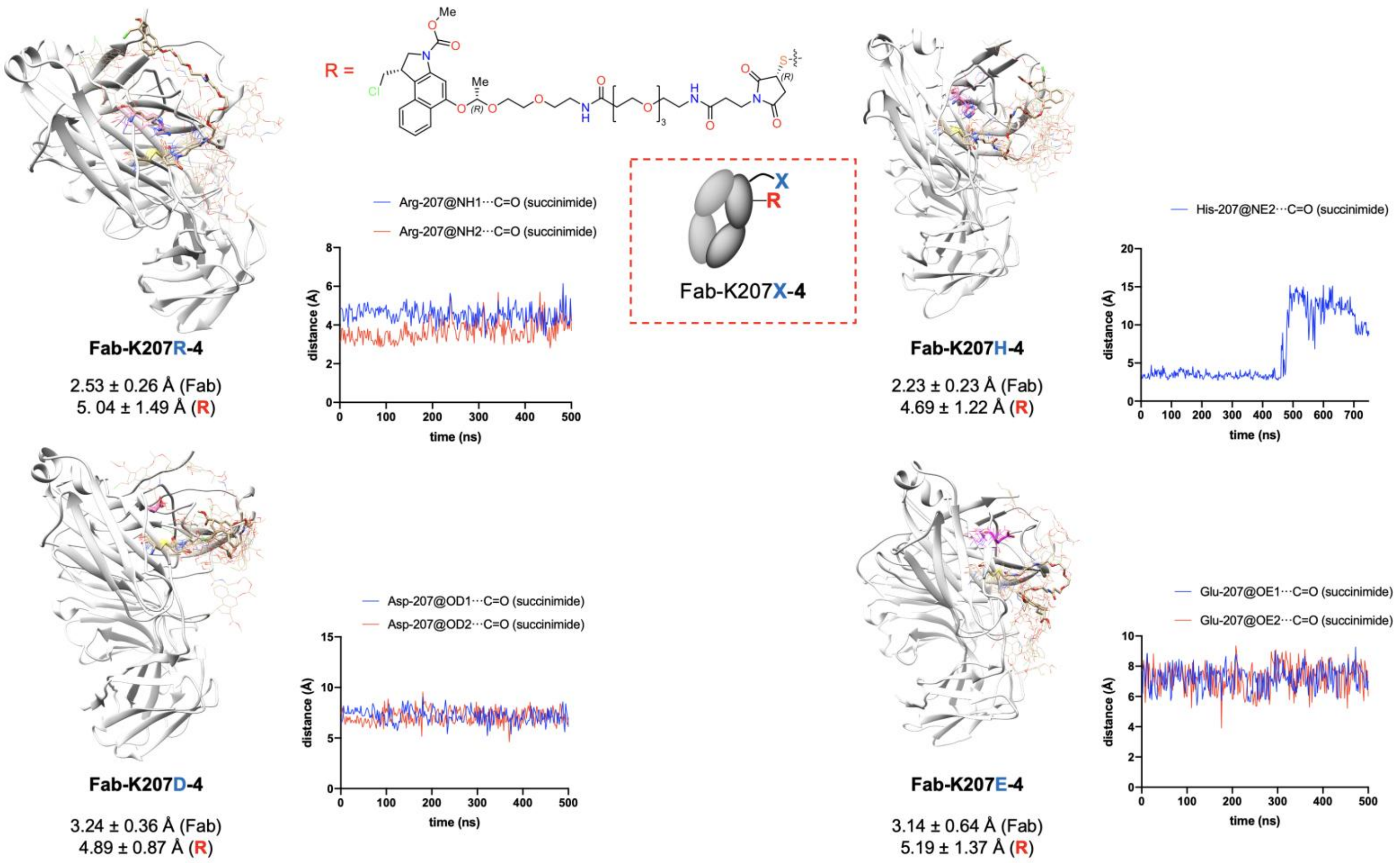

Figure S57. Structural ensembles obtained from 500 ns MD simulations performed on Fab-K20**R**-**4**, Fab-K20**H**-**4**, Fab-K20**D**-**4**, Fab-K20**E**-**4**, together with the root-mean-square deviation of the Fab fragment (backbone) and R group (heavy atoms) with respect to the starting structure. The *R* configuration at both stereocenters of the linker was consider in these calculations. Only the first frame of the Fab is shown for clarity. The evolution of the distance between the side chain of Arg-207, His-207, Asp-207 and Glu-207 oxygen of the carbonyl group of the succinimide ring and along the 500 ns MD simulations is also shown.

Production and characterization of IgG-K207A and Fab-K207A

The human kappa region with the mutations V205C, K207A were produced as a string by GeneArt and cloned into the corresponding pEU vector containing the Trastuzumab light chain variable gene using Gibson Assembly (NEB). IgG was expressed in CHO cells and purified as described previously.[24]
Specifically, IgG containing supernatants were cleared by centrifugation and filtration and purified on at 5 ml HiTrap MapSelecT SuRe columns loading either 100 ml or 300 ml supernatant, washed with 20 column volumes PBS and eluted with 0.1 M glycine pH 2.7. Eluted fractions (Figure S58) were pooled, and buffer exchanged to PBS using PD10 columns. Fab fragments from the V205C, K207A variant was prepared by digesting with papain following manufactures instruction. Fc and partial digested IgG was depleted from the reaction in a "flow through" chromatographic step prior to capture on a CH1 columns and elution with 25 mM NaAce pH 3.6 (Figure S58).

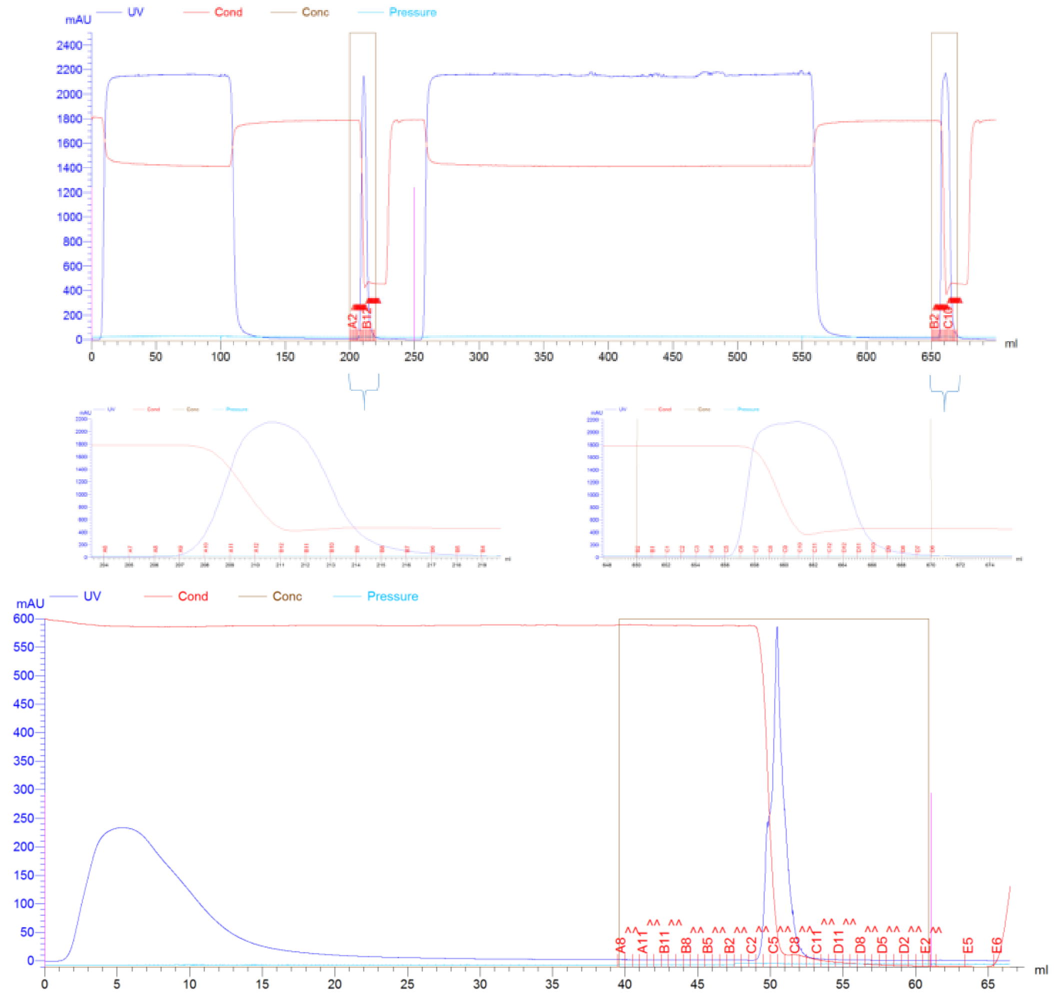

Figure S58. Traces (HiTrap MapSelecT SuRe column) for purification of IgG-K207A (upper and middle panel) or Fab-K207A (lower panel).

Cytotoxicity and IC_{50} calculation

Cytotoxicity of IgG and IgG-K207A conjugated with acetals **2**, **3**, and **4** was assessed using a CellTiter-Glo® Luminescent Cell Viability Assay (Promega), a method of determining the number of viable cells based on the quantitation of present ATP, an indicator of metabolically active cells. Compound **I** was used as a positive control. IgG, IgG-K207 and a DMSO solution were used as negative controls. Briefly, SKBR3 cells were seeded at a concentration of 10,000 cells/well (50 µL), in medium without antibiotics and without antimycotic) in flat-bottom 96 well-plates and allowed to adhere and adapt to the plates. The following day, the ADCs were filtered using a Millex GP 0.22µm unit, concentrated using 100K Amicon columns and diluted in culture medium without antibiotics and without antimycotic. Increasing concentrations of each ADC or controls (0.078 0.16, 0.31, 0.62, 1.2, 2.5, 5.0, 10 and 20 µM) were added to the medium in technical triplicates (50 µL), except unconjugated Thiomab that was done in duplicates. After 48 h incubation, cell viability was assessed by following CellTiter-Glo® Luminescent Cell Viability Assay protocol. Samples luminescence was detected with CLARIOstar® microplate reader (BMG Labtech), and relative luminescence units (RLU) were normalized to the values obtained for untreated cells. Results are shown as average of triplicates or duplicated. A sigmoidal curve (variable slope) was fitted to each dataset, using GraphPad Prism v9.3.1 software, and used to calculate the half-maximal inhibitory concentration (IC_{50}) for each ADC. Differences in IC_{50} values were evaluated with a Kruskal-Wallis test using GraphPad Prism v9.3.1 software. $p<0.05$ was considered statistically significant.

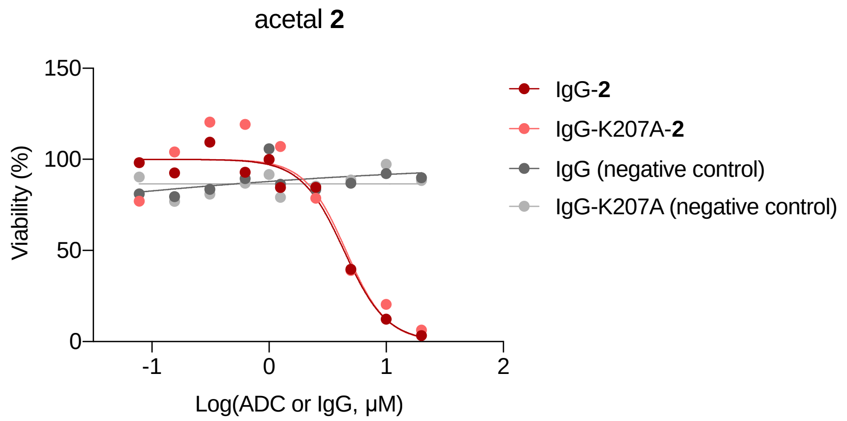

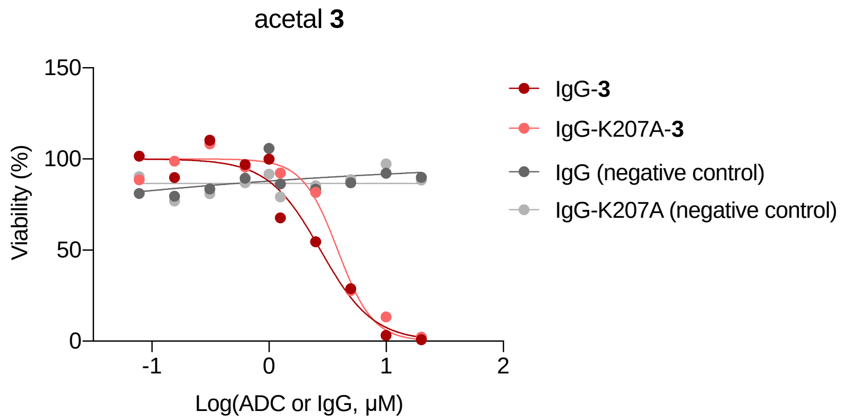

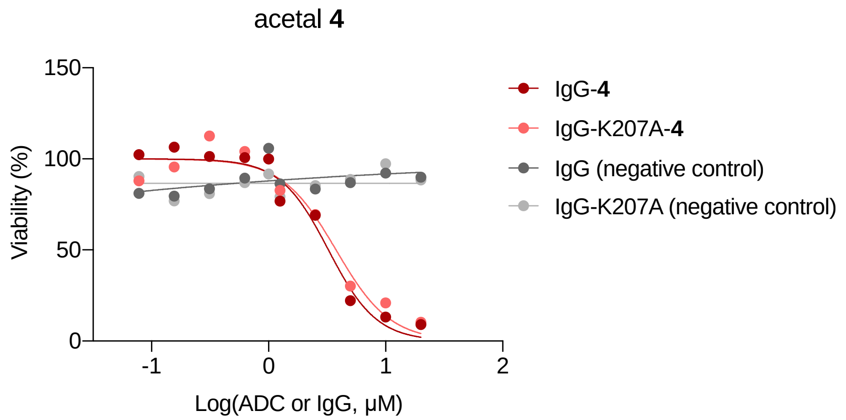

Figure S59. Dose-response curves of the different ADCs and unconjugated IgG (used as a negative control) in SKBR3 cells. IC_{50} values are shown in the main tex.

References

[1] S. Arumugam, V. V. Popik, *J. Am. Chem. Soc*. **2011**, *133*, 15730–15736.

[2] Gaussian 16, Revision C.01, M. J. Frisch, G. W. Trucks, H. B. Schlegel, G. E. Scuseria, M. A. Robb, J. R. Cheeseman, G. Scalmani, V. Barone, G. A. Petersson, H. Nakatsuji, X. Li, M. Caricato, A. V. Marenich, J. Bloino, B. G. Janesko, R. Gomperts, B. Mennucci, H. P. Hratchian, J. V. Ortiz, A. F. Izmaylov, J. L. Sonnenberg, D. Williams-Young, F. Ding, F. Lipparini, F. Egidi, J. Goings, B. Peng, A. Petrone, T. Henderson, D. Ranasinghe, V. G. Zakrzewski, J. Gao, N. Rega, G. Zheng, W. Liang, M. Hada, M. Ehara, K. Toyota, R. Fukuda, J. Hasegawa, M. Ishida, T. Nakajima, Y. Honda, O. Kitao, H. Nakai, T. Vreven, K. Throssell, J. A. Montgomery, Jr., J. E. Peralta, F. Ogliaro, M. J. Bearpark, J. J. Heyd, E. N. Brothers, K. N. Kudin, V. N. Staroverov, T. A. Keith, R. Kobayashi, J. Normand, K. Raghavachari, A. P. Rendell, J. C. Burant, S. S. Iyengar, J. Tomasi, M. Cossi, J. M. Millam, M. Klene, C. Adamo, R. Cammi, J. W. Ochterski, R. L. Martin, K. Morokuma, O. Farkas, J. B. Foresman, and D. J. Fox, Gaussian, Inc., Wallingford CT, 2016.

[3] Y. Zhao, D. G. Truhlar, *Theor. Chem. Acc*. **2008**, *120*, 215–241.

[4] G. Scalmani, M. J. Frisch, *J. Chem. Phys*. **2010**, *132*, 114110.

[5] R. F. Ribeiro, A. V. Marenich, C. J. Cramer, D. G. Truhlar, *J. Phys. Chem. B* **2011**, *115*, 14556–14562.

[6] C. Gonzalez, H.B. Schlegel, *J. Chem. Phys*. **1989**, *90*, 2154–2161.

[7] C. Gonzalez, H.B. Schlegel, *J. Phys. Chem*. **1990**, *94*, 5523–5527.

[8] M. Friedman, J. F. Cavins, J. S. Wall, *J. Am. Chem. Soc.* **1965**, *87*, 3672–3682.

[9] Mongan, J.; Case, D. A.; McCammon, J. A., *J. Comput. Chem.* **2004**, *25*, 2038–204.

[10] D.A. Case, S.R. Brozell, D.S. Cerutti, T.E. Cheatham, III, V.W.D. Cruzeiro, T.A. Darden, R.E. Duke, D. Ghoreishi, H. Gohlke, A.W. Goetz, D. Greene, R Harris, N. Homeyer, S. Izadi, A. Kovalenko, T.S. Lee, S. LeGrand, P. Li, C. Lin, J. Liu, T. Luchko, R. Luo, D.J. Mermelstein, K.M. Merz, Y. Miao, G. Monard, H. Nguyen, I. Omelyan, A. Onufriev, F. Pan, R. Qi, D.R. Roe, A. Roitberg, C. Sagui, S. Schott-Verdugo, J. Shen, C.L. Simmerling, J. Smith, J. Swails, R.C. Walker, J. Wang, H. Wei, R.M. Wolf, X. Wu, L. Xiao, D.M. York and P.A. Kollman (2018), AMBER 2018, University of California, San Francisco.

[11] Tsui, V.; Case, D. A., *Biopolymers* **2000**, *56*, 275–291.

[12] Onufriev, A.; Bashford, D.; Case, D. A., *Proteins* **2004**, *55*, 383–394.

[13] Hornak, V.; Abel, R.; Okur, A.; Strockbine, B.; Roitberg, A.; Simmerling, C., *Proteins* **2006**, *65*, 712–725.

[14] Uberuaga, B. P.; Anghel, M.; Voter, A. F., *J. Chem. Phys.* **2004**, *120*, 6363–6374.

[15] Sindhikara, D. J.; Kim, S.; Voter, A. F.; Roitberg, A. E., *J. Chem. Theory Comput.* **2009**, *5*, 1624–1631.

[16] Darden, T.; York, D.; Pedersen, L., *J. Chem. Phys.* **1993**, *98*, 10089–10092.

[17] H.-S. Cho, K. Mason, K. X. Ramyar, A. M. Stanley, S. B. Gabelli, D. W. Denney Jr, D. J. Leahy, *Nature* **2003**, *421*, 756–760.

[18] J. A. Maier, C. Martinez, K. Kasavajhala, L. Wickstrom, K. E. Hauser, C. J. Simmerling, *Chem. Theory Comput*. **2015**, *11*, 3696–3713.

[19] J. Wang, R. M. Wolf, J. W. Caldwell, P. A. Kollman, D. A. Case, *J. Comput. Chem*. **2004**, *25*, 1157–1174.

[20] C. I. Bayly, P. Cieplak, W. Cornell, P. A. Kollman, *J. Phys. Chem.* **1993**, *97*, 10269–10280.

[21] W. L. Jorgensen, J. Chandrasekhar, J. D. Madura, R. W. Impey, M. L. Klein, *J. Chem. Phys.* **1983**, *79*, 926–935.

[22] H. C. Andersen, *J. Chem. Phys*. **1980**, *72*, 2384–2393.

[23] S. Miyamoto, P. A. Kollman, *J. Comput. Chem*. **1992**, *13*, 952–962.

[24] J. McCafferty, K. J. Fitzgerald, J. Earnshaw, D. J. Chiswell, J. Link, R. Smith, J. Kenten, *Appl. Biochem. Biotechnol.* **1994**, *47*, 157–171; discussion 171–153.
